# Structure-Activity Study of an All-d Antimicrobial Octapeptide D2D

**DOI:** 10.3390/molecules24244571

**Published:** 2019-12-13

**Authors:** Abdullah Lone, Thomas T. Thomsen, Josefine Eilsø Nielsen, Peter W. Thulstrup, Rasmus N. Klitgaard, Anders Løbner-Olesen, Reidar Lund, Håvard Jenssen, Paul R. Hansen

**Affiliations:** 1 Department of Drug Design and Pharmacology, Faculty of Health and Medical Sciences, University of Copenhagen, Universitetsparken 2, 2100 Copenhagen, Denmark; alone@ruc.dk; 2 Department of Science and Environment, Roskilde University, 4000 Roskilde, Denmark; jenssen@ruc.dk; 3 Department of Clinical Microbiology, Rigshospitalet, Henrik Harpestrengs Vej 4A, 2100 Copenhagen, Denmark; thomas.thomsen@bio.ku.dk; 4 Department of Biology, Section for functional Genomics, University of Copenhagen, Ole Maaløes Vej 5, 2200 Copenhagen, Denmark; rasmusklitgaard49@gmail.com (R.N.K.); lobner@bio.ku.dk (A.L.-O.); 5 Department of Chemistry, University of Oslo, Sem Sælands vei 26, 0371 Oslo, Norway; j.e.nielsen@kjemi.uio.no (J.E.N.); reidar.lund@kjemi.uio.no (R.L.); 6 Department of Chemistry, University of Copenhagen, Universitetsparken 5, 2100 Copenhagen, Denmark; pwt@chem.ku.dk

**Keywords:** antimicrobial peptides, d-peptides, minimum inhibitory concentration, hemolytic activity, time-kill kinetics, circular dichroism, small angle X-ray scattering

## Abstract

The increasing emergence of multi-drug resistant bacteria is a serious threat to public health worldwide. Antimicrobial peptides have attracted attention as potential antibiotics since they are present in all multicellular organisms and act as a first line of defence against invading pathogens. We have previously identified a small all-d antimicrobial octapeptide amide kk(1-nal)fk(1-nal)k(nle)-NH_2_ (**D2D**) with promising antimicrobial activity. In this work, we have performed a structure-activity relationship study of **D2D** based on 36 analogues aimed at discovering which elements are important for antimicrobial activity and toxicity. These modifications include an alanine scan, probing variation of hydrophobicity at lys^5^ and lys^7^, manipulation of amphipathicity, N-and C-termini deletions and lys-arg substitutions. We found that the hydrophobic residues in position 3 (1-nal), 4 (phe), 6 (1-nal) and 8 (nle) are important for antimicrobial activity and to a lesser extent cationic lysine residues in position 1, 2, 5 and 7. Our best analogue **5**, showed MICs of 4 µg/mL against *A. baumannii*, *E. coli*, *P. aeruginosa* and *S. aureus* with a hemolytic activity of 47% against red blood cells. Furthermore, compound **5** kills bacteria in a concentration-dependent manner as shown by time-kill kinetics. Circular dichroism (CD) spectra of **D2D** and compounds **1**–**8** showed that they likely fold into α-helical secondary structure. Small angle x-ray scattering (SAXS) experiments showed that a random unstructured polymer-like chains model could explain **D2D** and compounds **1**, **3**, **4**, **6** and **8**. Solution structure of compound **5** can be described with a nanotube structure model, compound **7** can be described with a filament-like structure model, while compound **2** can be described with both models. Lipid interaction probed by small angle X-ray scattering (SAXS) showed that a higher amount of compound **5** (~50–60%) inserts into the bilayer compared to **D2D** (~30–50%). **D2D** still remains the lead compound, however compound **5** is an interesting antimicrobial peptide for further investigations due to its nanotube structure and minor improvement to antimicrobial activity compared to **D2D**.

## 1. Introduction

Multidrug-resistant (MDR) bacteria is a major global health problem [1]. Recently, WHO published a list of MDR bacteria which are of critical concern [2]. Especially worrying are the Gram-negative pathogens *Pseudomonas aeruginosa*, *Acinetobacter baumannii* and several species of Enterobacteriaceae [3]. The development of carbapenem resistance in these species has led to the re-introduction of the last resort antibiotic Colistin, which was initially abandoned due to side-effects. Since 2000, approximately 30 new antibiotics have been marketed worldwide of which five were first-in-class antibiotics [4]. However, these five new antibiotic classes linezolid, daptomycin, retapamulin, fidaxomicin and bedaquiline only target Gram-positive bacteria so there is an unmet need for new antibiotics targeting Gram-negative bacteria [4].

In recent years, antimicrobial peptides (AMPs) have attracted considerable interest as potential antibiotics [5]. Antimicrobial peptides are a part of the innate immune system in all higher organisms. They display broad-spectrum antimicrobial activity at low concentrations, fast killing, and often a membrane-specific mechanism of action [6]. Because the bacterial membrane is a fundamental part of the bacterial cell envelope, it is believed this makes it more difficult for microbes to develop effective mechanisms of resistance against AMPs as compared to traditional antibiotics [7]. Furthermore, AMPs may also have immunomodulatory properties [8]. The drawbacks of AMPs as peptide-based antibiotics include susceptibility to proteases and potential toxicity [9]. The stability of promising synthetic AMPs are typically improved by cyclization [10] or insertion of non-proteinogenic building blocks [11]. These include d-amino acids [12], peptoids (*N*-substituted glycines) [13], peptidomimetics such as, α-peptide/β-peptides [14], lysine-based α-peptides/α-peptoids [15], lysine-based α-peptide/β-peptoids [16], and α/γ *N*-Acylated-*N*-Aminoethylpeptides (AApeptides) [17]. Previously, we have identified an all-d-peptide, **D2D**, which shows promising activity against clinical isolates of Methicillin Resistant *Staphylococcus pseudintermedius* and *Pseudomonas aeruginosa*. Furthermore, the compound showed moderate toxicity against red blood cells and resistance to proteolytic degradation [18]. **D2D** consists of 4 cationic residues lys^1^, lys^2^, lys^5^, lys^7^ and 4 hydrophobic residues 1-naphthylalanine (1-nal)^3^, phe^4^, 1-nal^6^, norleucine (nle)^8^. **D2D** is a full d-amino acid analog derived from a structure-activity relationship study of the peptoid **D2**. Furthermore **D2**, was selected in a previous study after screening from a combinatorial library of de novo designed compounds for their activity against *S. pseudintermedius* and toxicity in vitro against red blood cells [19].

**Figure 1:**
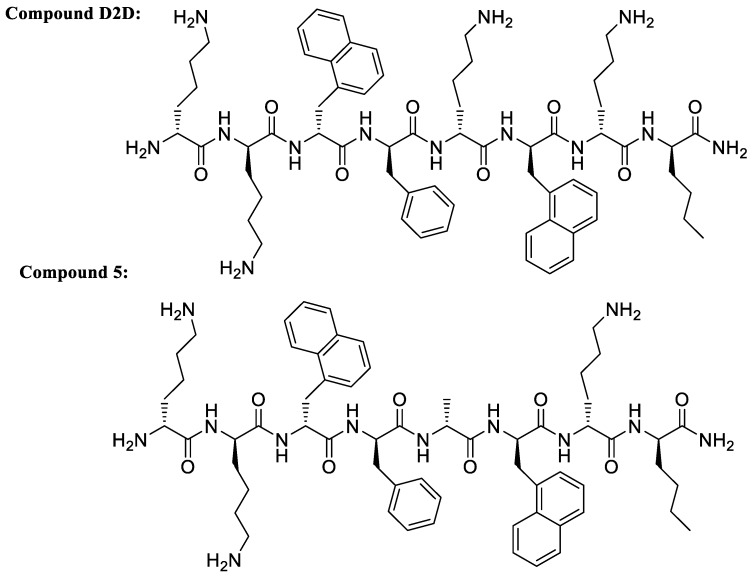
Chemical structure of the parent compound **D2D** and the best analogue compound **5**.

In this work, we present a structure-activity study of **D2D** (see Figure 1) based on 36 analogs including ala-scan, N-and C-termini deletions and lys-arg replacements. The analogs were tested against *Escherichia coli* (ATCC 25922), *Staphylococcus aureus* (ATCC 29213), *Pseudomonas aeruginosa* (ATCC 27853) and *Acinetobacter baumannii* (ATCC 19606).

## 2. Results and Discussion

A total of 37 peptides including the lead compound **D2D** (Table 1) were synthesized. See supplementary materials for structures, HPLC chromatograms and MALDI-TOF-MS spectra.

### 2.1. Alanine Scan of ***D2D***

In order to evaluate the role of each individual amino acid for the antimicrobial activity and toxicity of **D2D**, we performed alanine scan by systematically replacing all residues with d-alanine (Table 1). This identified four residues to be important for activity (compounds **3** (1-nal), **4** (phe), **6** (1-nal) and **8** (nle) (Table 1)). When these residues were replaced with d-alanine the MIC changed from 4–8 µg/mL to between 64 and >128 μg/mL for *E. coli* and *S. aureus* while *P. aeruginosa* and *A. baumannii* ranged from 16 to > 128 μg/mL. The 1-naphtylalanine (1-nal) at position 6 appeared to be the most important amino acid in the sequence for maintaining activity. These substitutions (**3**, **4**, **6** and **8**) also decreased the hemolytic activity compared to **D2D**. The alanine scan identified four of eight residues (Compounds **1**, **2**, **5** and **7**, all lys) with improved or similar MIC values compared to **D2D**. The MIC values ranged from 4–16 μg/mL for *E. coli*, *P. aeruginosa*, *A. baumannii* and 2–8 μg/mL for *S. aureus*. Compound **1** improved the activity against *S. aureus* by 4-fold, but decreased activity against *P. aeruginosa* by 2-fold compared to **D2D**. Compound **2** had decreased activity against *E. coli*, *P. aeruginosa* and *A. baumannii* by 2-fold, but retained the activity against *S. aureus* compared to **D2D**. Compound **5** showed improved activity against *E. coli* and *S. aureus* and comparable activity to **D2D** against *P. aeruginosa* and *A. baumannii*.

Compound **7** showed a 2-fold reduction in MIC against *P. aeruginosa*, but retained activity against other tested bacteria compared to **D2D**. Compound **1** and **5** showed higher hemolytic activity compared to **D2D**. Compound **2** and **7** greatly increased the hemolytic activity compared to **D2D**.

As mentioned previously, an ala-scan is often used to identify the importance of the side-chain in antimicrobial peptides. Representative examples include indolicidin [20], Temporin 1AI [21] and anoplin [22]. What is typically found is that cationic residues like Lys or Arg are replaceable, while substitution of hydrophobic residues has a great negative effect on MIC but a positive effect on hemolytic activity.

In an ala-scan of the α-helical decapeptide amide anoplin, GLLKRIKTLL, replacing residue Arg^5^, Lys^7^ or Thr^8^ with alanine were found to be more active than anoplin by extending the hydrophobic phase of the helix [22], while substituting the five hydrophobic residues resulted in increased MICs. In the present study, we investigated all-d-peptides. Previously, we have shown that **D2D** is approximately four-fold more active than its l-enantiomer [18]. This is in alignment with several studies which report that the d-enantiomer is more active than the corresponding l-peptide. For example, Manabe et al. compared the antimicrobial activity of a sapesin B analog, KLKLLLLLKLK-NH2, to its corresponding all-d analogue [23]. A significantly higher antimicrobial activity against *S. aureus* was found for the d-peptide (1 and 16 µg/mL, respectively). Similarly Oddo et al. reported that an all d-peptide amide, kklfkkilryl was significantly more active against *A. baumannii* than the corresponding l-enantiomer [24].

### 2.2. Investigation of Hydrophobicity at Position 5 and 7 Analogues

**Figure 2:**
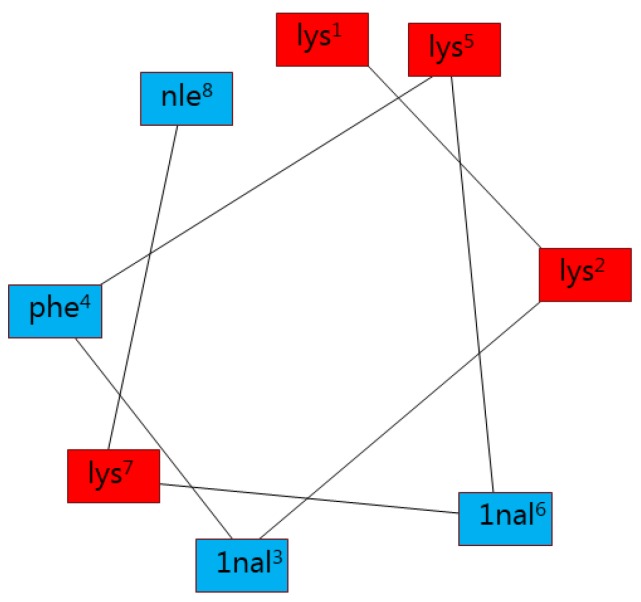
Helical wheel of **D2D**. The red colour indicates cationic amino acids and the blue colour indicates hydrophobic amino acids.

A series of substituted analogues were designed based on the above results. These included substitutions at residue lys^5^ and lys^7^ based on the improved or retained antimicrobial activity of compound **5** and **7** compared to **D2D**. Furthermore, placing **D2D** on a Schiffer-Edmundson wheel projection [25] (Figure 2) revealed a partly amphipathic helix, with lys^1^, lys^5^, and lys^2^ on the hydrophilic surface of the helical wheel and 1nal^6^, 1nal^3^, lys^7^, phe^4^, and nle^8^ on the hydrophobic side. The amino acid substitutions were selected to study the effects of substituting more or less hydrophobic amino acids than alanine. The chosen amino acids were d-1-Naphthylalanine, d-Dap, d-Nle, d-Phe, d-Ser, d-Thr, d-Tyr, and d-Val.

Position 5 (Compounds **9**–**16** Table 1): The MIC values for position 5 substitutions ranged from 4–64 μg/mL for *E. coli*, 2–16 μg/mL for *S. aureus*, 16–128 μg/mL for *P. aeruginosa* and 8–64 μg/mL for *A. baumannii*. The substitutions at position 5 did not improve the MIC values overall compared to **5** and **D2D**, but some peptides had improved activity against *S. aureus*. Substitutions at position 5 generally increased the hemolytic activity compared to **D2D** (23% hemolysis) compounds **11**, **12**, **15**, and **16** being more hemolytic than **D2D** and compound **9**, **10**, **13**, and **14** having moderate hemolytic activity compared to **D2D** at 150 μM.

Position 7 (Compounds **17**–**24** Table 1): The MIC values for position 7 substitutions ranged from 8–16 μg/mL for E. coli, 2–16 μg/mL for *S. aureus*, 16–64 μg/mL for *P. aeruginosa* and 4–16 μg/mL for *A. baumannii*. The substitutions at position 7 (compounds **17**–**24**) did not improve the MIC values overall compared to compound **5** and **D2D**, but the antimicrobial activity of the position 7 peptides is overall better compared to position 5 substitutions (compounds **9**–**16**). The substitutions at position 7 have significantly larger hemolytic activity compared to position 5 substitutions, with **18** being the only peptide with a hemolytic activity below 50% at 150 μM.

### 2.3. Manipulating Amphipaticity and Truncations (Compounds ***25***–***32**)*

Compound **25** and **26**:

Since many small cationic antimicrobial peptide adopt α-helical structures, we designed compounds **25** and **26** assuming possible gain from creating more balance between the hydrophobic and cationic residues. To obtain a better balance between these domains we exchanged 1-nal^6^ with lys^7^ compound **25** and lys^7^ and nle^8^ analogue **26** (Figure 2). In both cases, this creates a perfect amphipathic helix. The MIC values for both **25** and **26** were 128 µg/mL for *E. coli*, 64 µg/mL for *S. aureus* and 32 µg/mL for *P. aeruginosa* and *A. baumannii*. These rearrangements in the sequence lead to a significant loss in activity. The hemolytic activity was increased for compound **25** (50%), while compound **26** (18%) is almost the same as **D2D** (23%). The difference in hemolytic activity could be explained by the cationic lys^8^ at the C-terminal end for compound **26**.

N- and C-terminus truncations abolish activity of **D2D** (Compounds **27**–**32** Table 1):

The influence of the N- and C-terminal regions for the antimicrobial activity and toxicity were investigated. Six truncated analogues of **D2D** were synthesized; compounds **27**–**29** being the N-terminal truncations and compounds **30**–**32** being the C-terminal truncations. Only [des-lys^1^]**D2D**, compound **27**, showed some activity. Our finding that truncated analogues are not active is agreement with other structural activity studies of AMPs, e.g., fallaxin [26] and anoplin [22].

### 2.4. Manipulating Cationicity by Lys-Arg Substitutions (Compound ***33***–***36***)

The influence of an increased cationicity for the antimicrobial activity and toxicity was investigated by replacing all lysines one at a time with arginine. Arginine forms three hydrogen bonds (pKa 12.5) compared to lysine which forms two (pKa 10.5). The MIC values for the arginine substitutions was 8 μg/mL for *E. coli*, 2–8 μg/mL for *S. aureus*, 16 μg/mL for *P. aeruginosa* and 8–16 μg/mL for *A. baumannii*. The antimicrobial activity did not improve, but was more or less retained for the arginine substitutions compared to **D2D**. The hemolytic activity for analogues **33**, **34**, **35**, and **36** increased compared to **D2D**. A study of the antimicrobial peptide amide BP100, KKLFKKILKYL, showed that a single substitution with arginine at lys^9^ improved antimicrobial activity without any detectable increase in toxicity [24]. Another study of antimicrobial peptides consisting of exclusively arginine and tryptophan of varying length showed that arginine compared to lysine could yield some antimicrobial peptides with enhanced antimicrobial activity [27]. However, improvements in the antimicrobial activity was not seen for **33**–**36**.

### 2.5. Time-kill Kinetics of ***D2D*** and Analogue 5

**Figure 3:**
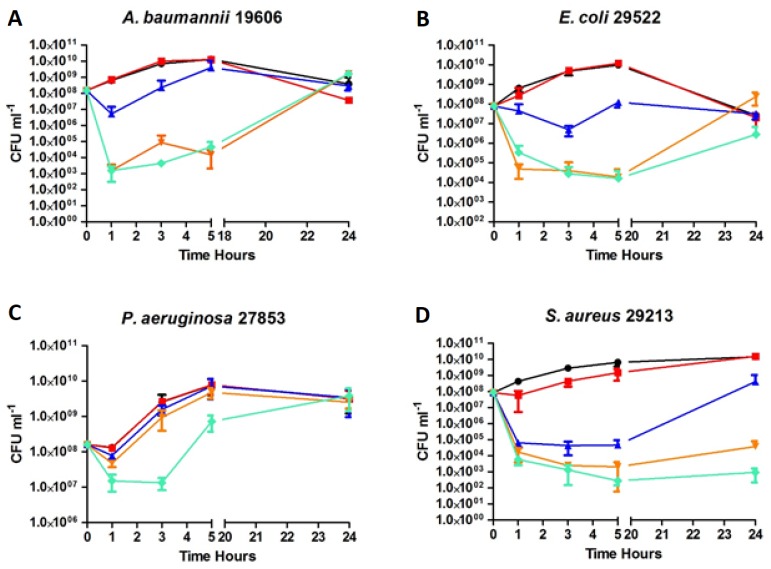
Time-Kill Kinetics. Compound **5** (**C5**) and **D2D** were evaluated for in vitro efficacy in time kill experiments. Exponentially growing cells (1 × 10^8^ cells) of ATCC strains *A. baumannii* 19606 (**A**), *E. coli* 25922 (**B**), *P. aeruginosa* 27853 (**C**) and *S. aureus* 29213 (**D**) were treated with **C5** at 1 × MIC (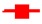), 3 × MIC (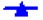), 5 × MIC (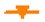) (MIC = 4 µg/mL), **D2D** at 5 × MIC (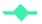) and non-treated cells (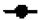). Sampling of viable cells was performed at time points 0, 1, 3, 5 and 24 h (X-axis). Viable cell counts (CFU ml^−1^, Y-axis), was done by spot plating of washed cells.

Compound **5** (**C5**) and **D2D** were evaluated for in vitro efficacy in time kill experiments (Figure 3). They were tested against ATCC strains: *A. baumannii* (Figure 3A), *E. coli* (Figure 3B), *P. aeruginosa* (Figure 3C) and *S. aureus* (Figure 3D). Compound **5** was tested at concentrations of 1 × MIC, 3 × MIC and 5 × MIC in all experiments. For comparison **D2D** was only included at 5 × MIC which seems to be slightly more active than compound **5**. There is a clear concentration-dependent bacterial killing observed for compound **5**, as is often seen for cationic AMPs [11]. **D2D** and compound **5**, both reduce the number of viable cells by 4–5 logs against *E. coli* and *A. baumannii* at 5 × MIC (Figure 3A,B). Whereas, only **D2D** is capable of killing 1 log of *P. aeruginosa* at 5 × MIC (Figure 3C), clearly demonstrating some differentiation in species specificity between the compounds. For the three Gram-negative species tested, regrowth of cultures was evident after 24 h. This is probably related to the concentration dependent killing, as the peptides get sequestered by killed cells leaving unaffected cells to regrow. Both compounds were shown to be capable of killing *S. aureus* with a 5 log reduction in viable cells at 5 × MIC. From the data, it seems that there could be a slight re-growth of *S. aureus* after 24 h, however it is much less pronounced than it was for the Gram-negative species, indicating that bacterial killing probably was more pronounced. Because the MIC of compound **5** was the same for all species (4 µg/mL), this indicates that the concentration dependent killing is differentiated from Gram-negative to Gram-positive bacteria, i.e., less compound is needed for antimicrobial killing of Gram-positive *S. aureus* or less is sequestered than for Gram-negative species. Furthermore, it is clear that *P. aeruginosa* is much less sensitive to compound **5** and **D2D** in our time kill experiments, indicating that while the MIC is relative similar against all Gram-negative species tested (at 5 × 10^5^ CFU), growth conditions at higher cell densities in exponentially growing cultures may influence activity/sequestration. Our data also indicate that small modifications to AMPs (Table 1) can have relatively large effect on species-specific activity as seen for *P. aeruginosa*. However, this is only indicative and it needs a more detailed analysis.

### 2.6. CD-Experiments 

**Figure 4:**
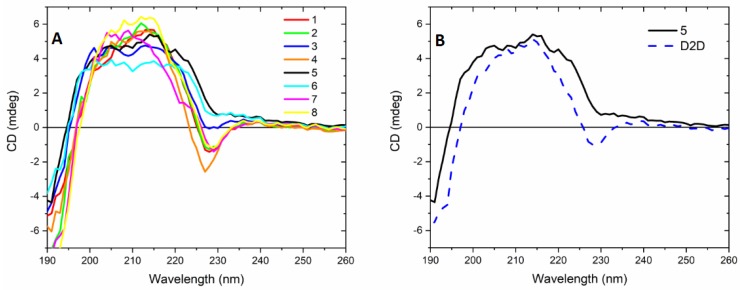
Far-UV circular dichroism spectra of **D2D** and **1**–**8** in a 50% trifluoroethanol water mixture at 37 °C. The left panel compares **1**–**8** (**A**) while the right panel compares **5** to **D2D** (**B**).

**Figure 5:**
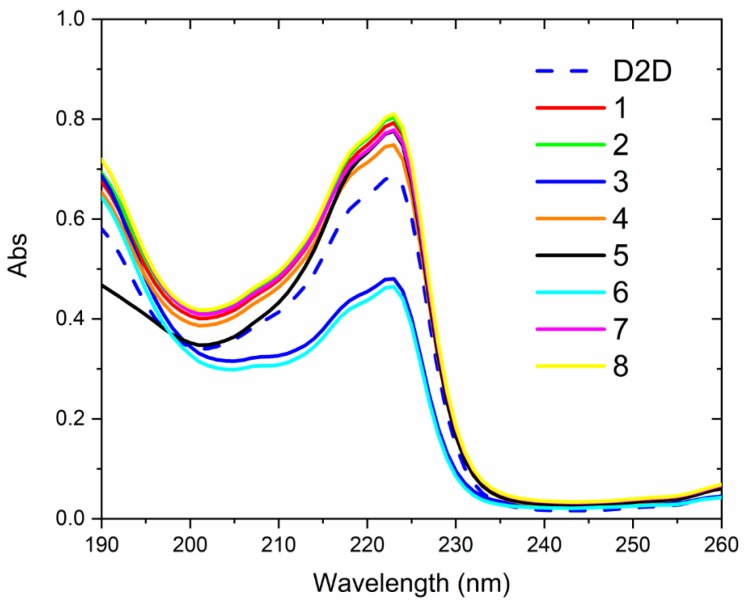
Far-UV absorption spectra for **D2D** and **1**–**8** in a 50% trifluoroethanol water mixture at 37 °C. as obtained from conversion of the high tension signal of the detector during measurement of CD. The absorbance peak with maximum at 223 nm originates in the naphthylalanine side chain, which is present at positions three and six (except in compound **3** and **6** where one such instance of 1-nal is replaced by ala).

The solution structure of compounds **1**–**8** were compared with the **D2D** sequence by circular dichroism spectroscopy at 37 °C in a simple membrane-mimicking solvent mixture of water and trifluoroethanol (Figure 4). **D2D** and all eight analogs display far UV CD that can point toward folding with α-helical or possibly β-sheet secondary structure, but a detailed analysis is hindered due to the strong spectral contributions from the aromatic 1-naphtylalanine sidechains. The positive CD in the range from 200–240 nm and negative below 200 nm for **1**–**8** all is consistent with the expected mirror image spectra of an l-amino acid sequence with α-helical secondary structure [28]. The alanine scan has consequences for the UV absorption due to the single (in 3 and 6) or double presence of the chromophoric sidechain of naphthylalanine (in the rest) which displays an absorbance band centered at 223 nm (Figure 5). The far-UV CD spectra are also affected by these aromatic side-chains. This is mainly observed in the highest wavelength region, between 220–240 nm, where a negatively signed component is seen for peptides with two d-1-naphthylalanines present (Figure 4A), which is likely due an exciton coupling effect between the aromatic groups. The exception to this is observed for compound **5**, which does not display a first negative band component, despite the sequence similarity to **D2D** (Figure 4B). This might be interpreted as a case where the positive peak of the peptide n-π * transition eliminates a contribution from the d-1-naphthylalanines− or that these have a changed signature due to a difference in folding of **5** compared to e.g., **D2D**.

### 2.7. Peptide Self-Assembly Nanostructure Investigated by Small Angle X-ray Scattering

**Figure 6:**
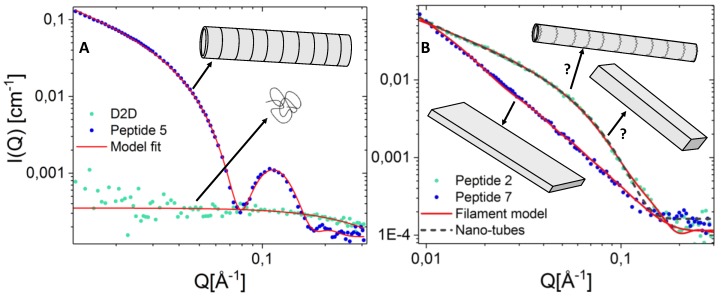
SAXS results showing the normalized scattered intensity of 5 mg/mL peptides in solution measured by a Bruker Nanostar SAXS instrument. Comparison of scattering from compound **D2D** and **5** (**A**) and compound **2** and **7** (**B**) in solution with model fits and inset illustration representing the assumed structure based on model fit analysis.

The nanostructure of peptide **D2D** and compound **1**–**8** (alanine-scan) in solution was studied in detail using SAXS at ambient temperature. In Figure 6A the scattering curve from the lead compound **D2D** and the most active compound **5** is plotted together for comparison. As seen from the figure, there are significant differences in the structure of these two peptides. While the scattering curve for **D2D** can be recognized as the classical scattering pattern expected from random unstructured polymer-like chains, the results for compound **5** indicates formation of much larger self-assembled structures. Fit analysis of the data from compound **5** reveals that the scattering can be well explained using a fit model of defined hollow nano-tubes with an outer radius of 33 Å and a shell thickness of 4 Å. The structure is retained over the whole concentration range from 2.5–10 mg/mL (lower concentrations were not measured as the scattering signal to noise ratio is too low for the lab-SAXS instrument and the peptide too susceptible to radiation damage to be measured using synchrotron SAXS). Increasing the temperature to 37 °C and 45 °C did not dissolve the structure (see supplementary information Figure S1).

Further studies showed that compounds **1**, **3**, **4**, **6** and **8** all resemble **D2D** as random polymer-like chains (see supplementary information, Figure S3), while compounds **2** and **7** assemble into larger filament like structures as seen in Figure 6B. The scattering curve for compound **2** can be explained by a nanotube structure where the cross section of the tubes is much smaller (14 Å) than for compound **5**. The scattering could also be explained by a filament-like structure with dimensions 65x32x > 400 Å consisting of 85% water. Compound **7** on the other hand does not fit with a nano-tube model but has a higher slope at low q (power of 2) indicating sheet-formation. Due to the limited q range on the lab-source SAXS instrument a defined length cannot be directly accessed from the scattering curve.

The detector image (see supplementary information Figure S2) reveal that compound **2** filaments aligns parallel in the capillary resulting in anisotropic scattering (seen as asymmetric beam on the detector). The sample was measured for 14 h to see whether the injection into the capillary was the cause of the filament-ordering. However, the results revealed that the asymmetry of the scattering on the detector increased over time. This indicate that during the peptide injection the filaments are partly broken up, and reforms in the capillary over time seen as an increased asymmetric scattering pattern. However as seen in Figure S2 the scattering curve does not change over time indicating that the increase in length cannot be seen in the probed q-range (the “broken up” filaments at time = 0 are already above 400 Å.).

Even though both compound **5** and **7** both have the same elongated structure these peptides do not align in the capillary during the measurements in the same way. This effect might be explained by increased length of the tubes or mechanical stiffness for compound **2** compared with compound **5** and **7**. However, as the exact length of these peptides is not visible in the available q-range and the mechanical properties have not been studied, this remains inconclusive.

### 2.8. Lipid Interaction Probed by Small Angle X-ray Scattering

**Figure 7:**
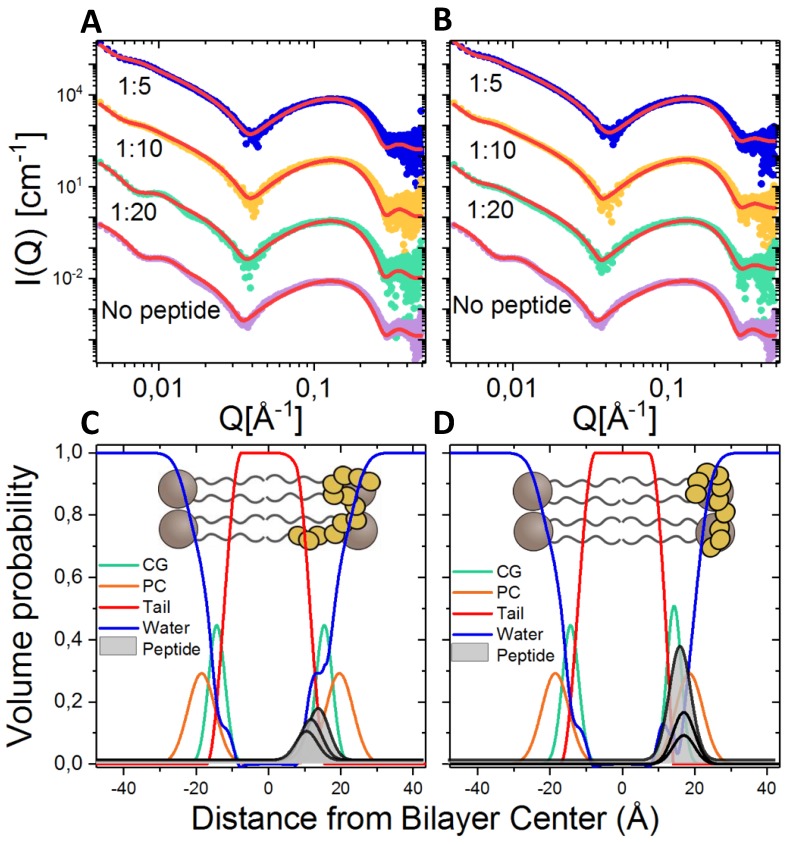
Lipid-peptide interaction of **D2D** and **5** studied using synchrotron SAXS, and the resulting lipid bilayer structure (of DMPC-DMPG-DMPE-PEG bilayers) as obtained from detailed mathematical modeling of the scattering curves. Scattering of compound **D2D** (**A**) and compound **5** (**B**) mixed with lipids in ration 1:5, 1:10 and 1:20 together with the model fit curves in red. The curves have been offset with a factor of 100 for better visualization. Based on the fit analysis volume probability plots have been calculated for **D2D** (**C**) and compound **5** (**D**) showing that a higher amount of compound **5** inserts into the membrane than **D2D**. Both peptides seem to position in the interface between the headgroups and tail region of the outer leaflet of the lipid bilayer.

As bacterial membrane destruction is reported as one of the most important modes of actions of antimicrobial peptides we mixed the most active compound **5** and the lead compound **D2D** with lipid vesicles mimicking membranes of bacterial and measured the samples with synchrotron-SAXS (see Figure 7A,B).

The results were analysed using a detailed scattering model that allow extraction of peptide position in the bilayer as well as the peptide effect on the structure and thickness of the lipid bilayer [29]. From the fit parameters (see supplementary information Table S2) a volume probability plot showing the structure of the bilayer after peptide insertion can be calculated, and has been plotted in Figure 7C for peptide **D2D** and Figure 7D for compound **5**. As seen from the plot a higher amount of compound **5** (~50–60%) inserts into the bilayer compared to **D2D** (~30–50%). This is seen directly from the scattering curve in Figure 7A,B by a smaller shift in the first minima at intermediate q for **D2D**. When comparing to previous results found for natural unstructured peptides like indolicidin, where ~75–100% of the peptide has been found to insert into bilayers of the same lipid composition [29], the synthetic d-peptides in this study have a slightly lower affinity for the membrane.

Further **D2D** does not seem to affect the membrane structure in any significant way at this concentration, even at higher ratios where only minor reductions of the bilayer thickness of about 1 Å was found which is within statistical errors. These results indicate that there is no evidence for membrane deformation or any particular indication of pore formation. However peptide membrane insertion has been suggested to cause lipid disordering resulting increased leakage of cell fluids [30].

## 3. Materials and Methods

TentaGel R RAM (0.19 mmol/g) were purchased from Rapp Polymere GmbH. TFA (trifluoroacetic acid) and piperidine were purchased from Iris-Biotech GmbH. Fmoc (9-fluorenylmethyloxycarbonyl) protected amino acids were purchased from Sigma-Aldrich, Iris-Biotech GmbH, Novabiochem and Alfa Aesar. Disposable reactors (5 mL polypropylene) fitted with a PTFE filter were acquired from Fa. Gerhardt, Kassel Germany. *N*,*N*-diisopropylethylamine (DIEA), triisopropylsilane (TIS), Mueller-Hinton Broth II (MHBII), Phosphate buffered saline (PBS tablets) and α-cyano-4-Hydroxycinnamic acid (ACCA) were from Sigma-Aldrich. 1-Hydroxy-7-azabenzotriazole (HOAt) and (1-[Bis(dimethylamino)methylene]-1*H*-1,2,3-triazolo[4,5-b]pyridinium 3-oxid hexafluorophosphate (HATU) were from GL Biochem Shanghai. Melittin were from Serva. DMF (dimethylformamide), DCM (dichloromethane), ACN (acetonitrile) and Et_2_O (diethyl ether) were from VWR, Pennsylvania, USA. All reagents and solvents were used without further purification. Biorad Microseal film, CAPP Origami reagent reservoir, clear V and U-shaped 96-well polypropylene plate, clear flat bottom 96-well polystyrene ELISA plate, Eppendorf Protein LoBind tubes (2 mL).

### 3.1. Synthesis of Peptides

The manual Fmoc solid-phase synthesis of peptides was carried out in disposable syringes, equipped with a fritted filter [31]. Crude products were purified by preparative RP-HPLC until ≥95% purity was obtained (analytical RP-HPLC). HPLC system consisting of WatersTM 600 Pump, In-line Degasser, 600 Controller and 2996 Photodiode Array Detector, the column used was a WatersTM XBridgeTM BEH130 C18, 5 µm, 10 × 250 mm with H_2_O:ACN gradient. The appropriate fractions were concentrated and lyophilized. Purity was determined by analytical reverse-phase HPLC system consisting of WatersTM 717 plus Autosampler, In-line Degasser AF, 600 Controller and 2996 Photodiode Array Detector, the column used was a WatersTM SymmetryTM C18, 5 µm, 4.6 × 250 mm on an acetonitrile-water gradient. Finally, the products were characterized by matrix-assisted laser desorption/ionization time-of-flight mass spectrometry (Bruker MicroflexTM), using α-cyano-4-hydroxycinnamic acid as matrix.

### 3.2. Bacterial Strains

*E. coli* (ATCC 25922), *S. aureus* (ATCC 29213), *P. aeruginosa* (ATCC 27853) and *A. baumannii* (ATCC 19606).

### 3.3. Antimicrobial Susceptibility Testing

Antimicrobial susceptibility testing was performed by broth dilution in MHB-II media using a protocol adapted from [32], with minor modifications. Briefly, the compounds were prepared in standard stocks of 1 mg/mL in milli-Q H_2_O. Antimicrobial dilutions were prepared in two times the final concentration (128 µg/mL) of the compounds. Bacterial suspensions were prepared from overnight cultures (MHB-II) by diluting to OD600 = 0.001 (Approximately 1 × 10^6^ CFU). An amount of 100 µL MHB-II was transferred to all wells in column 1–9, excluding the antibiotics well (A1–A9). An amount of 200 µL of MHB-II was transferred to all wells in column 10–12. An amount of 200 µL peptide stock was transferred to each well from A1–9. A 2-fold serial dilution from row A–G was obtained by transferring 100 µL from row A to B, and continued until row G. Resulting in final concentration range of peptides: 128, 64, 32, 16, 8, 4, and 2 (µg/mL). Finally, 100 µL bacterial suspension (1 × 10^6^ CFU) were added to all wells giving a final bacterial inoculum of approximately 5 × 10^5^ colony forming units (CFU). The plates were incubated for 18–20 h at 37 °C.

### 3.4. Time Kill Curves

The time kill experiments were performed by treating exponentially growing cultures with **D2D** and compound **5**. Overnight cultures grown in MHB-II media, were diluted 1:10.000 to ensure exponentially growing cultures (8–10 generations), before cultures reached 1 × 10^8^ CFU approximately OD600 = 0.1. When cultures reached 1 × 10^8^ they were split into blood glass tubes containing 3 mL each and then treated with the respective drugs at the concentrations as shown in Figure 3. Sampling was performed at 0, 1, 3, 5 and 24 h, by removing 250 µL culture. Samples were spun down at 4000 G for 5 min and supernatant removed, cells were washed in 1 mL 0.9% NaCl, spun down again, before being suspended in 250 µL 0.9% NaCl. Finally, 10 fold dilution series were prepared in 0.9% NaCl. From these 10 µL were spotted (in triplicate) onto LB agar. CFU were counted after 24 h incubation at 37 °C. Experiments were performed in triplicate from individual colonies. Data analysis was performed using GraphPad prism 5 and plotted as the mean of triplicated with standard deviation.

### 3.5. Hemolytic Activity

The percentage of hemolysis at 150 μM was determined for all compounds as previously described [33]. Briefly, two-fold serial dilutions (2.35 to 150 μM) of compounds in PBS were mixed with a 0.5% *v*/*v* suspension of fresh human red blood cells (RBC) in the same buffer. After 1 h incubation at 37 °C, plates were centrifuged and aliquots of the supernatants were transferred to clear ELISA plates. Absorbance at 414 nm was determined and normalised with respect to a negative (PBS, 0%) and positive (melittin, 100%) control.

### 3.6. Circular Dichroism

Circular dichroism (CD) spectra were measured on a Jasco J-815 spectropolariometer in the far-UV region from 260–190 nm with a 1 nm interval, a 2 nm bandwidth using a digital integration time of 4 s and a scan speed of 20 nm per min. During measurement, the high-tension signal applied to the detector was also recorded and was subsequently converted to absorbance. Data represents the average of five individual scans subtracted a corresponding reference measurement on pure solvent. The peptide samples **D2D** and **1**–**8** were dissolved in milli-Q water with 50% (*v*/*v*) 2,2,2-trifluoroethanol (TFE). Each sample was placed in a 2 mm quartz cuvette from Hellma. The temperature was kept at 37 °C during measurements using a Jasco CDF-426S thermostated sample holder accessory. Data was treated using Jasco Spectra Analysis and plotted using MicroCal OriginPro 2018. No smoothing has been applied. The quantitative interpretation of secondary structure for the studied peptides is hampered by the spectral contributions of the aromatic sidechains and the analysis has thus centered on a comparative study of the band shape and sign and the Y-axis unit is reported as the measured elipticity signal in mdeg.

### 3.7. Small Angle X-ray Scattering on Pure Peptides in Solution

SAXS experiments of **D2D** and the alanine-scan (compounds **1**–**8**) were performed using a Bruker NANOSTAR equipped with a microfocus X-ray source (IμS Cu, InCoatec, Germany) and a VÅNTEC-2000 detector. Raw scattering data was calibrated to absolute intensity scale using water as a primary standard and radially averaged in order to obtain the 1D scattered intensity profile as a function of the scattering vector *q* (*q* = 4 π sin (*θ*/2)/*λ*), where *θ* is the scattering angle and *λ* is the X-ray wavelength of 1.54 Å. The samples were dissolved in 50 mM Tris buffer, pH 7.4 (10 mg/mL, 5 mg/mL and 2.5 mg/mL) and injected manually into a temperature-controlled quartz cell located in a vacuum chamber and measured for 180 min.

Random polymer like chains:

The scattering of the peptide **D2D** was analysed using a random polymer-like chain model:

(1) $$I_{\mathrm{chain}}\left(q\right)=\phi \cdot V_{p}\cdot \Delta \rho^{2}\cdot P_{\mathrm{chain}}\left(q\right)$$

where ϕ is the volume fraction of the peptide, $V_{p}$ is the volume of the polymer, Δρ is the excess scattering length density and *P_chain_*(*q*) is the form factor of the free peptide chains given by the Debye expression for Gaussian chains:

(2) $$gP_{\mathrm{chain}}\left(q\right)=\frac{2\cdot \exp\left[-{\left(qR_{g}\right)}^{2}\right]-1+{\left(qR_{g}\right)}^{2}}{{\left(qR_{g}\right)}^{4}}$$

where *R_g_* is the radius gyration of the peptide chains.

Filament like sheet model:

The scattering for compounds **2** and **7** was analysed using a rectangular sheet like model:

(3) $$I_{\mathrm{sheet}}\left(q\right)=\phi \cdot V_{p}\cdot \Delta \rho^{2}\cdot N_{p}\cdot P_{\mathrm{sheet}}\left(q\right)$$

Under the assumption that the length of the peptide sheets are much greater than the lateral dimension, i.e., c >> a, b, the form factor *P_sheet_*(*q*) is given by

(4) $$P_{\mathrm{sheet}}\left(q\right)=F_{c}\left(q\right)\frac{1}{2\pi }\int_{0}^{2\pi }A_{\mathrm{sheet}}{\left(q,\;\alpha \right)}^{2}d\alpha$$

where the amplitude is given by

(5) $$A_{\mathrm{sheet}}\left(q,\alpha \right)=\frac{\sin\left(qb\;\cos\left(\alpha \right)/2\right)}{qb\;\cos\left(\alpha \right)/2}\cdot \;\frac{\sin\left(qa\;\sin\left(\alpha \right)/2\right)\;}{qa\;\sin\left(\alpha \right)/2)}$$

and

(6) $$F_{c}\left(q\right)=(2\;\mathrm{Si}\left(qc\right)/\left(qc\right)-4{\;\sin}^{2}\left(qc/2\right)/{\left(qc\right)}^{2}$$

where $\mathrm{Si}\left(x\right)=\int_{0}^{x}t^{-1}\sin t\;\mathrm{dt}$.

Nanotube model:

The expression for the absolute scattering intensity, from this structure can be written as [34]:

(7) $$I_{nanotube}\left(q\right)=\phi \cdot P\cdot V_{tot}\cdot \Delta \rho^{2}\cdot P{\left(q\right)}_{L}\cdot A{\left(q\right)}_{cs}^{2}\cdot DW\left(q\right)$$

where ϕ is the volume fraction of the peptide, P is the aggregation number given by $P\;\pi \left(R_{o}^{2}-R_{i}^{2}\right)L/V_{p}$ where $V_{p}$ is the volume of a peptide chain, $R_{o}$ is the outer radius and $R_{i}$ is the inner radius of the tubes, and L is the length. $A{\left(q\right)}_{cs}^{2}$ is given by:

(8) $$A{\left(q\right)}_{cs}^{2}=\frac{R_{o}^{2}A\left(q,R_{c}\right)-R_{in}^{2}A\left(q,R_{i}\right)}{\left(R_{o}^{2}-R_{i}^{2}\right)}$$

where $A\left(q,x\right)=2J_{1}\left(q\cdot x\right)/\left(q\cdot x\right)$ and $J_{1}\left(x\right)$ is the first order Bessel function.

The longitudinal form factor *P*(*q*)*_L_* is given by:

(9) $$P{\left(q\right)}_{L}=2\;\mathrm{Si}\left(QL\right)/QL\;-4\;\sin(sin^{2}\left(\frac{QL}{2}\right)/{\left(QL\right)}^{2}$$

where *L* is the length of the nanotubes and Si(x) is the Sine-function.

In addition, we introduced a slight distribution in the inner radius for possible shape fluctuations, and a Debye Waller factor, *DW*(*q*) = exp(−*q*^2^ *σ*^2^), describing possible surface roughness of the inner and outer wall.

Synchrotron-small angle x-ray scattering on peptide-lipid interaction.

For preparation of unilamellar lipid vesicles, synthetic DMPC (1,2-dimyristoyl-sn-glycero-3-phosphocholine), DMPG (1,2-dimyristoyl-sn-glycero-3-phospho-(10-rac-glycerol)), and DMPE-PEG (1,2-dimyristoyl-sn-glycero-3-phosphoethanolamine-*N*-[methoxy(polyethylene glycol)-2000]) (from Avanti Polar Lipids) were used in the molar ratio 9:7.5:2.5. The lipids were first dissolved in a 1:3 methanol:chloroform solution, and then the organic solvents were removed completely under vacuum using a Heidolph rotary evaporator with a Vacuubrand vacuum pump. The resulting lipid film was hydrated with 50 mM Tris buffer, pH 7.4, for at least one hour at 35 °C. After sonication for 10 min, the lipid dispersions were extruded through a 100 nm pore diameter polycarbonate filter (21 times) using an Avanti mini-extruder fitted with two 1 mL airtight syringes. Immediately before the SAXS experiment, a peptide solution with the adequate concentration for the target lipid:peptide ratio was mixed 1:1 with the lipid solution (1:1) using a micropipette.

SAXS experiments of the peptide-lipid mixes were performed at the automated BM29 bioSAXS beamline at the European Synchrotron Radiation Facility (ESRF) in Grenoble, France [35]. The data was obtained using an energy of 12.5 keV and a detector distance of 2.87 m, covering a q range of about 0.0047 Å^−1^ to 0.5 Å^−1^. The data set was calibrated to an absolute intensity scale using water as a primary standard. 40 µL samples were run through a capillary using the flow mode of the automated sample changer [36]. SAXS data was collected in ten successive frames of 0.5 s each to monitor radiation damage and the data reduction was done using the standard tool at BM29 [37].

The SAXS results of the lipid-peptide mixes were analysed using the theoretical model described in detail in [29]. In short, the model provides a detailed description of the membrane by dividing into probability functions for each component (lipid sub-units/peptide) across the bilayer.

## 4. Conclusions

We presented a structure-activity relationship study of the small antimicrobial all-d octapeptide amide *H*-kk(1-nal)fk(1-nal)k(nle)-NH2 **(D2D)** based on 36 analogues, which reveals several interesting results. d-alanine-scanning of D2D revealed that the hydrophobic residues in position 3 (1-nal), 4 (phe), 6 (1-nal) and 8 (nle) are important for antimicrobial activity. Furthermore, lysine could be replaced by alanine in either position 1, 2, 5 or 7 without losing antimicrobial activity. Varying hydrophobicity at position 5 (lys) or 7 (lys) didn’t lead to overall more potent and less toxic compounds. Exchanging 1-nal^6^ with lys^7^ and lys^7^ with nle^8^ led to loss of activity. The N- and C-terminus truncations indicated that the full octapeptide is required for as potent activity as **D2D**, however [des k^1^]-**D2D** retained some antibacterial activity. Substitutions with arginine did not lead to overall more potent and less toxic compounds.

Our best candidate **5**, showed MICs of 4 µg/mL against *A. baumannii*, *E. coli*, *P. aeruginosa* and *S. aureus* with a hemolytic activity of 47 % against red blood cells. Time-kill kinetics showed that compound **5** kill bacteria in a concentration-dependent manner. The peptide showed a > 3-log reduction in CFU/mL except for *P. aeruginosa*.

Analysis of CD spectra of **D2D** and compounds **1**–**8** were complicated by spectral contributions from naphthylalanine in position 3 and 6. The far-UV band shape could be consistent with α-helical secondary structure for **D2D** and **1**–**8** in a trifluoroethanol-containing aqueous solvent.

Small angle X-ray scattering (SAXS) experiments showed that **D2D** and compound **1**, **3**, **4**, **6** and **8** can be described as random unstructured polymer-like chains model. Compound **5** can be described with a nanotube structure model. Compound **2** can be described both with a nanotube structure and a filament-like structure model, while compound **7** only can be explained by larger filament-like structures.

Lipid interaction probed by SAXS showed that a higher amount of compound **5** (~50–60%) inserts into the bilayer compared to **D2D** (~30–50%).

**D2D** still remains the lead compound, however compound **5** is also an interesting antimicrobial peptide that needs further investigation mainly due to its formation of nanotube structures and minor increase in antimicrobial activity compared to **D2D**.

## Figures and Tables

**Table 1:** Sequence, MIC and hemolytic activity of 36 analogues of **D2D.**

		Minimum Inhibitory Concentration ^[a]^				
No.	Sequence ^[b]^	E.C. ^[c]^	S.A. ^[d]^	P.A. ^[e]^	A.B. ^[f]^	%H ^[g]^
**D2D**	kk(1-nal)fk(1-nal)k(nle)-NH_2_	8	8	4	4	23
**1**	ak(1-nal)fk(1-nal)k(nle)-NH_2_	8	2	8	4	41
**2**	ka(1-nal)fk(1-nal)k(nle)-NH_2_	16	8	16	16	59
**3**	kkafk(1-nal)k(nle)-NH_2_	>128	64	16	16	2
**4**	kk(1-nal)ak(1-nal)k(nle)-NH_2_	>128	128	32	64	22
**5**	kk(1-nal)fa(1-nal)k(nle)-NH_2_	4	4	4	4	47
**6**	kk(1-nal)fkak(nle)-NH_2_	>128	>128	>128	>128	10
**7**	kk(1-nal)fk(1-nal)a(nle)-NH_2_	8	8	8	4	67
**8**	kk(1-nal)fk(1-nal)ka-NH_2_	64	64	16	64	5
**9**	kk(1-nal)f(1-nal)(1-nal)k(nle)-NH_2_	64	4	128	32	20
**10**	kk(1-nal)f(dap)(1-nal)k(nle)-NH_2_	8	16	16	16	28
**11**	kk(1-nal)f(nle)(1-nal)k(nle)-NH_2_	16	4	64	64	33
**12**	kk(1-nal)ff(1-nal)k(nle)-NH_2_	16	4	32	16	74
**13**	kk(1-nal)fs(1-nal)k(nle)-NH_2_	4	8	32	8	29
**14**	kk(1-nal)ft(1-nal)k(nle)-NH_2_	8	8	32	8	28
**15**	kk(1-nal)fy(1-nal)k(nle)-NH_2_	8	2	16	8	40
**16**	kk(1-nal)fv(1-nal)k(nle)-NH_2_	32	4	64	64	54
**17**	kk(1-nal)fk(1-nal)(1-nal)(nle)-NH_2_	16	2	32	4	89
**18**	kk(1-nal)fk(1-nal)(dap)(nle)-NH_2_	8	16	16	16	21
**19**	kk(1-nal)fk(1-nal)(nle)(nle)-NH_2_	8	4	32	8	56
**20**	kk(1-nal)fk(1-nal)f(nle)-NH_2_	8	4	32	4	91
**21**	kk(1-nal)fk(1-nal)s(nle)-NH_2_	8	16	64	16	85
**22**	kk(1-nal)fk(1-nal)t(nle)-NH_2_	8	8	32	8	66
**23**	kk(1-nal)fk(1-nal)y(nle)-NH_2_	8	4	64	8	74
**24**	kk(1-nal)fk(1-nal)v(nle)-NH_2_	8	4	32	8	50
**25**	kk(1-nal)fkk(1-nal)(nle)-NH_2_	128	64	32	32	50
**26**	kk(1-nal)fk(1-nal)(nle)k-NH_2_	128	64	32	32	18
**27**	k(1-nal)fk(1-nal)k(nle)-NH_2_	8	8	32	16	49
**28**	(1-nal)fk(1-nal)k(nle)-NH_2_	>128	>128	>128	>128	62
**29**	fk(1-nal)k(nle)-NH_2_	>128	>128	128	>128	12
**30**	kk(1-nal)fk(1-nal)k-NH_2_	>128	>128	128	>128	11
**31**	kk(1-nal)fk(1-nal)-NH_2_	>128	>128	128	>128	20
**32**	kk(1-nal)fk-NH_2_	>128	>128	>128	>128	5
**33**	rk(1-nal)fk(1-nal)k(nle)-NH_2_	8	8	16	16	60
**34**	kr(1-nal)fk(1-nal)k(nle)-NH_2_	8	4	16	16	35
**35**	kk(1-nal)fr(1-nal)k(nle)-NH_2_	8	4	16	16	49
**36**	kk(1-nal)fk(1-nal)r(nle)-NH_2_	8	2	16	8	51

[a]: Minimum Inhibitory Concentration (MICs) were determined in triplicate by the broth microdilution method in accordance with CLSI standards. MICs are reported in µg/mL. To convert to µM use µM = ((µg/mL)/M_w_)·1000; [b]: Compounds tested in this study. All D-peptides were synthesized as *C*-terminal amides. Changes relative to **D2D** are highlighted. Unusual amino acids in the sequences: 1-naphthylalanine (1-nal) and norleucine (nle) [c]: *E. coli* (ATCC 25922); [d] *S. aureus* (ATCC 29213); [e]: *P. aeruginosa* (ATCC 27853); [f]: *A. baumannii* (ATCC 19606); [g] % Hemolysis at 150 µM.

## Supplementary Materials

The following are available online, Peptide structures, Analytical HPLC chromatograms, MALDI-TOF-MS spectra. Figure S1: SAXS data for peptide **5**; Figure S2: SAXS data for peptide **2**; Figure S3: SAXS data for peptide **1**, **3**, **4**, **6** and **8**. Table S1: Peptide mass, HPLC retention time and purity; Table S2: Important fit parameters from the analysis of liposomes-peptide mixes

## References

[1] Konaklieva M.I. (2018). Addressing Antimicrobial Resistance through New Medicinal and Synthetic Chemistry Strategies. SLAS DISCOVERY: Adv. Life Sci. R D.

[2] Tacconelli E. (2017). Global priority list of antibiotic-resistant bacteria to guide research, discovery, and development of new antibiotics. World Health Organ..

[3] Mulani M.S., Kamble E.E., Kumkar S.N., Tawre M.S., Pardesi K.R. (2019). Emerging Strategies to Combat ESKAPE Pathogens in the Era of Antimicrobial Resistance: A Review. Front. Microbiol..

[4] Butler M.S., Blaskovich M.A.T., Cooper M.A. (2017). Antibiotics in the clinical pipeline at the end of 2015. J Antibiot..

[5] Mercer D.K., O’Neil D.A. (2013). Peptides as the next generation of anti-infectives. Future Med. Chem..

[6] Zasloff M. (2002). Antimicrobial peptides of multicellular organisms. Nature.

[7] Greber K.E., Dawgul M. (2017). Antimicrobial Peptides Under Clinical Trials. Curr. Top. Med. Chem..

[8] Haney E.F., Mansour S.C., Hancock R.E.W., Hansen P.R. (2017). Antimicrobial Peptides: An Introduction. Antimicrobial Peptides: Methods and Protocols.

[9] Fosgerau K., Hoffmann T. (2015). Peptide therapeutics: Current status and future directions. Drug Discov. Today.

[10] Joo S.H. (2012). Cyclic Peptides as Therapeutic Agents and Biochemical Tools. Biomol. Ther..

[11] Molchanova N., Hansen P.R., Franzyk H. (2017). Advances in Development of Antimicrobial Peptidomimetics as Potential Drugs. Molecules.

[12] Bessalle R., Kapitkovsky A., Gorea A., Shalit I., Fridkin M. (1990). All-D-magainin-chirality, antimicrobial activity and proteolytic resistance. FEBS Lett..

[13] Zuckermann R.N., Kerr J.M., Kent S.B.H., Moos W.H. (1992). Efficient method for the preparation of peptoids (oligo(N-substituted glycines)) by submonomer solid-phase synthesis. J. Am. Chem. Soc..

[14] Schmitt M.A., Weisblum B., Gellman S.H. (2006). Interplay among Folding, Sequence, and Lipophilicity in the Antibacterial and Hemolytic Activities of α/β-Peptides. J. Am. Chem. Soc..

[15] Ryge T.S., Frimodt-Moller N., Hansen P.R. (2008). Antimicrobial activities of twenty lysine-peptoid hybrids against clinically relevant bacteria and fungi. Chemotherapy.

[16] Molchanova N., Hansen P.R., Damborg P., Nielsen H.M., Franzyk H. (2017). Lysine-Based α-peptide/β-peptoid peptidomimetics: Influence of hydrophobicity, fluorination and distribution of cationic charge on antimicrobial activity and cytotoxicity. ChemMedChem.

[17] Niu Y., Wu H., Li Y., Hu Y., Padhee S., Li Q., Cao C., Cai J. (2013). AApeptides as a new class of antimicrobial agents. Org. Biomol. Chem..

[18] Greco I., Hansen J.E., Jana B., Molchanova N., Oddo A., Thulstrup P.W., Damborg P., Guardabassi L., Hansen P.R. (2019). Structure–Activity Study, Characterization, and Mechanism of Action of an Antimicrobial Peptoid D2 and Its D- and L-Peptide Analogues. Molecules.

[19] Greco I., Hummel B., Vasir J., Watts J., Koch J., Hansen J., Nielsen H., Damborg P., Hansen P. (2018). In Vitro ADME Properties of Two Novel Antimicrobial Peptoid-Based Compounds as Potential Agents against Canine Pyoderma. Molecules.

[20] Staubitz P., Peschel A., Nieuwenhuizen W.F., Otto M., Götz F., Jung G., Jack R.W. (2001). Structure-function relationships in the tryptophan-rich, antimicrobial peptide indolicidin. J. Pept. Sci..

[21] Grieco P., Luca V., Auriemma L., Carotenuto A., Saviello M.R., Campiglia P., Barra D., Novellino E., Mangoni M.L. (2011). Alanine scanning analysis and structure–function relationships of the frog-skin antimicrobial peptide temporin-1Ta. J. Pept. Sci..

[22] Ifrah D., Doisy X., Ryge T., Hansen P. (2005). Structure-activity relationship study of anoplin. J. Pept. Sci..

[23] Manabe T., Kawasaki K. (2017). D-form KLKLLLLLKLK-NH2 peptide exerts higher antimicrobial properties than its L-form counterpart via an association with bacterial cell wall components. Sci. Rep..

[24] Oddo A., Thomsen T.T., Kjelstrup S., Gorey C., Franzyk H., Frimodt-Møller N., Løbner-Olesen A., Hansen P.R. (2016). An all-D amphipathic undecapeptide shows promising activity against colistin-resistant strains of Acinetobacter baumannii and a dual mode of action. Antimicrob. Agents Chemother..

[25] Schiffer M., Edmunson A.B. (1967). Use of helical wheels to represent the structures of proteins and to identify segments with helical potential. Biophys. J..

[26] Nielsen S.L., Frimodt-Moller N., Kragelund B.B., Hansen P.R. (2007). Structure activity study of the antibacterial peptide fallaxin. Protein Sci..

[27] Deslouches B., Hasek M.L., Craigo J.K., Steckbeck J.D., Montelaro R.C. (2016). Comparative functional properties of engineered cationic antimicrobial peptides consisting exclusively of tryptophan and either lysine or arginine. J. Med. Microbiol..

[28] Greenfield N.J. (2006). Using circular dichroism spectra to estimate protein secondary structure. Nat. Protoc..

[29] Nielsen J.E., Bjørnestad V.A., Lund R. (2018). Resolving the structural interactions between antimicrobial peptides and lipid membranes using small-angle scattering methods: The case of indolicidin. Soft Matter.

[30] Nielsen J.E., Lind T.K., Lone A., Gerelli Y., Hansen P.R., Jenssen H., Cárdenas M., Lund R. (2019). A biophysical study of the interactions between the antimicrobial peptide indolicidin and lipid model systems. BBA Biomembr..

[31] Greco I., Emborg A.P., Jana B., Molchanova N., Oddo A., Damborg P., Guardabassi L., Hansen P.R. (2019). Characterization, mechanism of action and optimization of activity of a novel peptide-peptoid hybrid against bacterial pathogens involved in canine skin infections. Sci. Rep.-UK.

[32] Wiegand I., Hilpert K., Hancock R.E.W. (2008). Agar and broth dilution methods to determine the minimal inhibitory concentration (MIC) of antimicrobial substances. Nat. Protoc..

[33] Oddo A., Hansen P.R. (2017). Hemolytic Activity of Antimicrobial Peptides. Methods Mol. Biol..

[34] Sun J., Jiang X., Lund R., Downing K.H., Balsara N.P., Zuckermann R.N. (2016). Self-assembly of crystalline nanotubes from monodisperse amphiphilic diblock copolypeptoid tiles. Proc. Natl. Acad. Sci. USA.

[35] Pernot P., Round A., Barrett R., De Maria Antolinos A., Gobbo A., Gordon E., Huet J., Kieffer J., Lentini M., Mattenet M. (2013). Upgraded ESRF BM29 beamline for SAXS on macromolecules in solution. J. Synchrotron Radiat..

[36] Round A., Felisaz F., Fodinger L., Gobbo A., Huet J., Villard C., Blanchet C.E., Pernot P., McSweeney S., Roessle M. (2015). BioSAXS Sample Changer: A robotic sample changer for rapid and reliable high-throughput X-ray solution scattering experiments. Acta. Crystallogr. D.

[37] De Maria Antolinos A., Pernot P., Brennich M.E., Kieffer J., Bowler M.W., Delageniere S., Ohlsson S., Malbet Monaco S., Ashton A., Franke D. (2015). ISPyB for BioSAXS, the gateway to user autonomy in solution scattering experiments. Acta. Crystallogr. D.

