# Supplementary material for: Structure-Activity Study of an All-d Antimicrobial Octapeptide D2D
Source: Molecules. 2019 Dec 13;24(24):4571. doi: 10.3390/molecules24244571 (PMC6943423; doi:10.3390/molecules24244571)
Supplement: Supplementary file 1 [file molecules-24-04571-s001.pdf]

Supplemental Materials

Structure-activity study of an all-D antimicrobial octapeptide D2D

Abdullah Lone [1,2], Thomas T. Thomsen [3,4], Josefine Eilsø Nielsen[5], Peter W. Thulstrup[6], Rasmus, N. Klitgaard[4], Anders Løbner-Olesen[4], Reidar Lund[5], Håvard Jenssen[2] and Paul R. Hansen [1*]

1 Department of Drug Design and Pharmacology, Faculty of Health and Medical Sciences, University of Copenhagen, Universitetsparken 2, 2100 Copenhagen, Denmark; alone@ruc.dk; prh@sund.ku.dk
2 Present Address: Department of Science and Environment, Roskilde University, 4000 Roskilde, Denmark; jenssen@ruc.dk
3 Department of Clinical Microbiology, Rigshospitalet, Henrik Harpestrengs Vej 4A, 2100 Copenhagen. Denmark; thomas.thomsen@bio.ku.dk
4 Department of Biology, Section for functional Genomics, University of Copenhagen, Ole Maaløes Vej 5, 2200 Copenhagen, Denmark; rasmusklitgaard49@gmail.com; lobner@bio.ku.dk
5 Department of Chemistry, University of Oslo, Sem Sælands vei 26, 0371 Oslo, Norway; j.e.nielsen@kjemi.uio.no; reidar.lund@kjemi.uio.no
6 Department of Chemistry, University of Copenhagen, Universitetsparken 5, 2100 Copenhagen, Denmark; pwt@chem.ku.dk

* Correspondence: prh@sund.ku.dk; Tel: +4535336625

Received: date; Accepted: date; Published: date

Content:

Page 2: Table S1: Peptide mass, HPLC retention time and purity

Page 3-15: Peptide structures

Page 16-34: Analytical HPLC chromatograms

Page 35-53: MALDI-TOF-MS spectra

Page 54: Figure S1 SAXS data for peptide 5

Page 55: Figure S2: SAXS data for peptide 2

Page 56: Figure S3: SAXS data for peptide 1, 3, 4, 6 and 8

Page 57: Table S2: Important fit parameters from the analysis of liposomes-peptide mixes

Table S1: Peptide mass, retention time and purity

Peptide	Molecular weight	MALDI mass (MH$^+$)	Retention time (min)	Purity
**D2-D**	1184.54	1185.77	15.45	100 %
**1**	1127.45	1128.54	16.48	96 %
**2**	1127.45	1128.56	16.48	98 %
**3**	1058.38	1059.49	14.45	96 %
**4**	1108.44	1109.54	14.72	99 %
**5**	1127.45	1128.62	16.49	98 %
**6**	1058.38	1059.76	13.39	100 %
**7**	1127.45	1128.75	16.45	99 %
**8**	1142.46	1143.64	14.47	98 %
**9**	1253.60	1253.92	18.24	98 %
**10**	1142.46	1143.48	16.00	98 %
**11**	1169.53	1170.46	17.45	98 %
**12**	1203.54	1204.46	17.43	98 %
**13**	1143.45	1144.48	17.90	96 %
**14**	1157.47	1158.44	18.03	96 %
**15**	1219.54	1220.76	17.11	95 %
**16**	1155.50	1156.51	17.31	97 %
**17**	1253.60	1255.13	18.45	98 %
**18**	1142.46	1143.97	15.63	95 %
**19**	1169.53	1170.93	17.41	97 %
**20**	1203.54	1204.75	17.50	96 %
**21**	1143.45	1145.08	16.34	96 %
**22**	1157.47	1158.80	16.44	96 %
**23**	1219.54	1220.84	16.66	93 %
**24**	1155.50	1156.79	16.84	95 %
**25**	1184.54	1186.18	15.48	98 %
**26**	1184.54	1186.04	15.46	99 %
**27**	1056.37	1057.97	16.42	97 %
**28**	928.19	929.63	17.22	97 %
**29**	730.96	732.30	15.56	99 %
**30**	1071.38	1072.89	14.47	94 %
**31**	943.21	944.60	15.59	96 %
**32**	745.97	747.30	13.05	98 %
**33**	1212.56	1213.95	15.72	97 %
**34**	1212.56	1213.98	15.77	98 %
**35**	1212.56	1214.06	15.71	97 %
**36**	1212.56	1214.13	15.73	97 %

Peptide structures

D2D

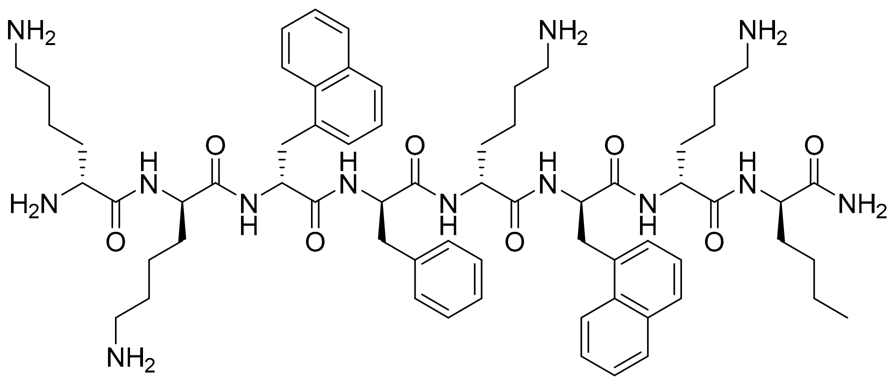

Molecular Weight: 1184,54

1

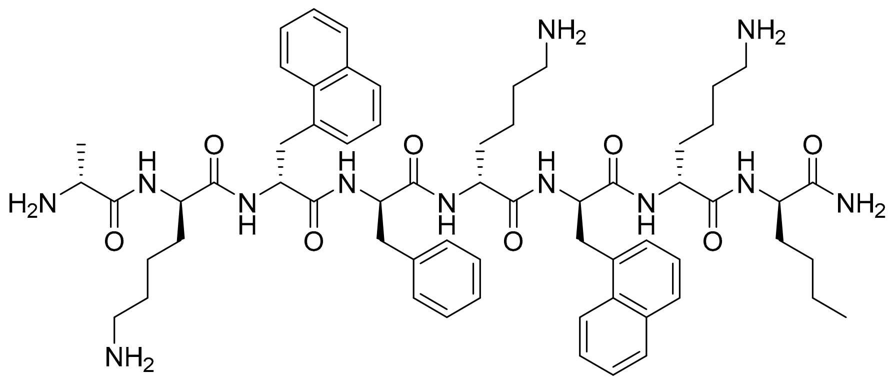

Molecular Weight: 1127,45

2

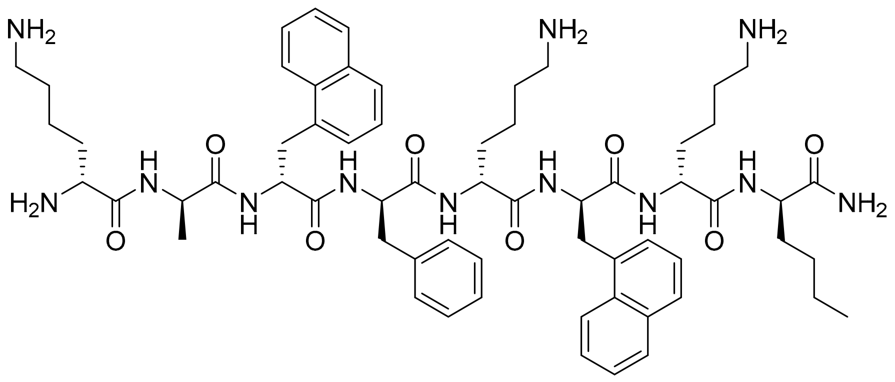

Molecular Weight: 1127,45

3

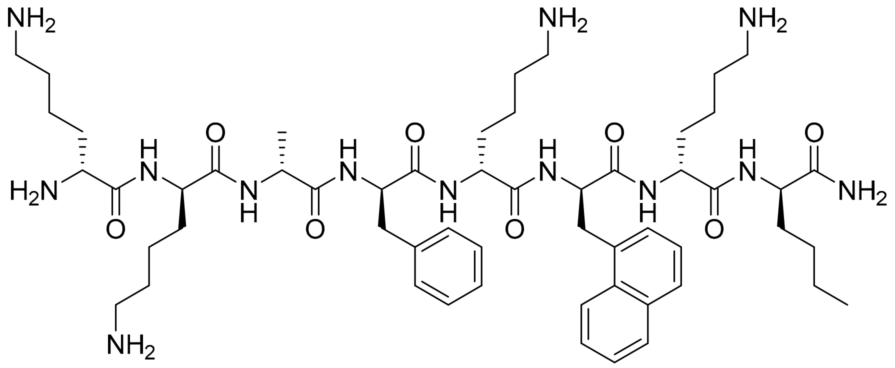

Molecular Weight: 1058,38

4

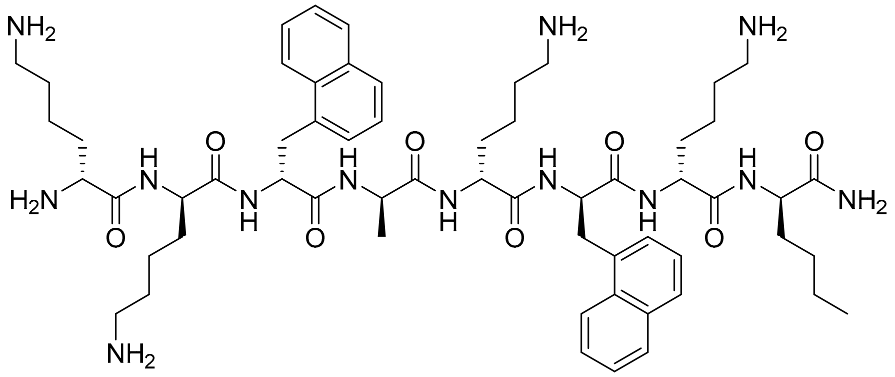

Molecular Weight: 1108,44

5

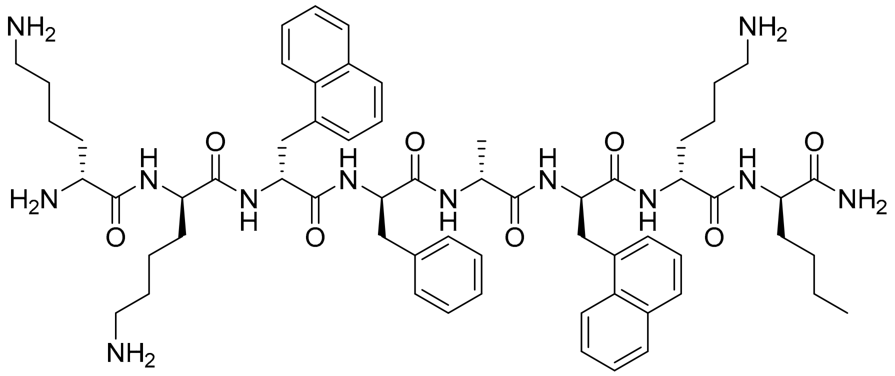

Molecular Weight: 1127,45

6

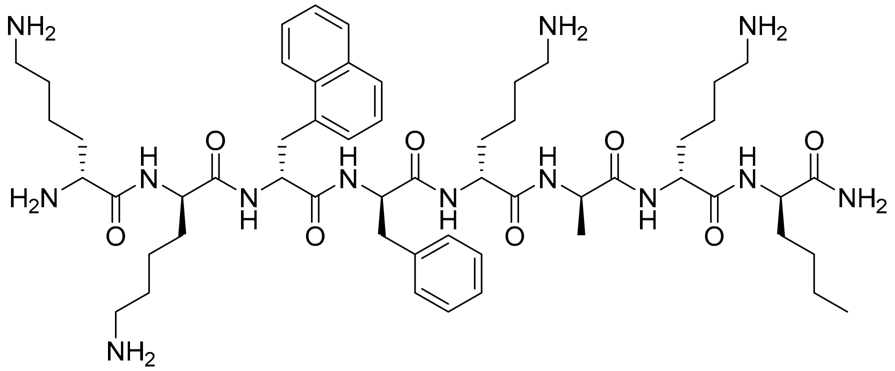

Molecular Weight: 1058,38

7

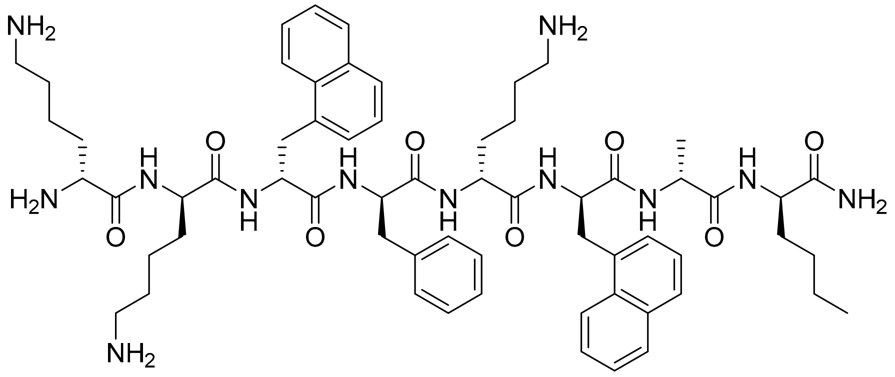

Molecular Weight: 1127,45

8

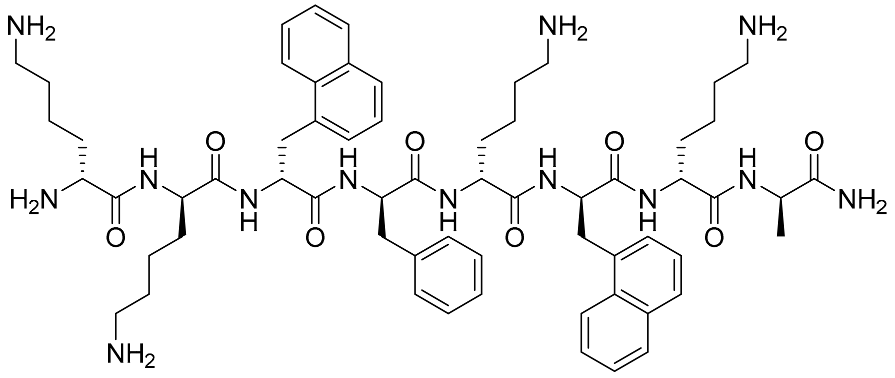

Molecular Weight: 1142,46

9

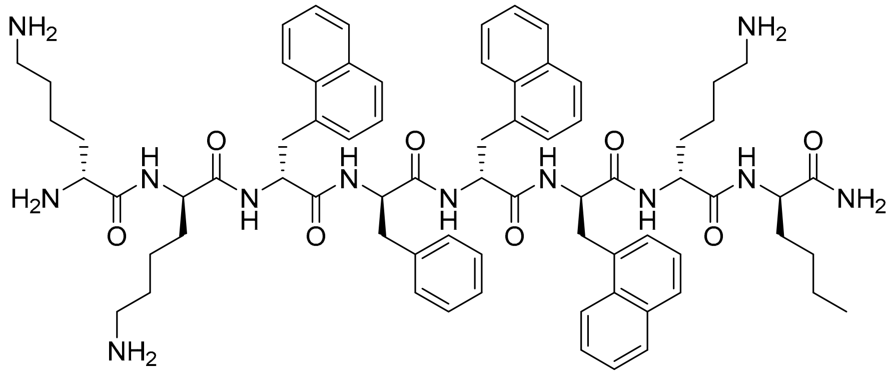

Molecular Weight: 1253,60

10

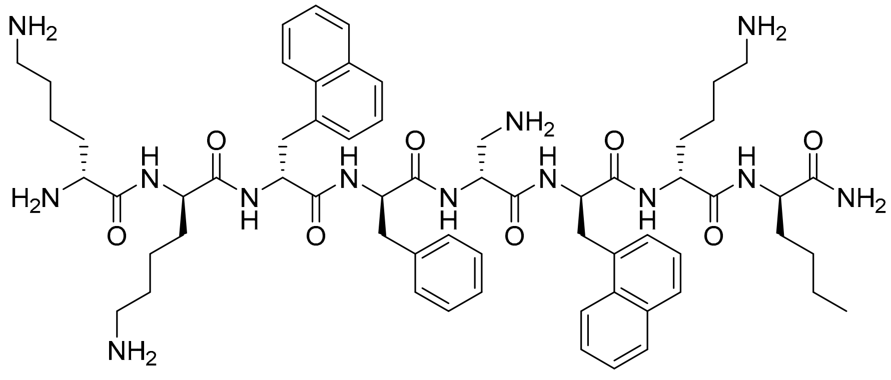

Molecular Weight: 1142,46

11

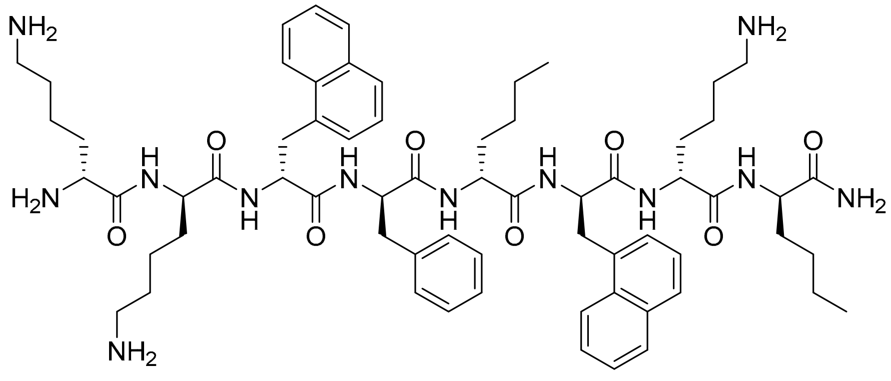

Molecular Weight: 1169,53

12

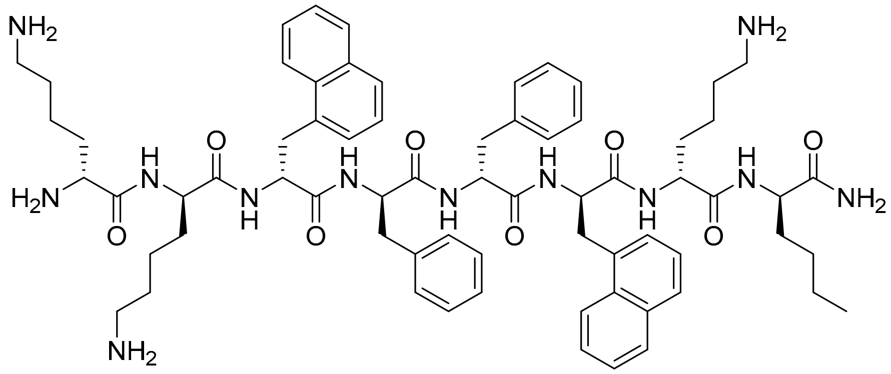

Molecular Weight: 1203,54

13

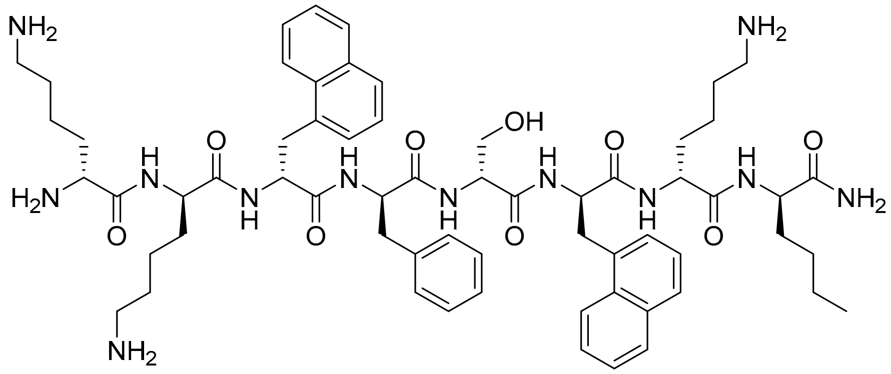

Molecular Weight: 1143,45

14

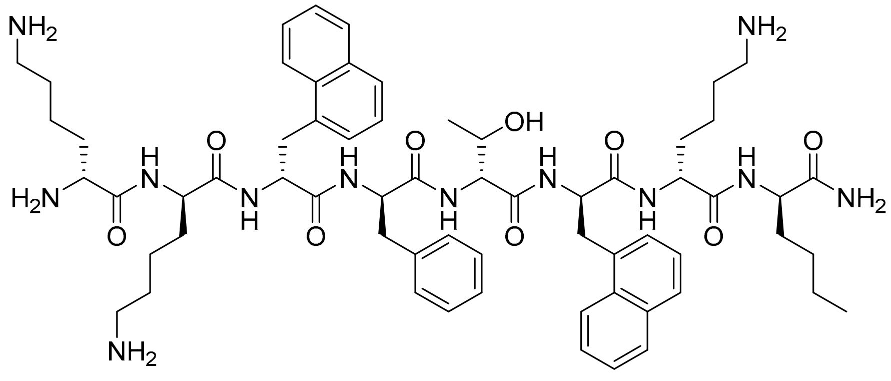

Molecular Weight: 1157,47

15

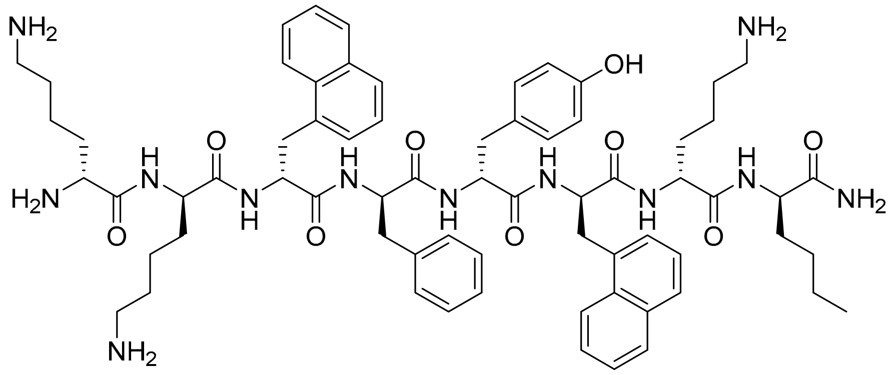

Molecular Weight: 1219,54

16

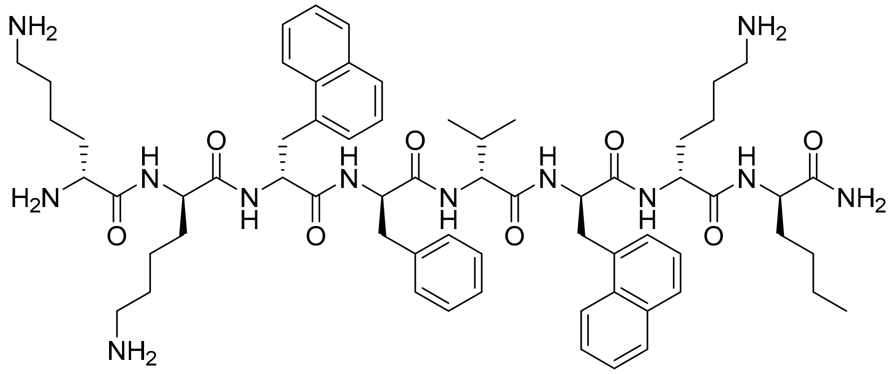

Molecular Weight: 1155,50

17

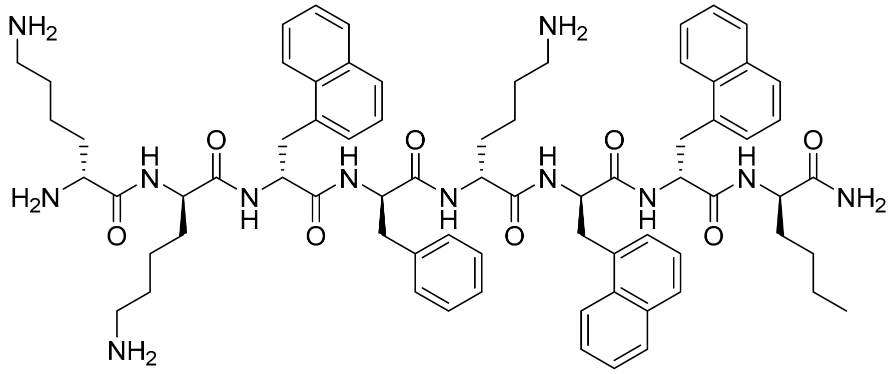

Molecular Weight: 1253,60

18

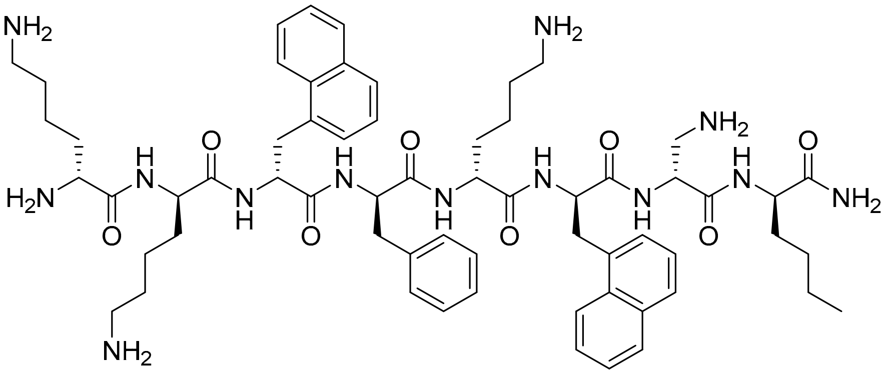

Molecular Weight: 1142,46

19

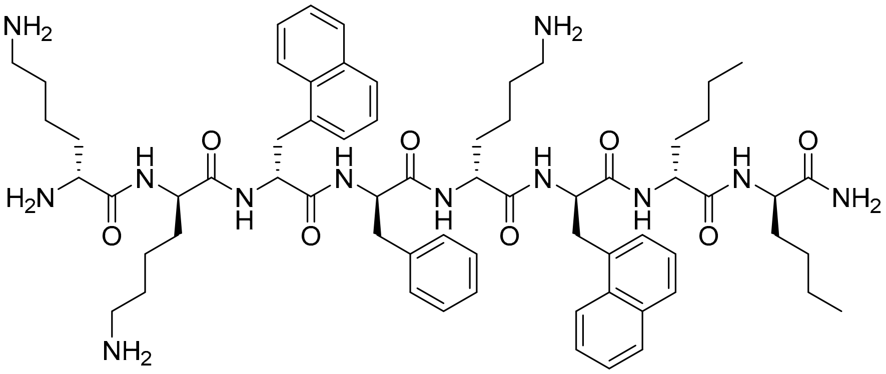

Molecular Weight: 1169,53

20

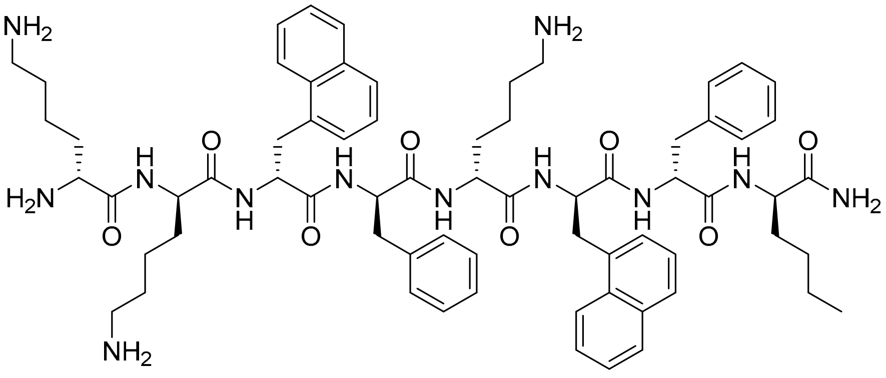

Molecular Weight: 1203,54

21

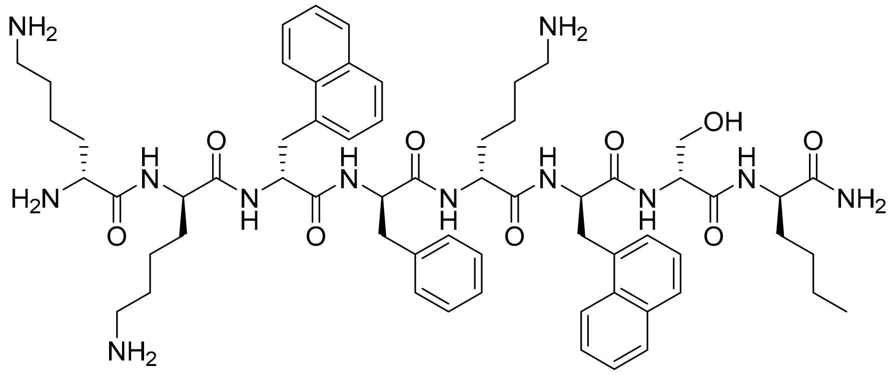

Molecular Weight: 1143,45

22

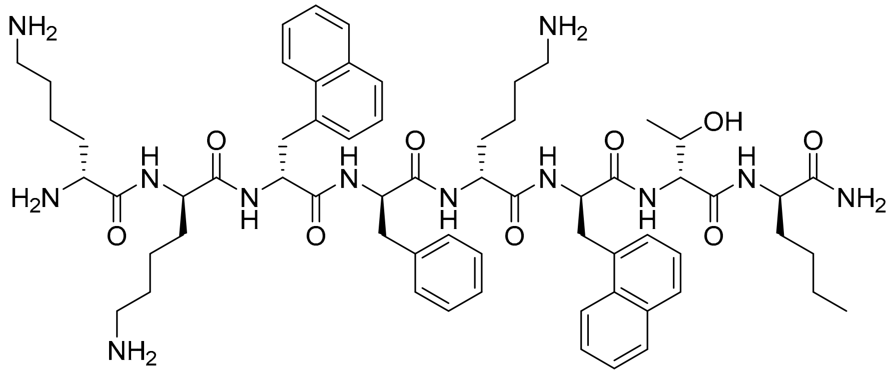

Molecular Weight: 1157,47

23

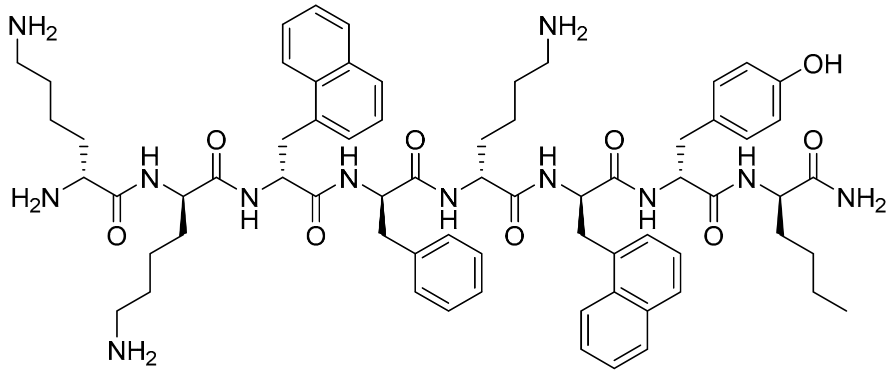

Molecular Weight: 1219,54

24

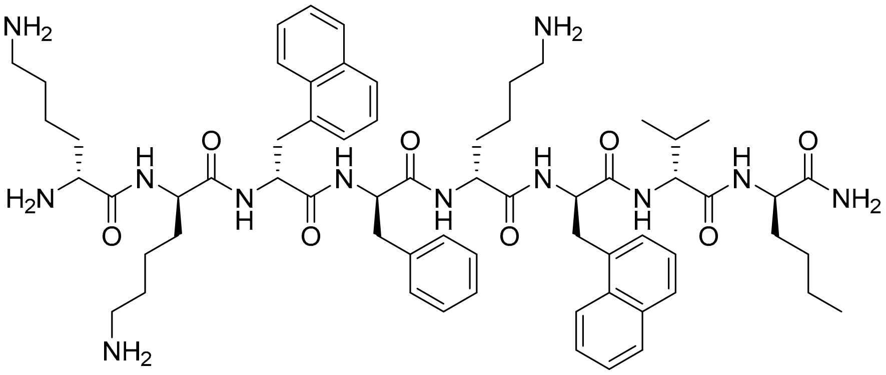

Molecular Weight: 1155,50

25

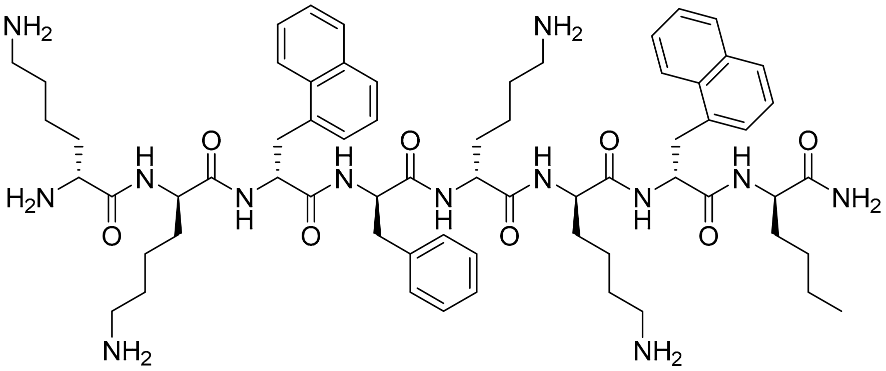

Molecular Weight: 1184,54

26

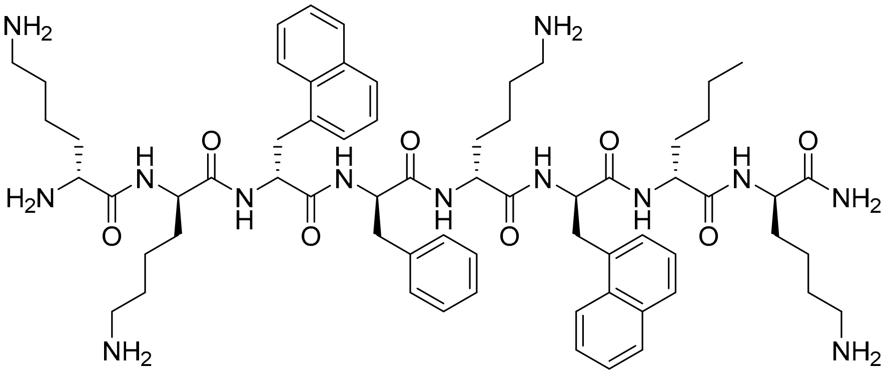

Molecular Weight: 1184,54

27

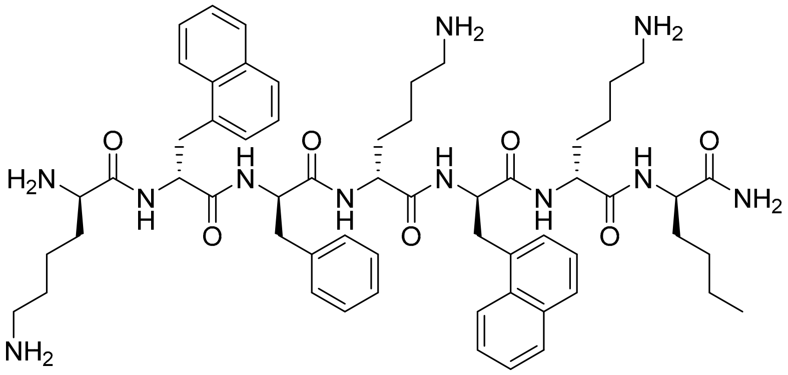

Molecular Weight: 1056,37

28

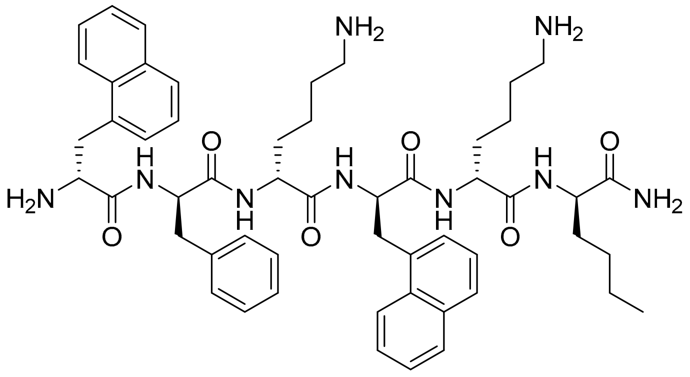

Molecular Weight: 928,19

29

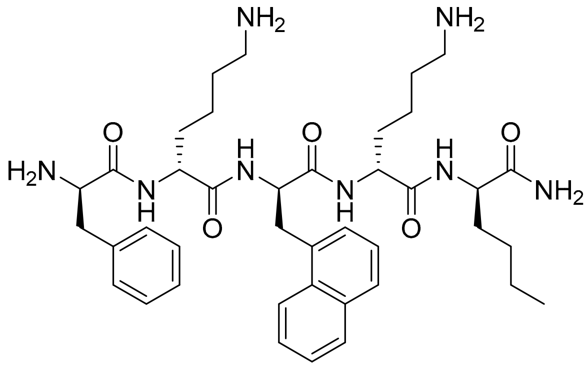

Molecular Weight: 730,96

30

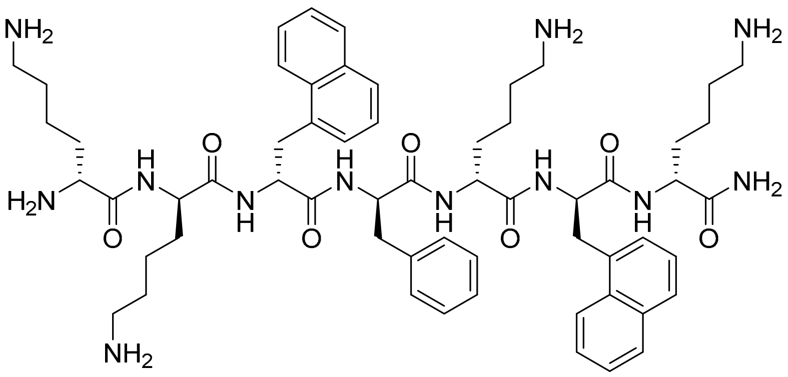

Molecular Weight: 1071,38

31

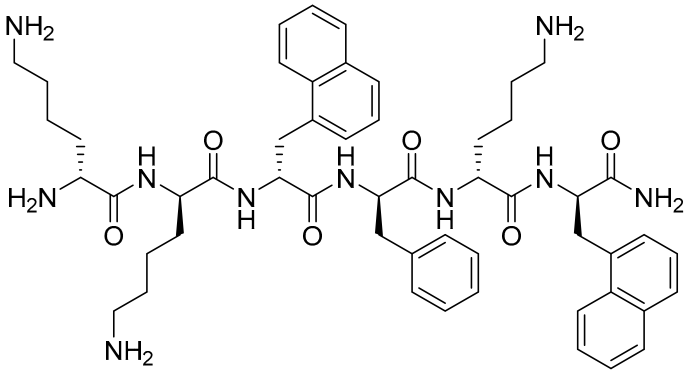

Molecular Weight: 943,21

32

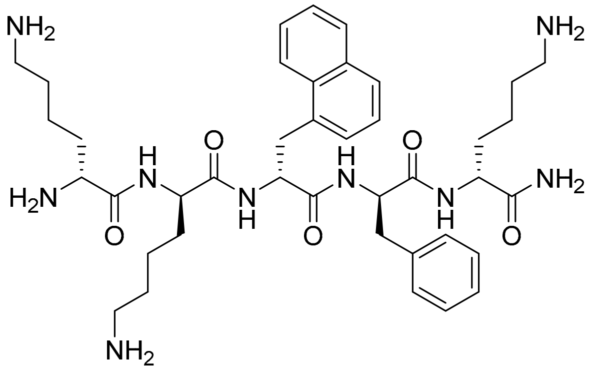

Molecular Weight: 745,97

33

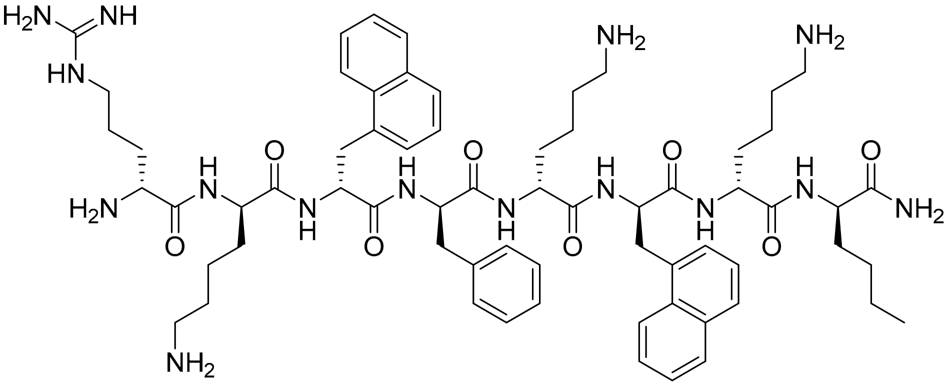

Molecular Weight: 1212,56

34

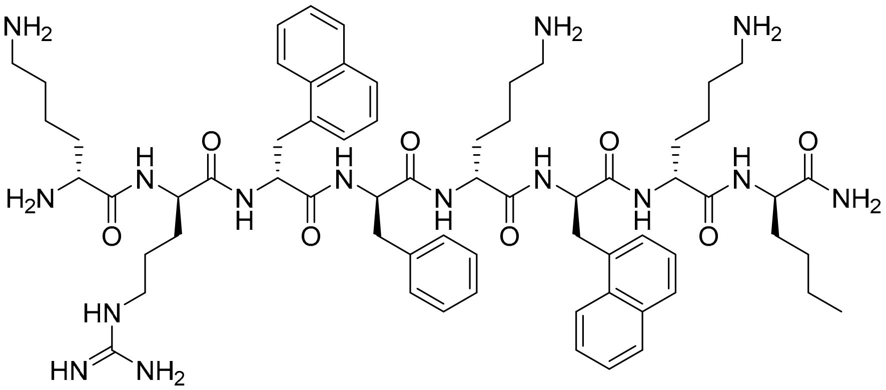

Molecular Weight: 1212,56

35

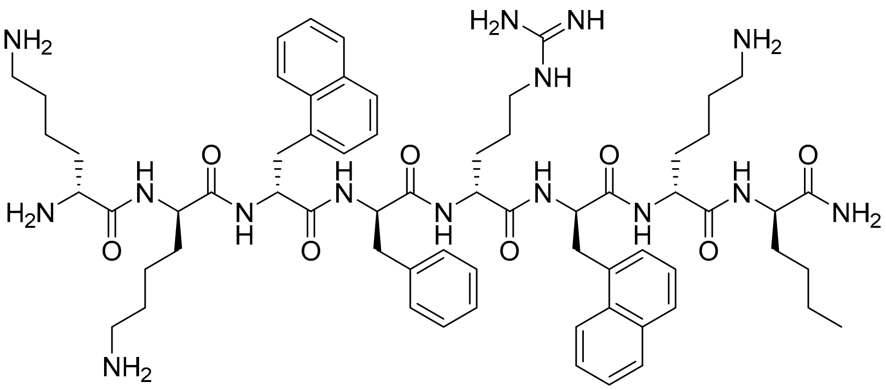

Molecular Weight: 1212,56

36

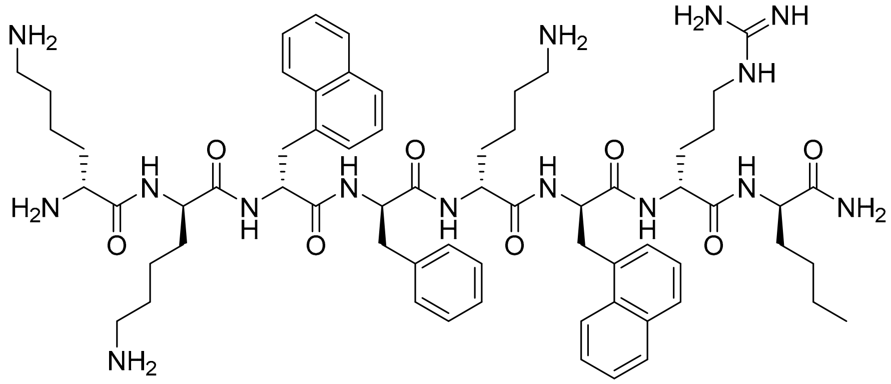

Molecular Weight: 1212,56

Analytical chromatograms

D2-D
Retention time: 15.45 min
Purity: 100 %

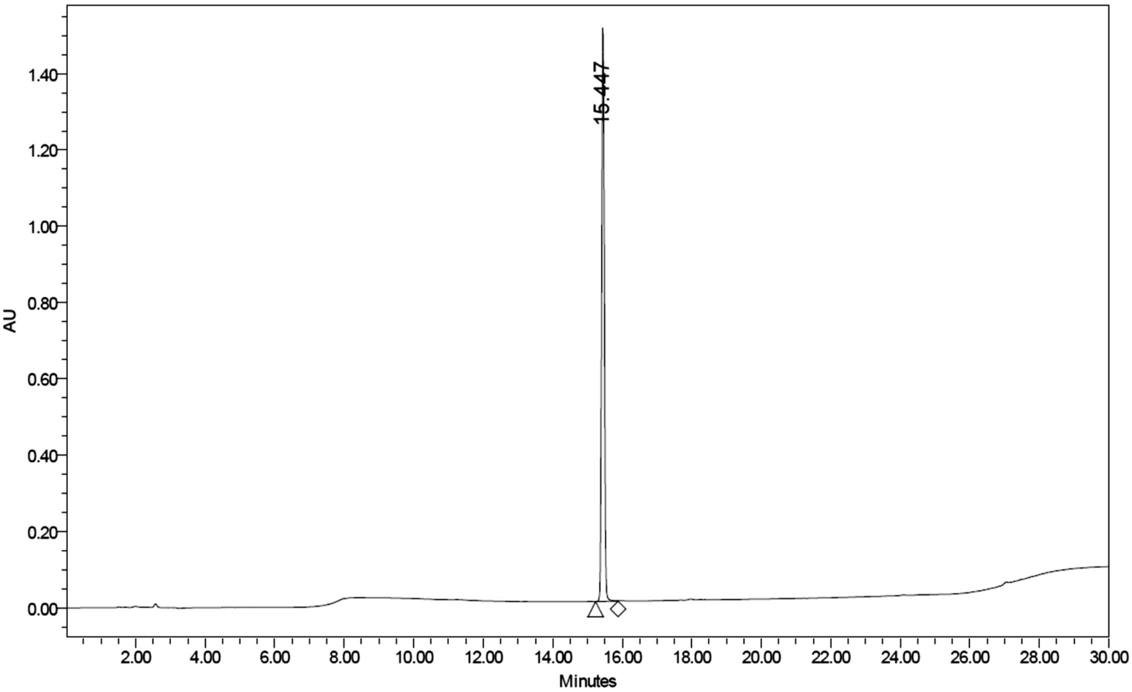

1
Retention time: 16.48 min
Purity: 96 %

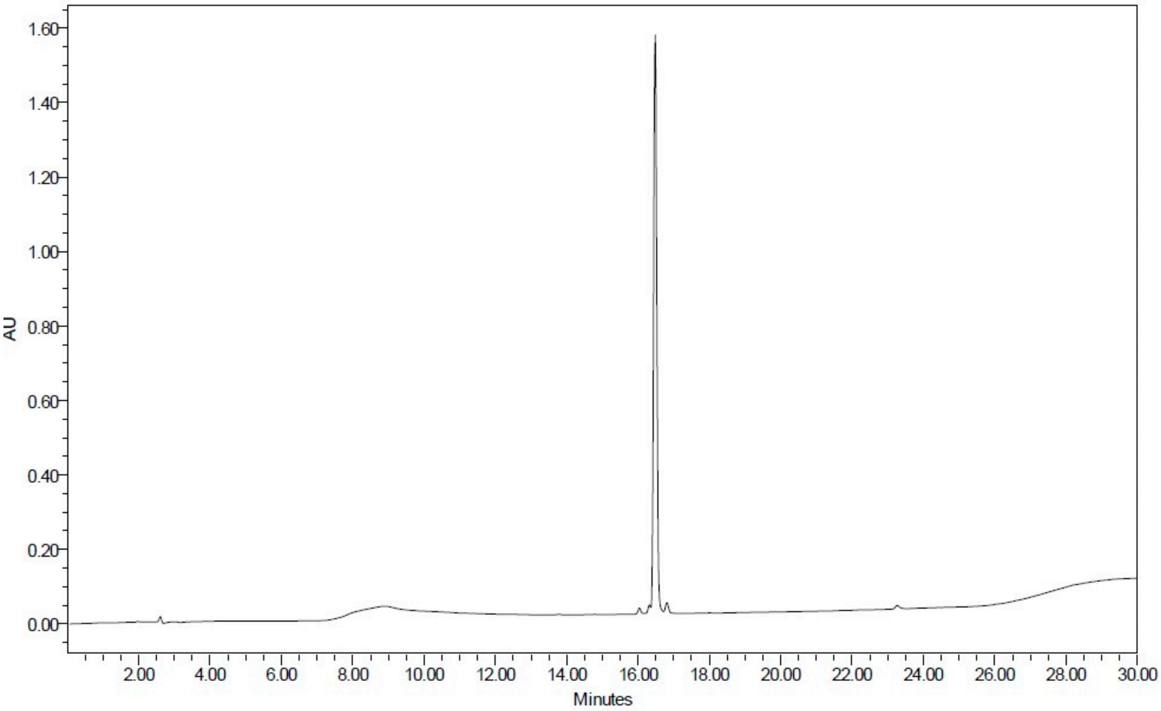

2

Retention time: 16.48 min

Purity: 98 %

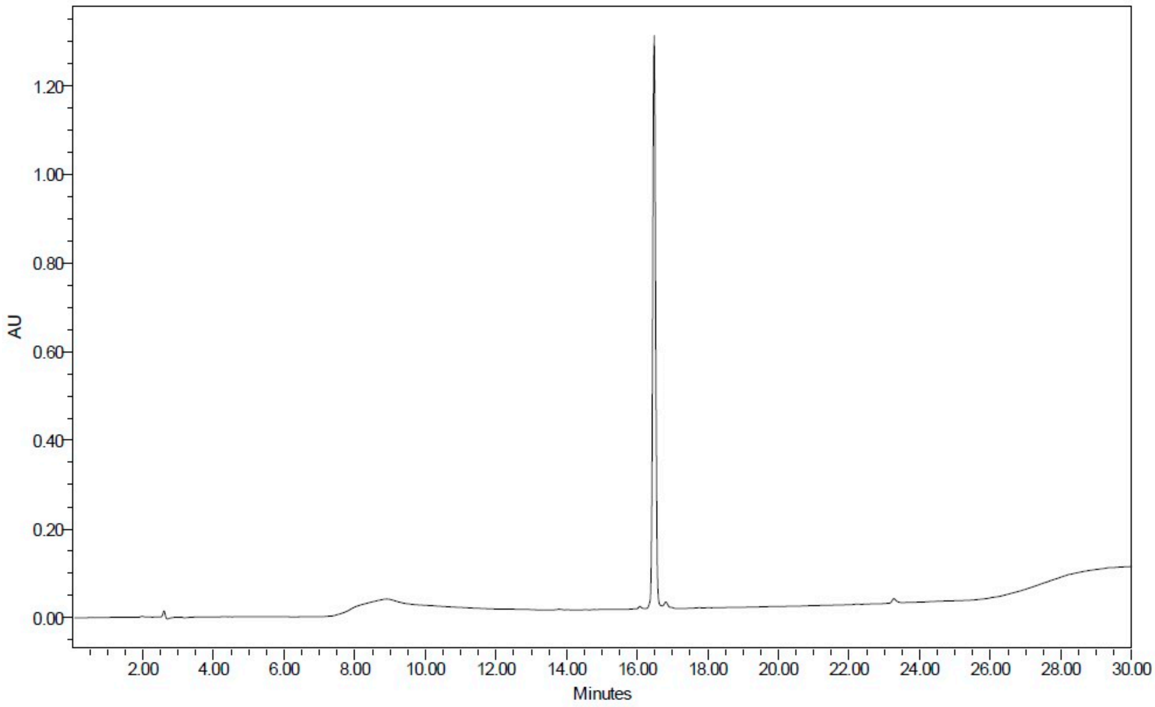

3

Retention time: 14.45 min

Purity: 96 %

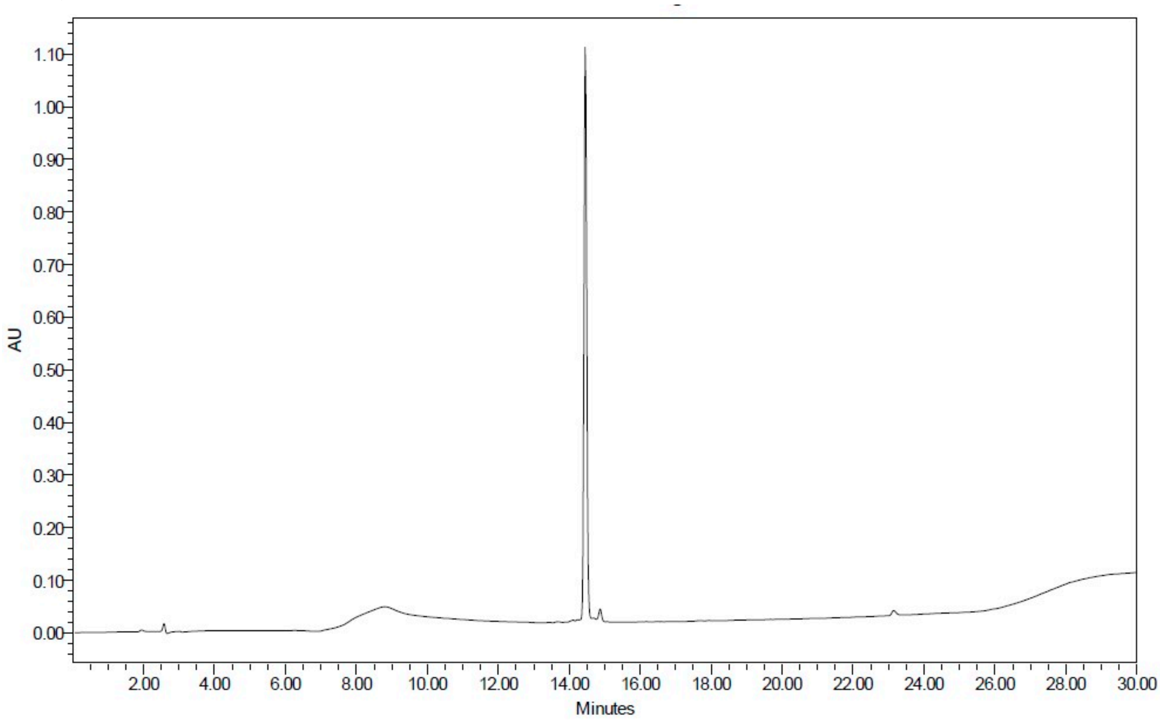

4
Retention time: 14.72 min
Purity: 99 %

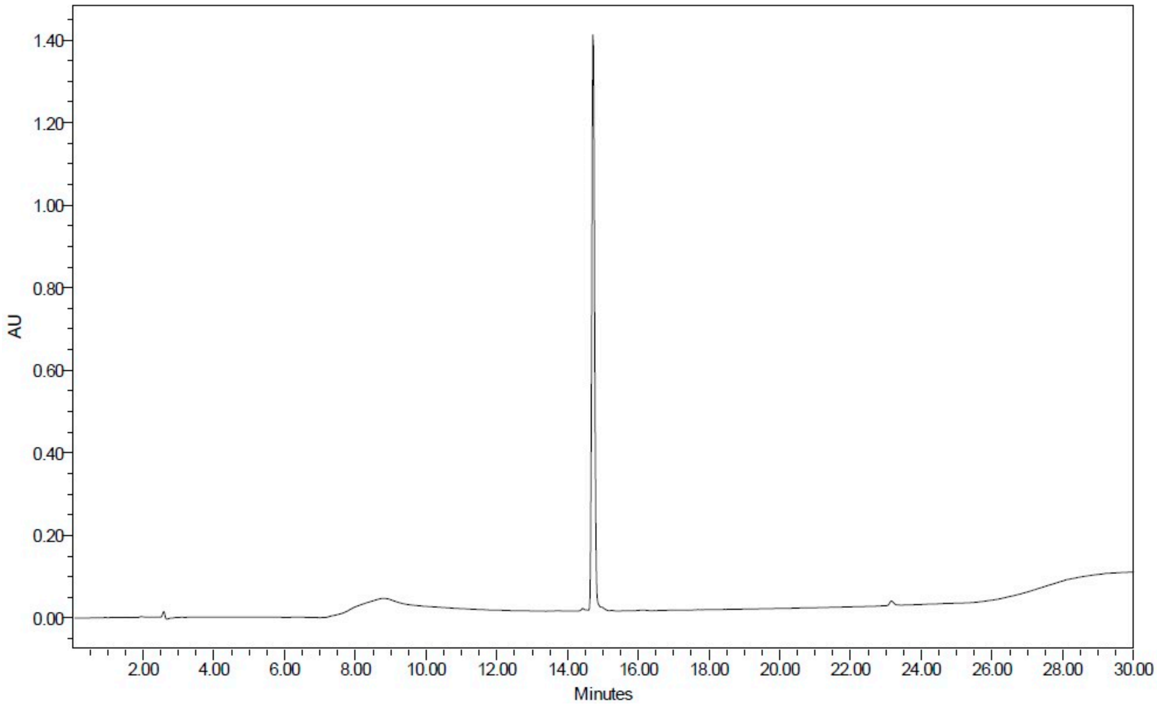

5
Retention time: 16.49 min
Purity: 98 %

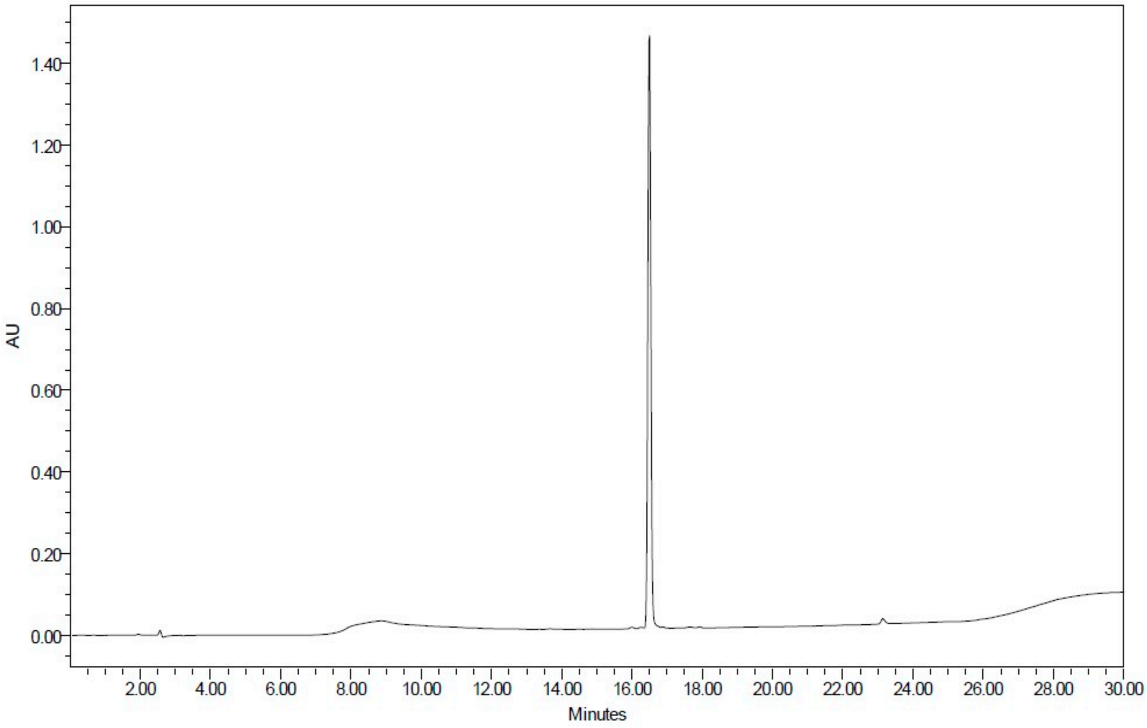

6
Retention time: 13.39 min
Purity: 100 %

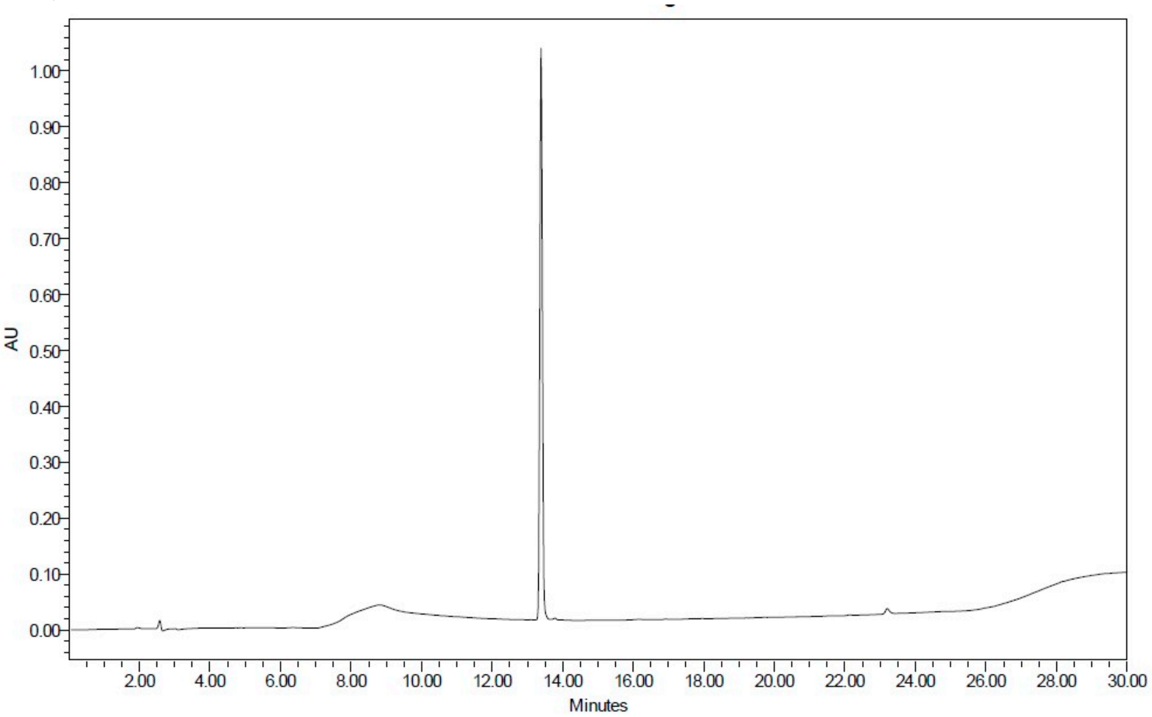

7
Retention time: 16.45 min
Purity: 99 %

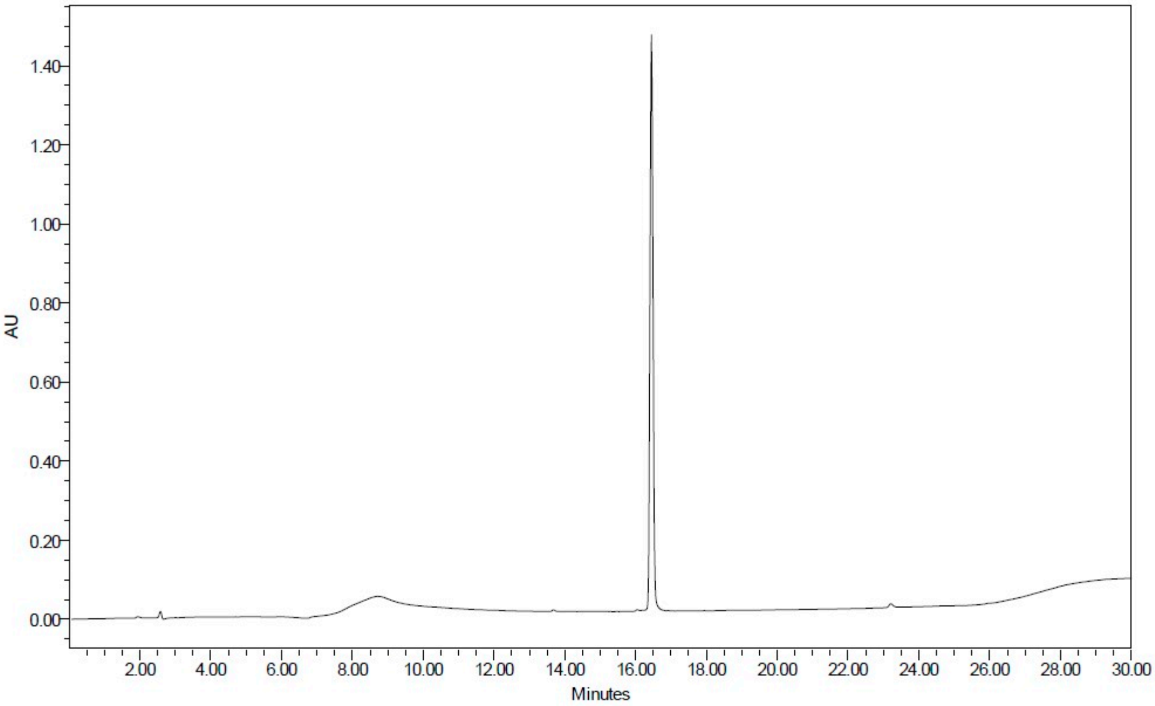

8
Retention time: 14.47 min
Purity: 98 %

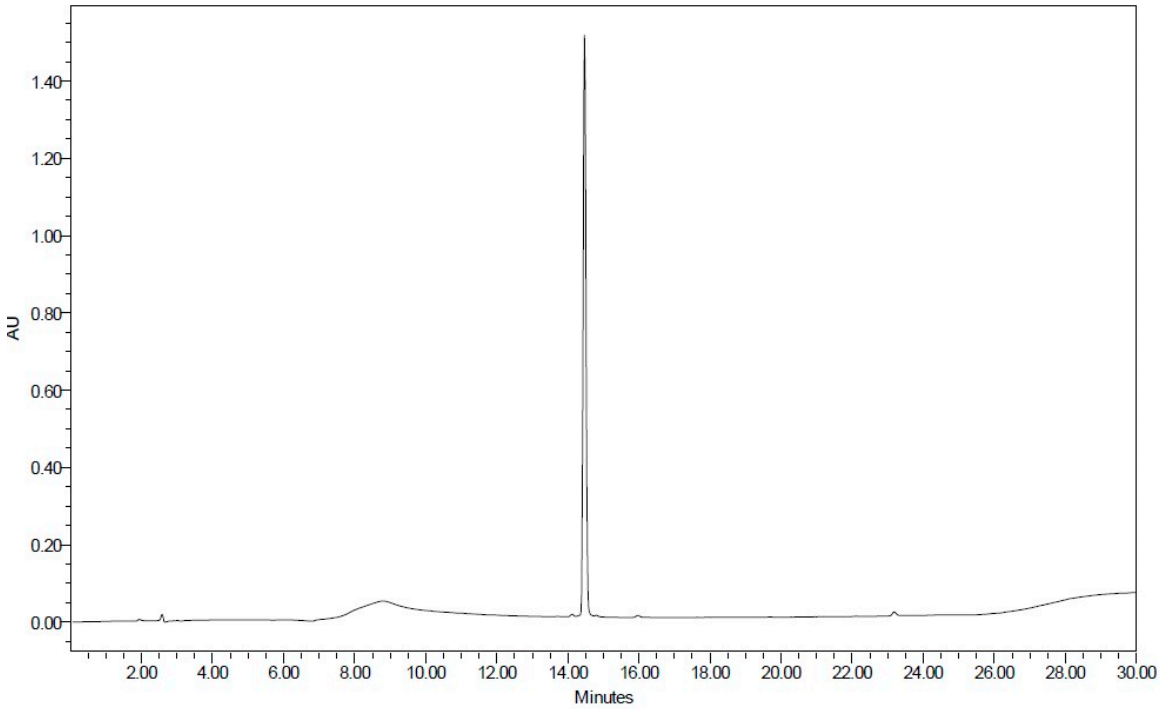

9
Retention time: 18.24 min
Purity: 98 %

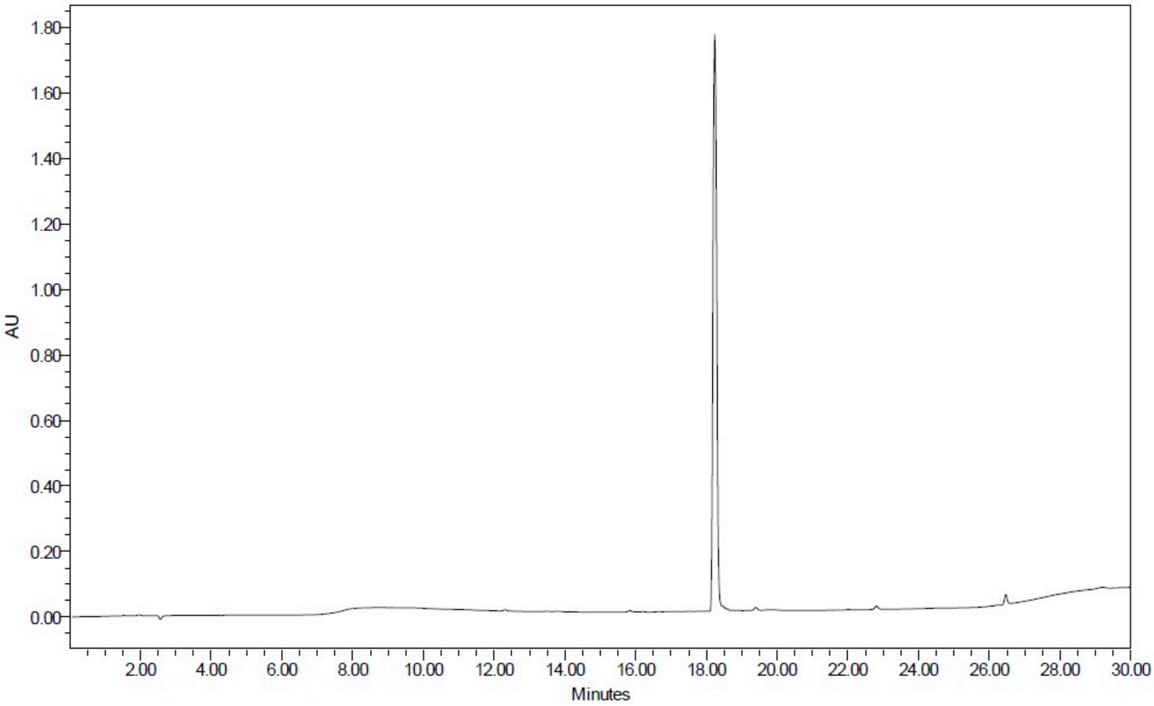

10
Retention time: 16.00 min
Purity: 98 %

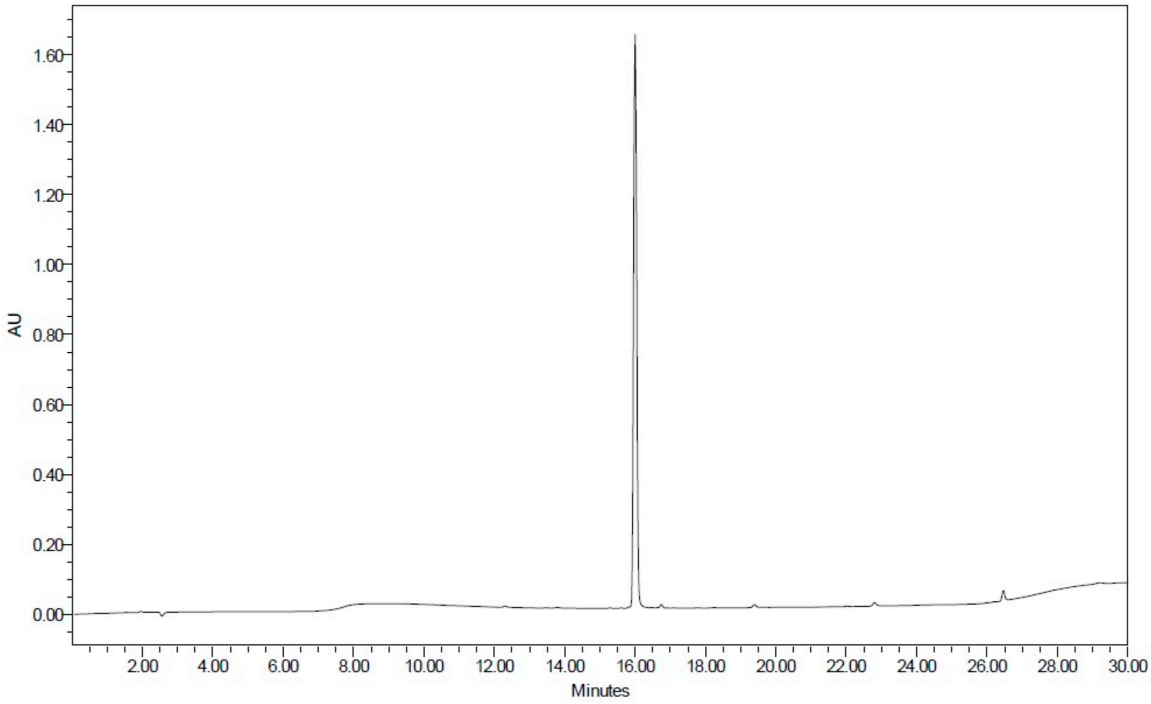

11
Retention time: 17.45 min
Purity: 98 %

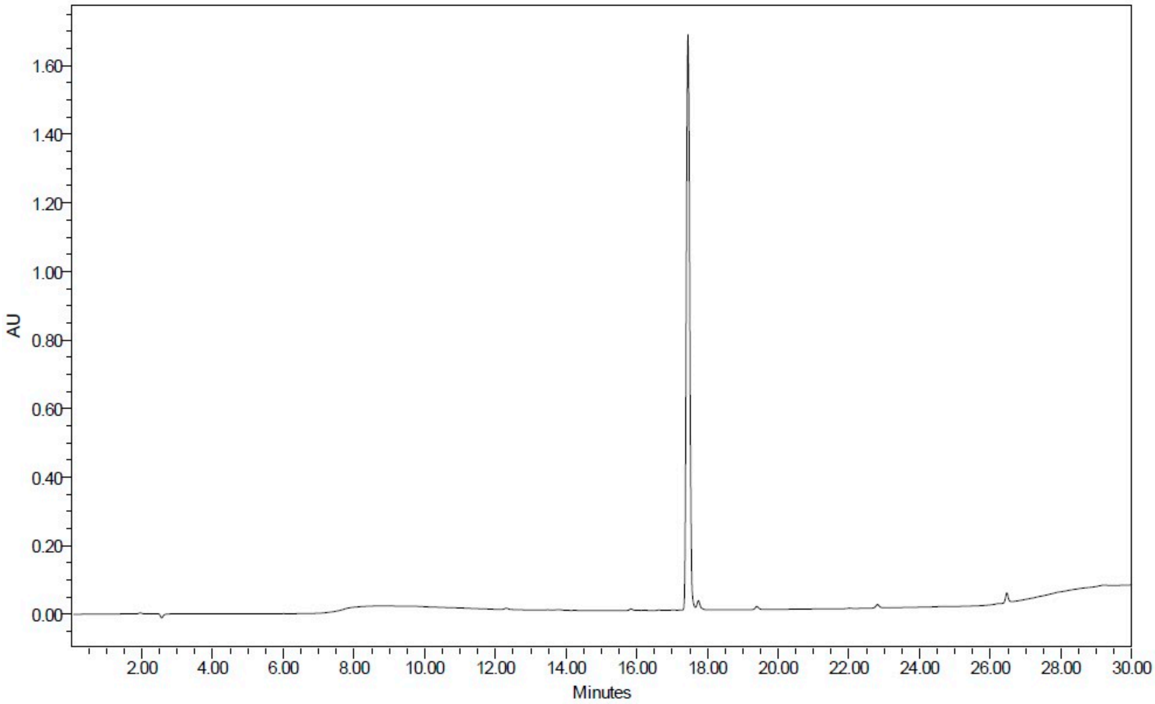

12
Retention time: 17.43 min
Purity: 98 %

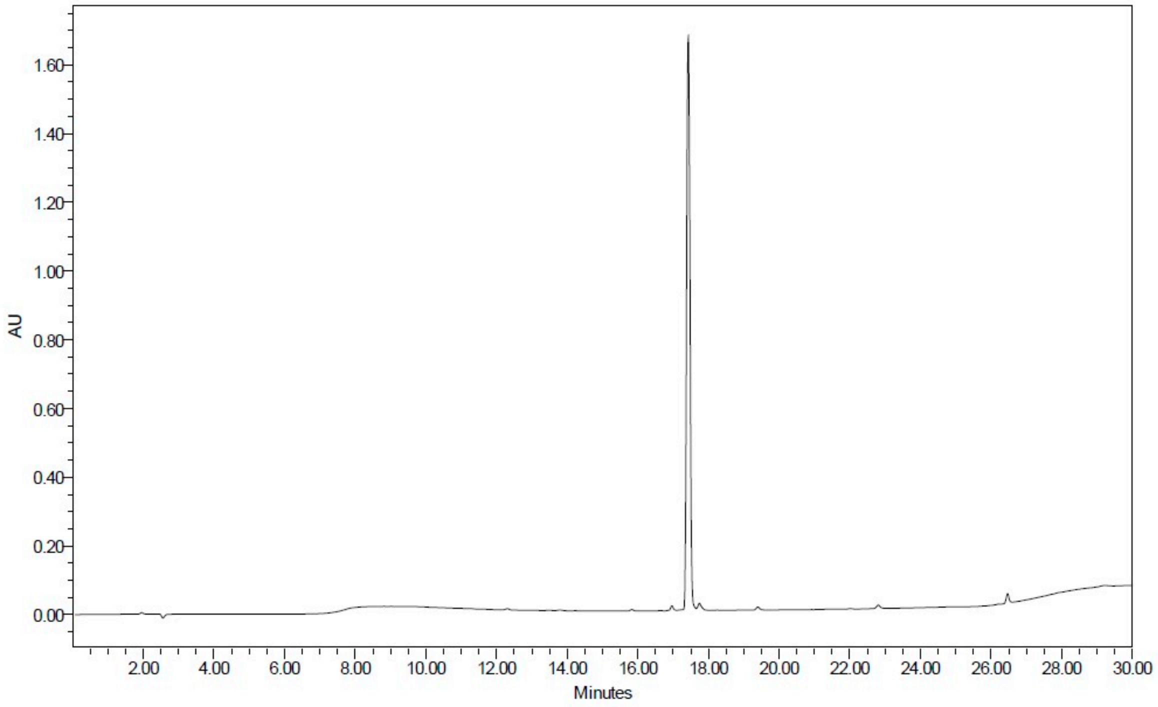

13
Retention time: 17.90 min
Purity: 96 %

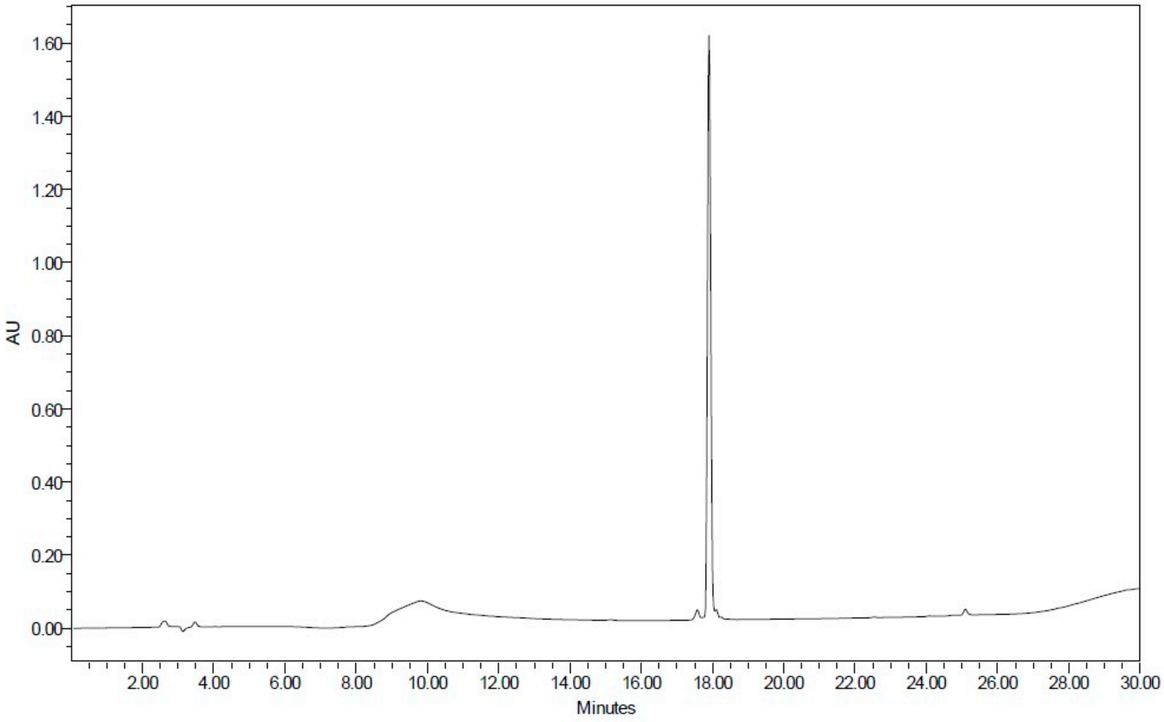

14
Retention time: 18.03 min
Purity: 96 %

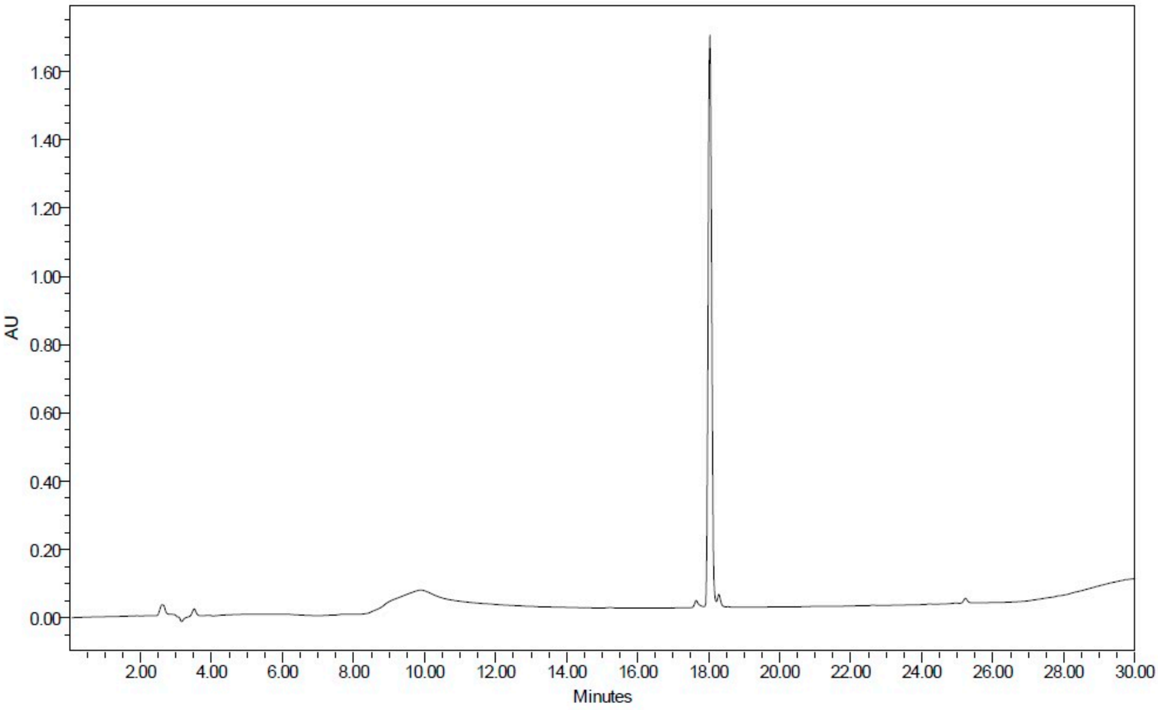

15
Retention time: 17.11 min
Purity: 95 %

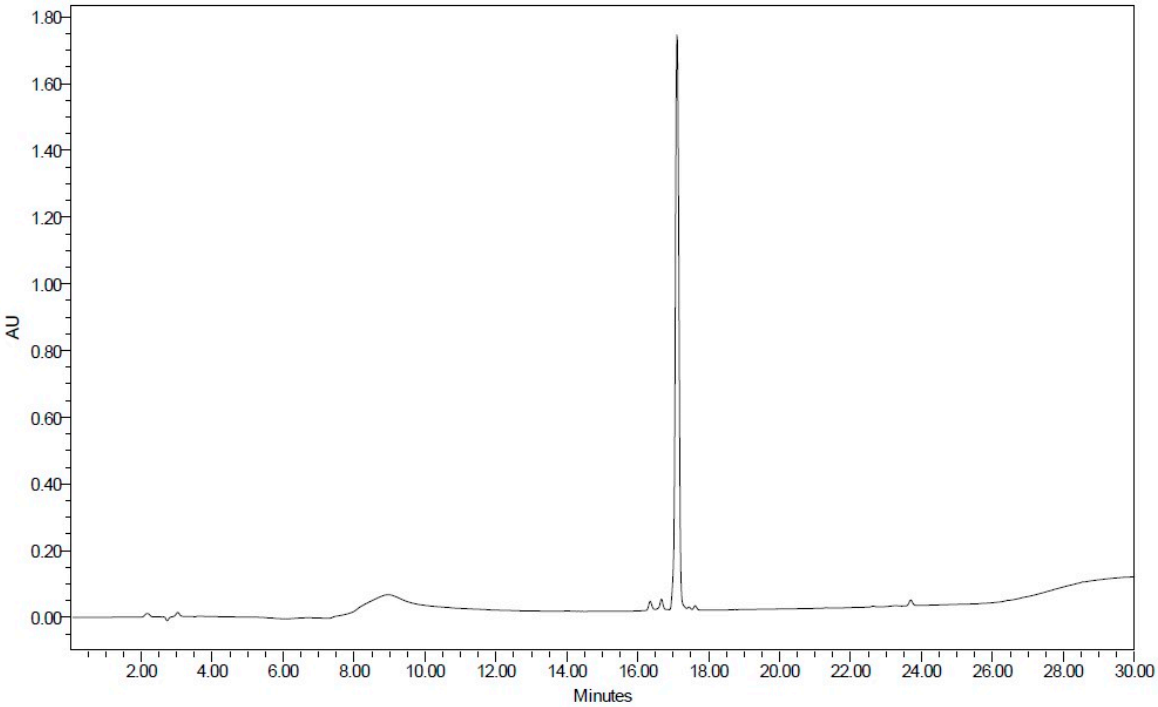

16
Retention time: 17.31 min
Purity: 97 %

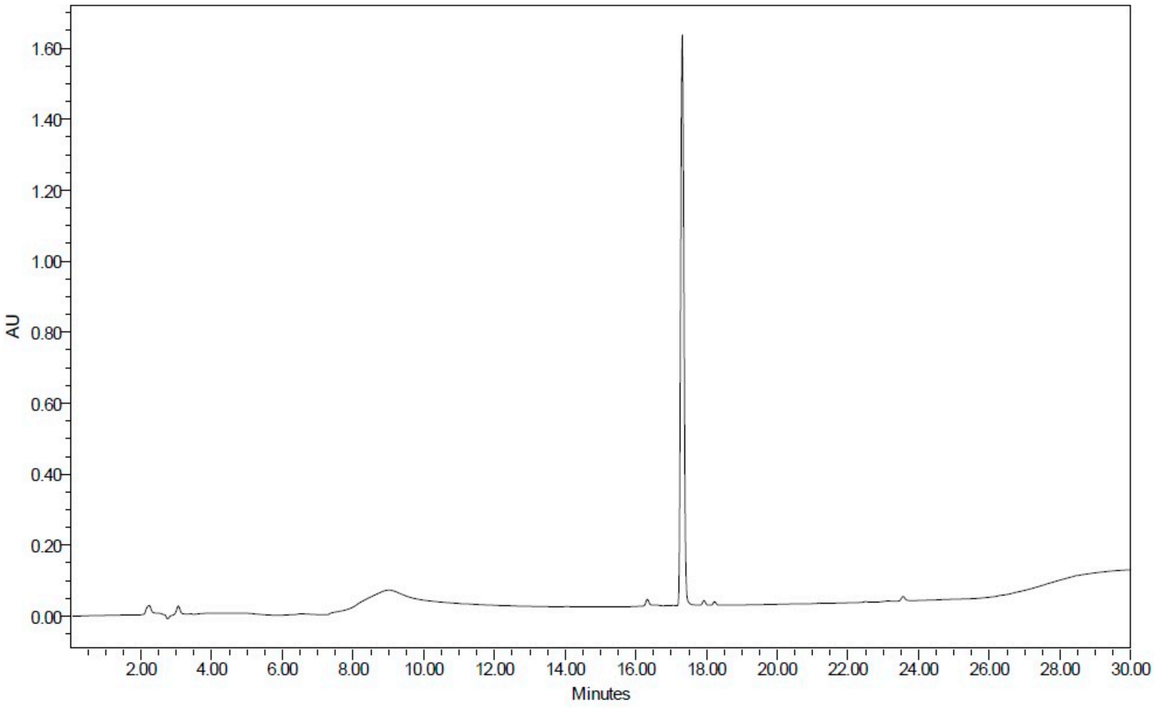

17
Retention time: 18.45 min
Purity: 98 %

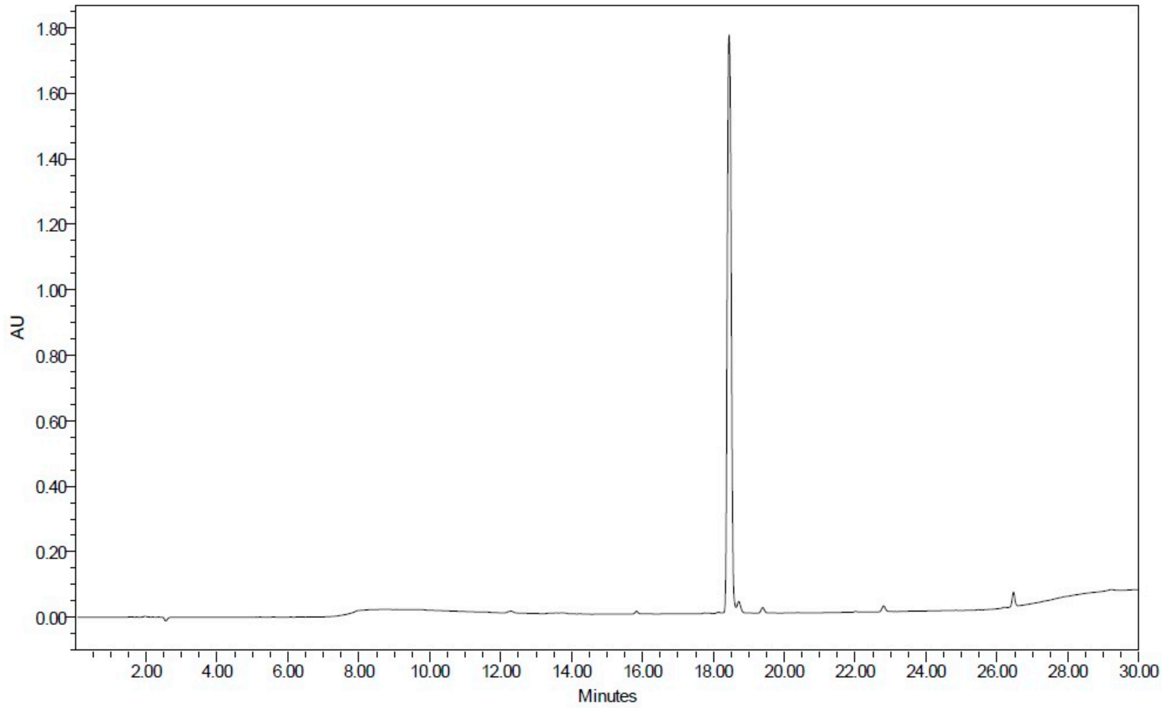

18
Retention time: 15.63 min
Purity: 95 %

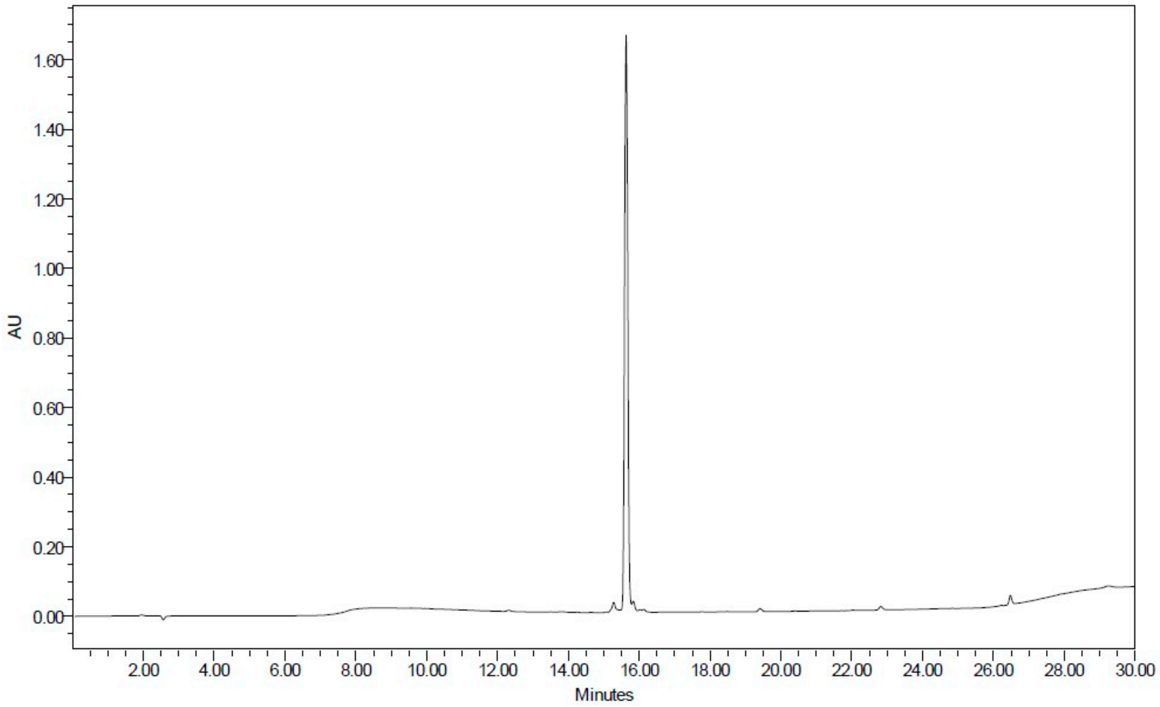

19
Retention time: 17.41 min
Purity: 97 %

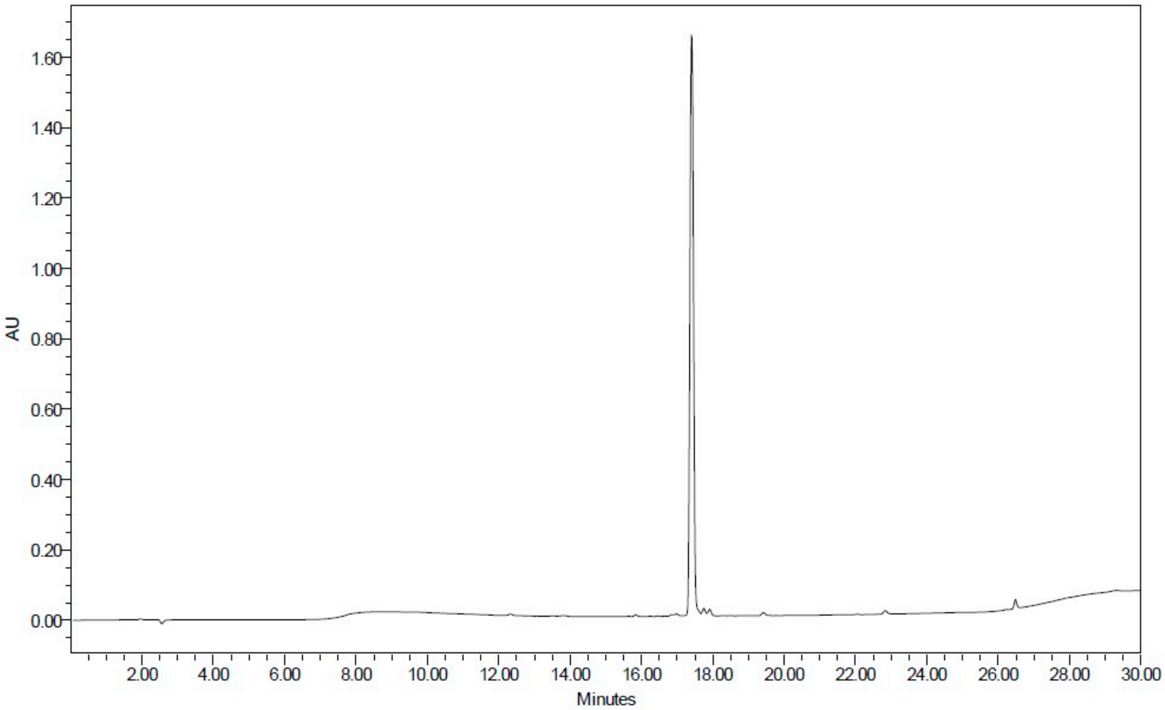

20
Retention time: 17.50 min
Purity: 96 %

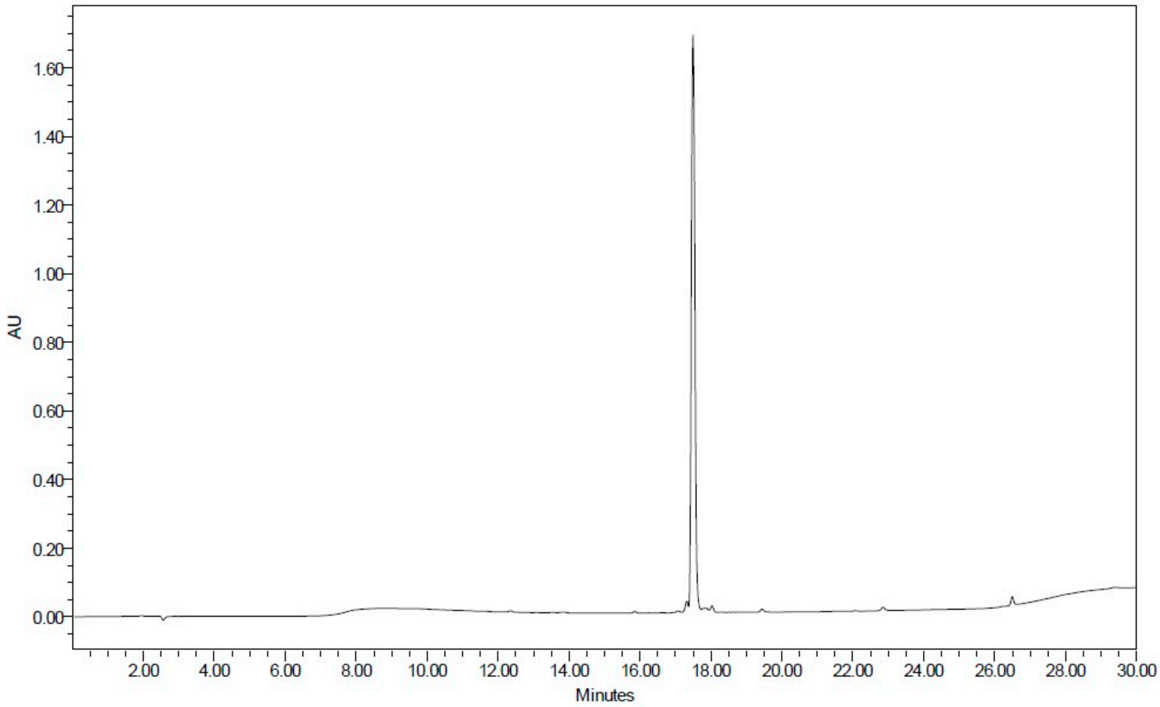

21
Retention time: 16.34 min
Purity: 96 %

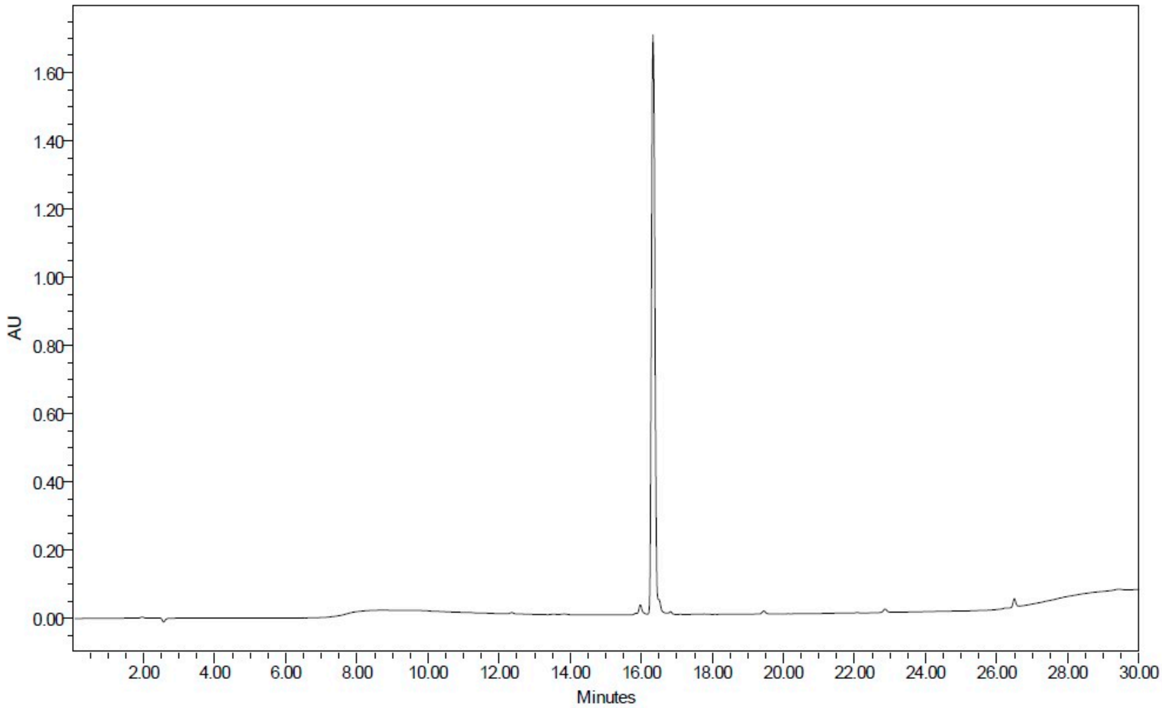

22
Retention time: 16.44 min
Purity: 96 %

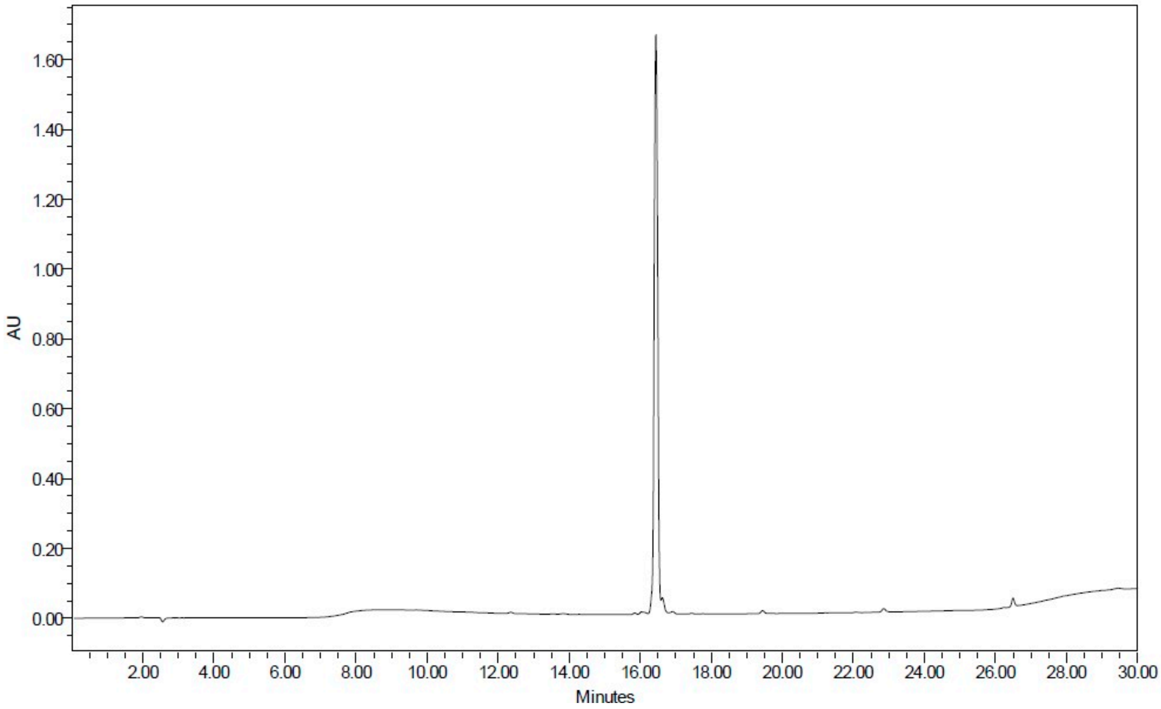

23
Retention time: 16.66 min
Purity: 93 %

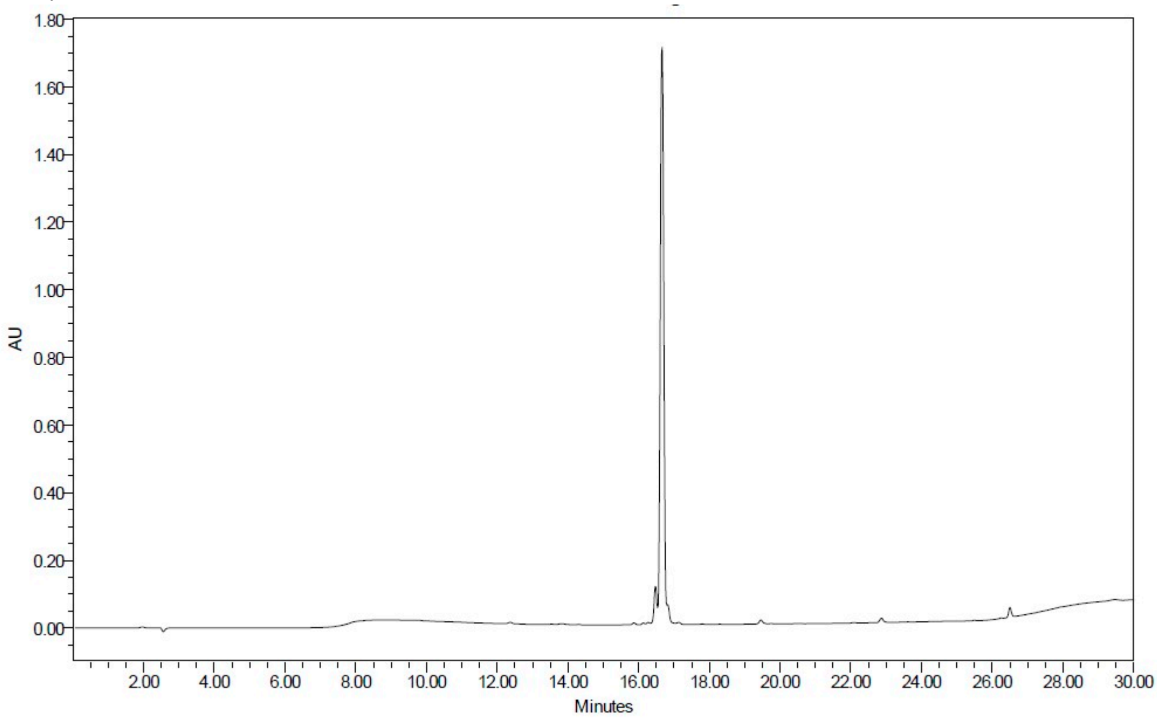

24
Retention time: 16.84 min
Purity: 95 %

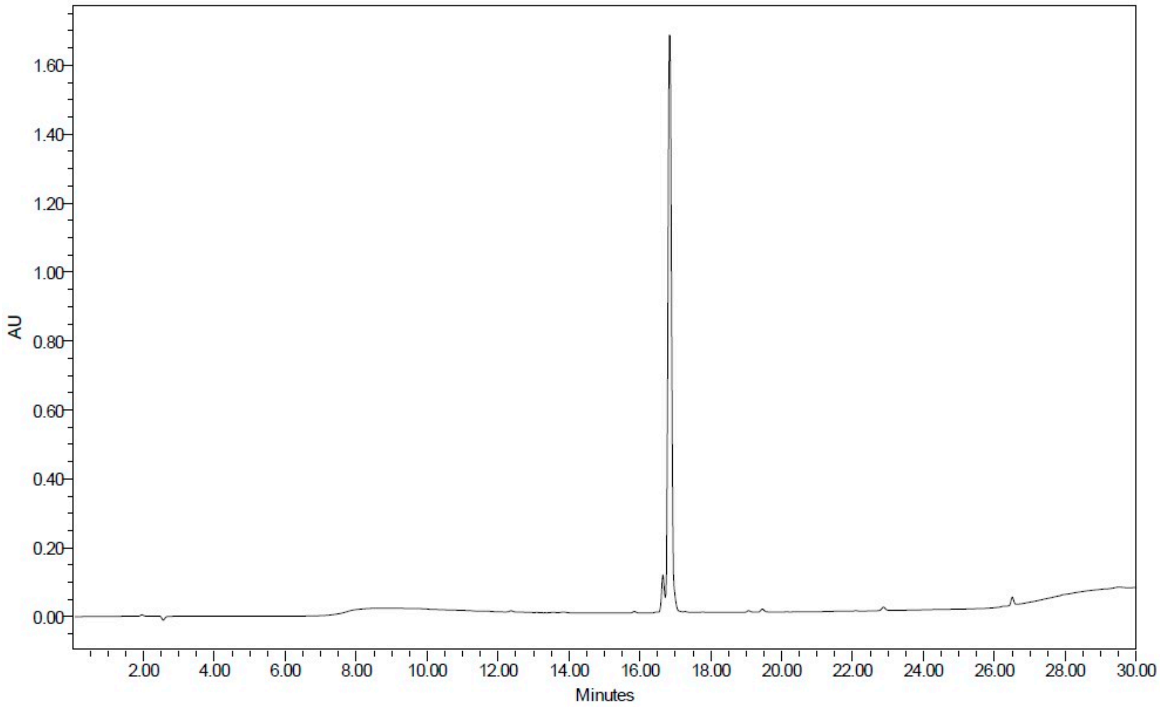

25
Retention time: 15.48 min
Purity: 98 %

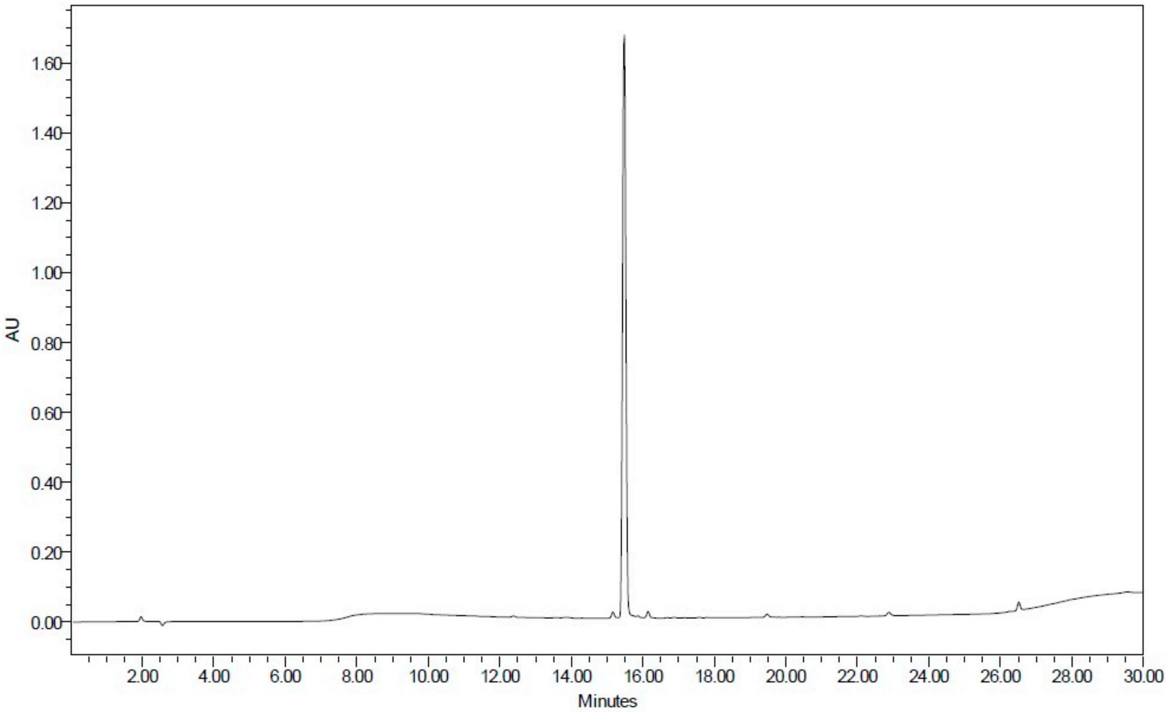

26

Retention time: 15.46 min

Purity: 99 %

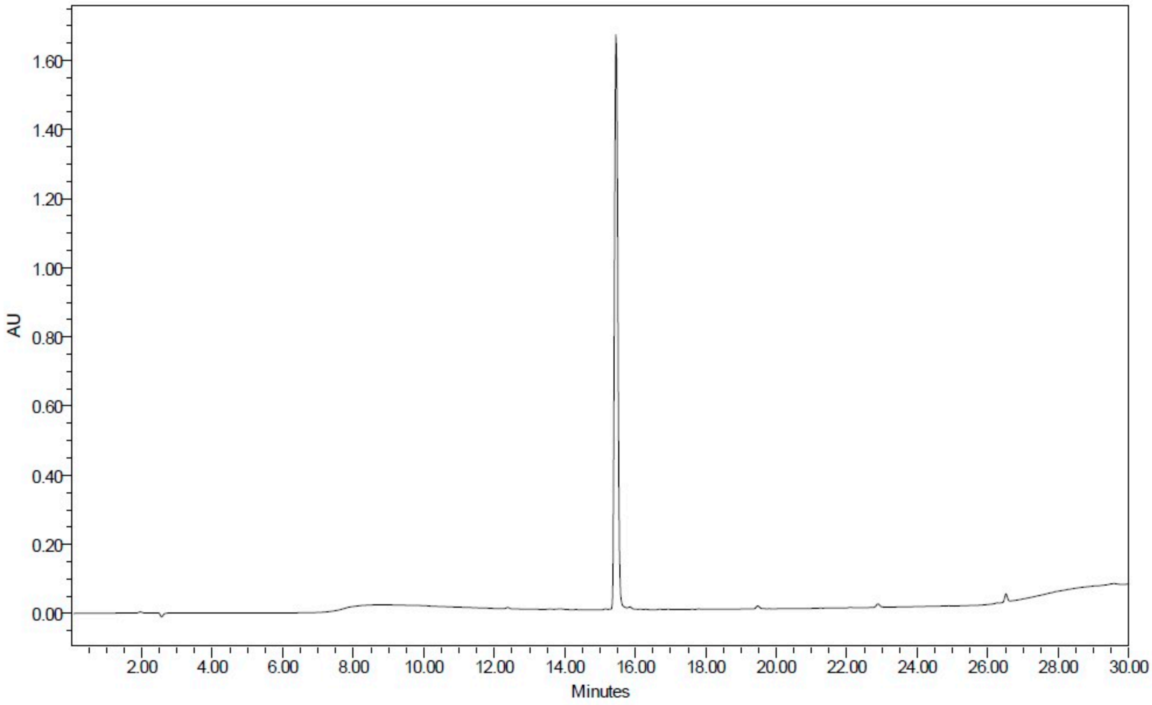

27

Retention time: 16.42 min

Purity: 97 %

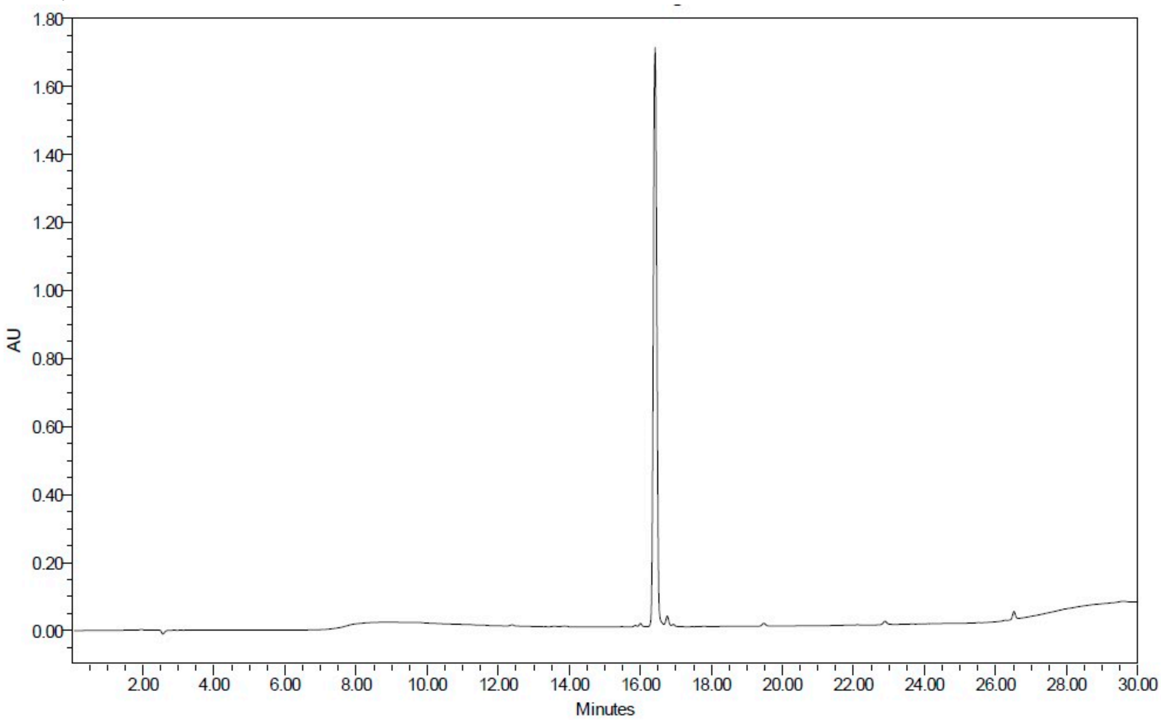

28
Retention time: 17.22 min
Purity: 97 %

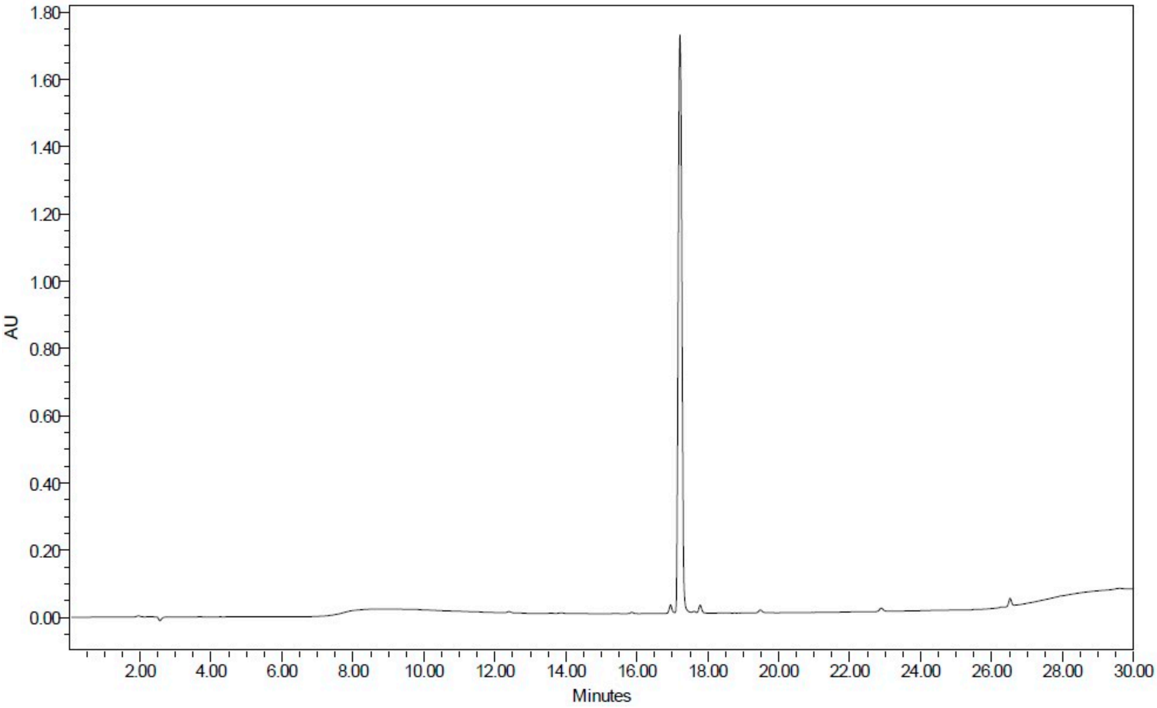

29
Retention time: 15.56 min
Purity: 99 %

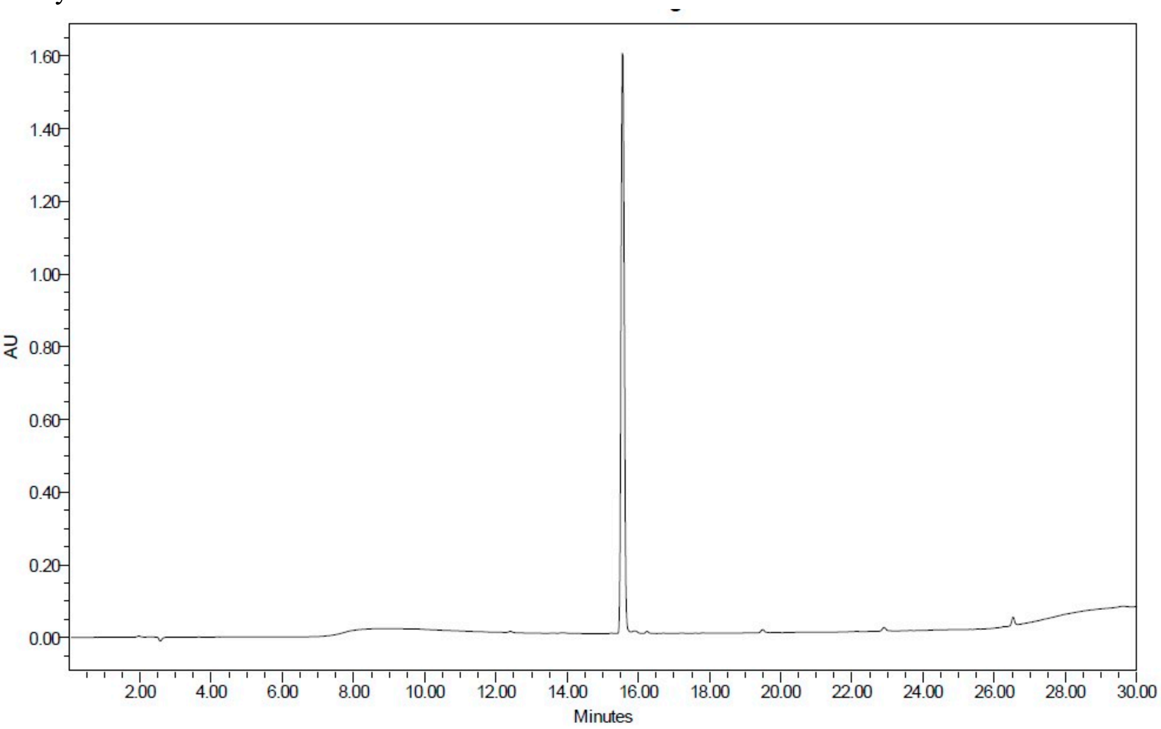

30
Retention time: 14.47 min
Purity: 94 %

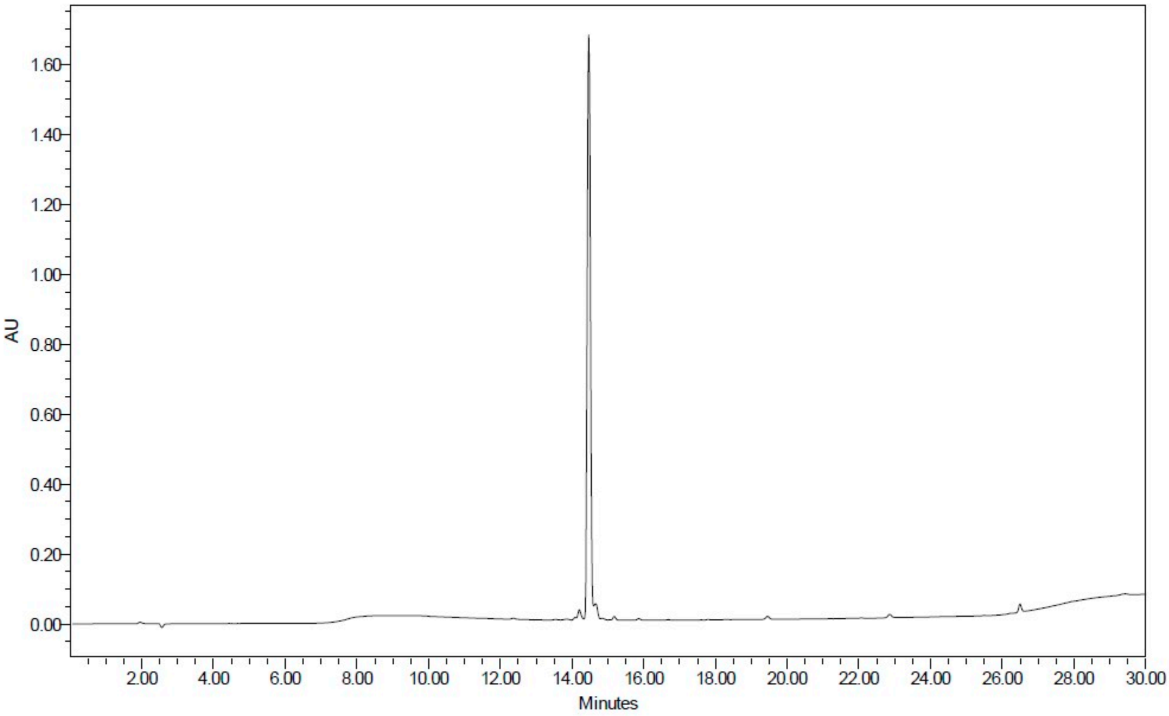

31
Retention time: 15.59 min
Purity: 96 %

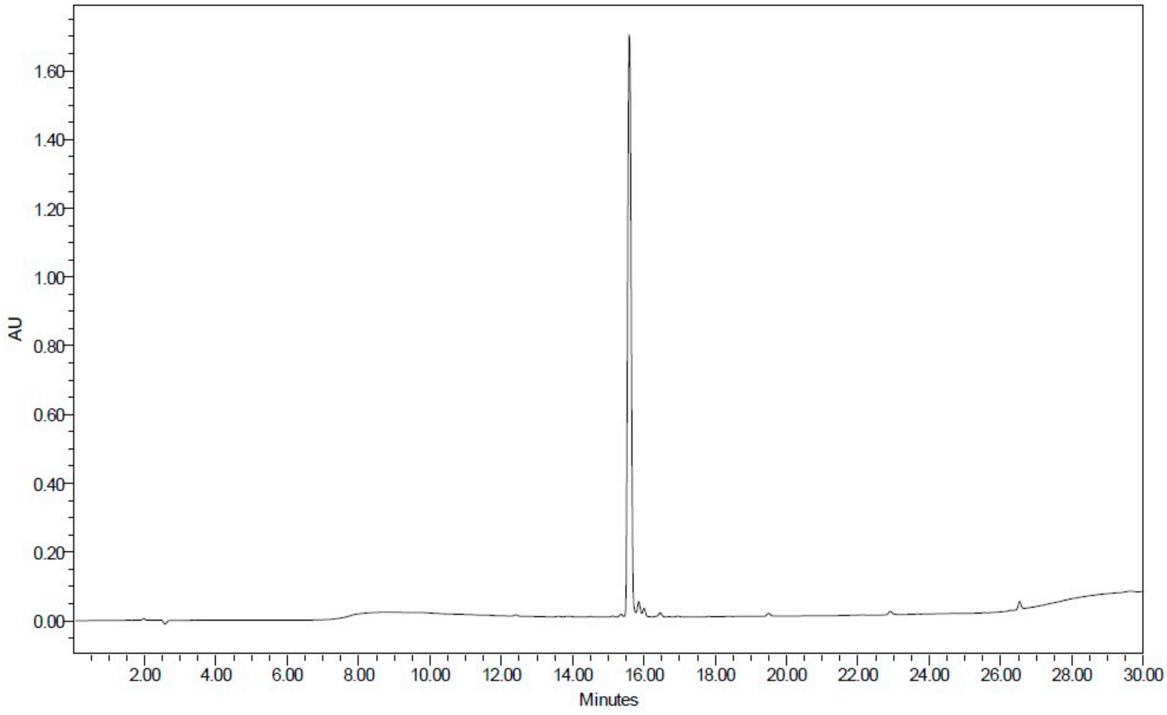

32
Retention time: 13.05 min
Purity: 98 %

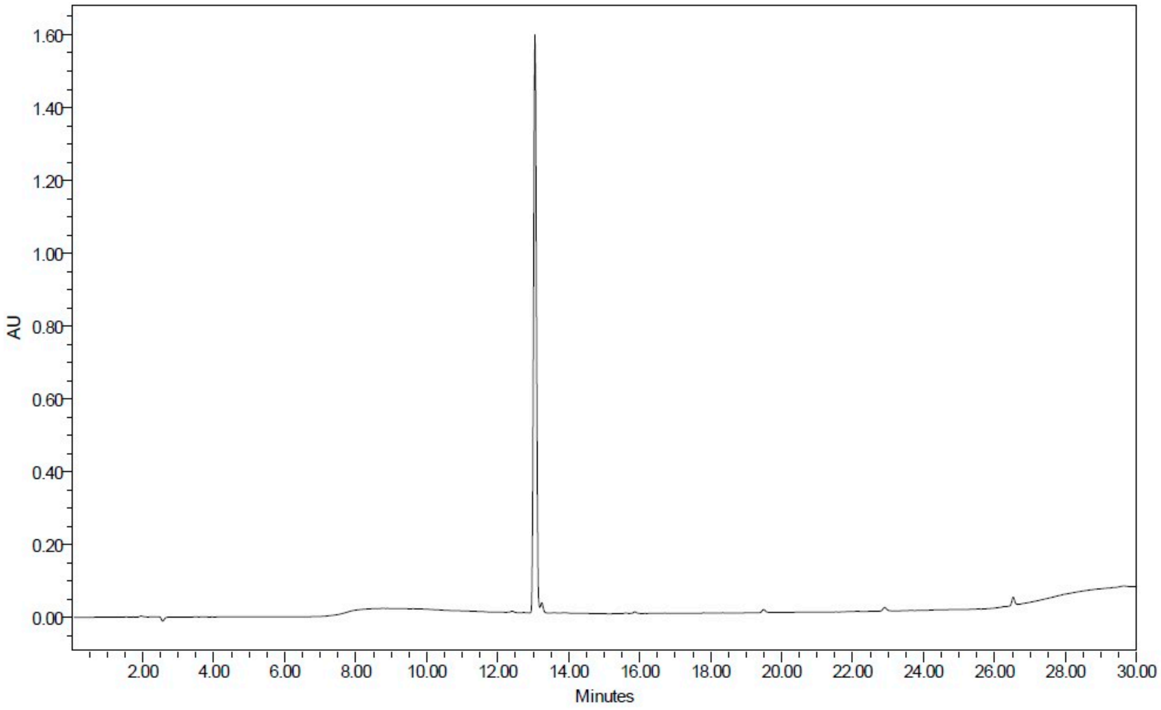

33
Retention time: 15.72 min
Purity: 97 %

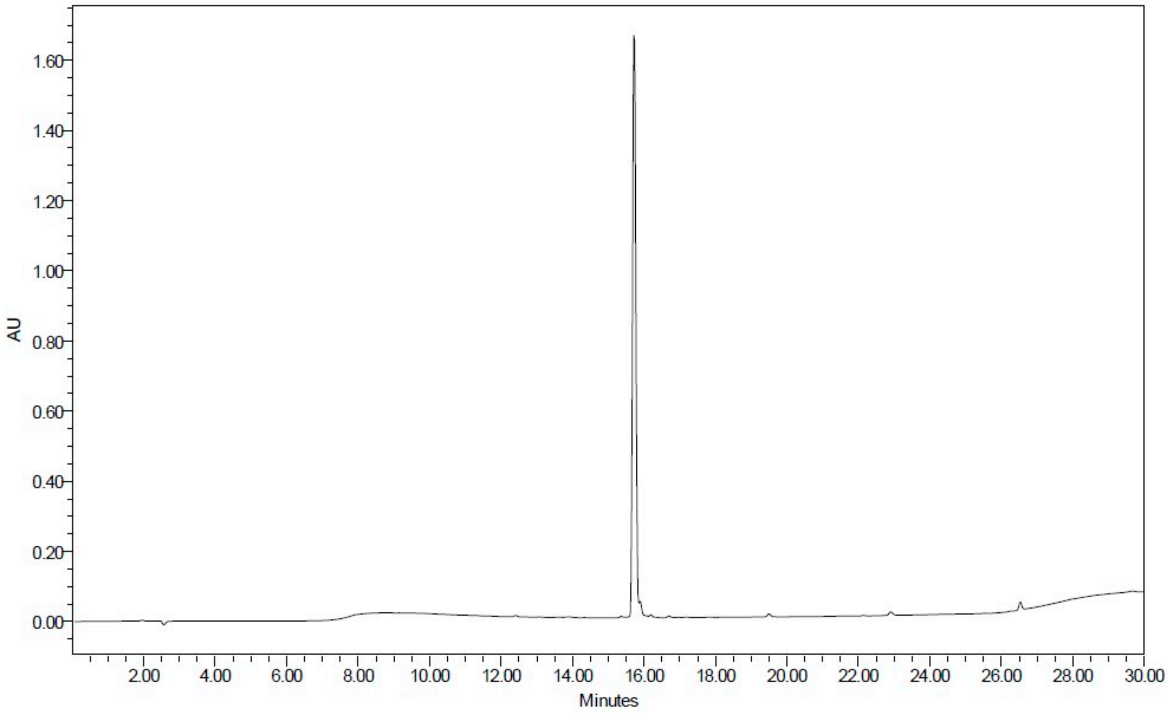

34
Retention time: 15.77 min
Purity: 98 %

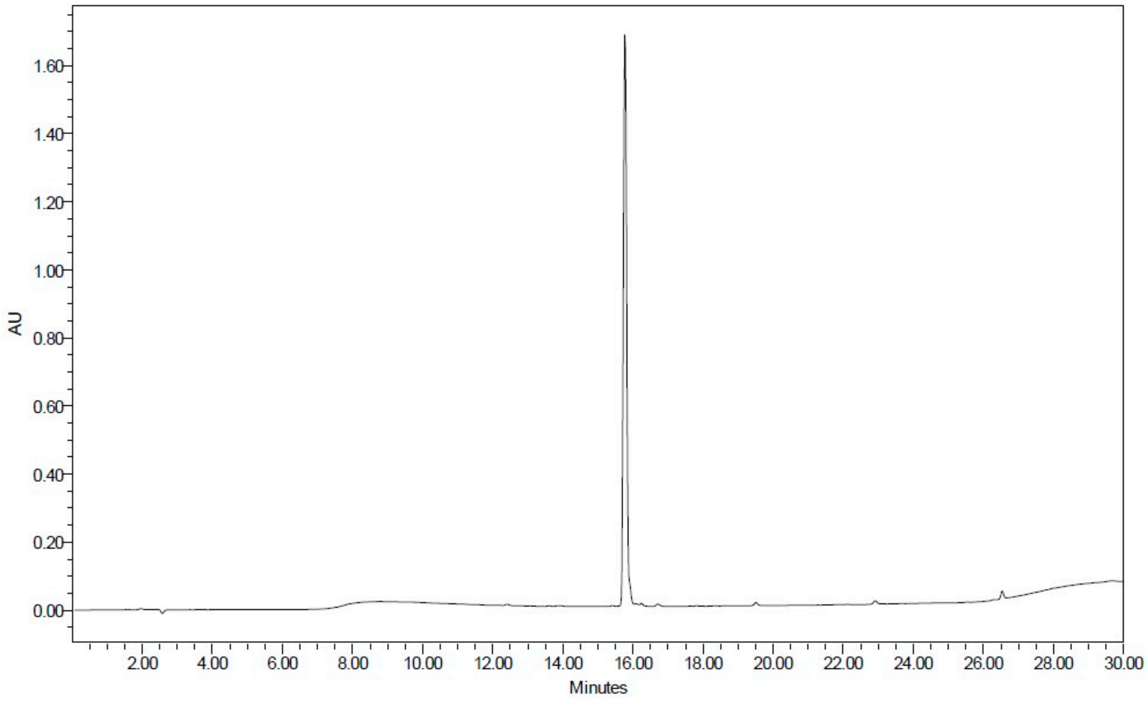

35
Retention time: 15.71 min
Purity: 97 %

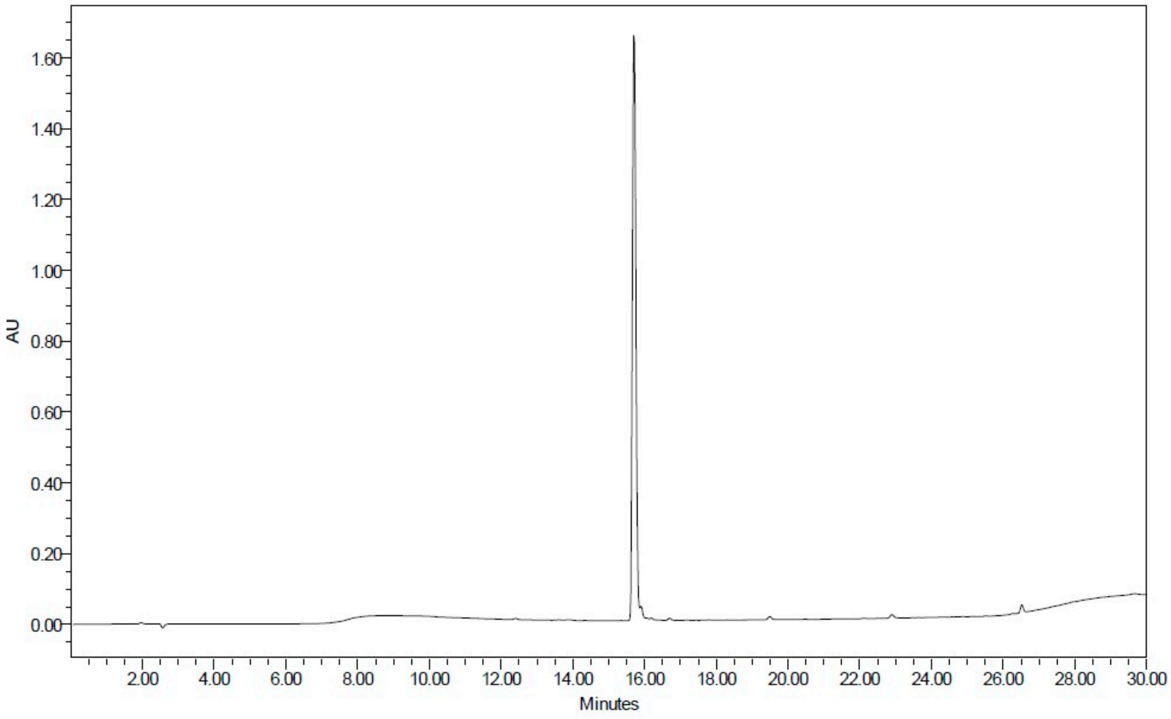

36

Retention time: 15.73 min

Purity: 97 %

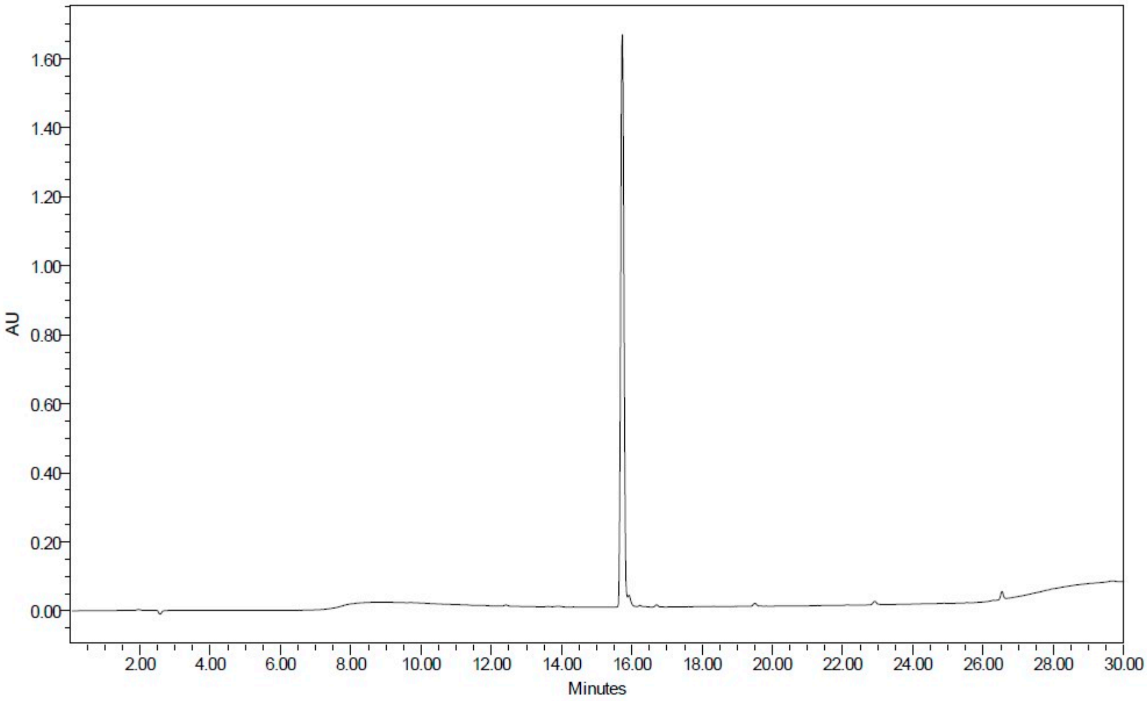

MALDI-TOF-MS spectra

D2-D
Mw: 1184.54
[MH$^+$]: 1185.77

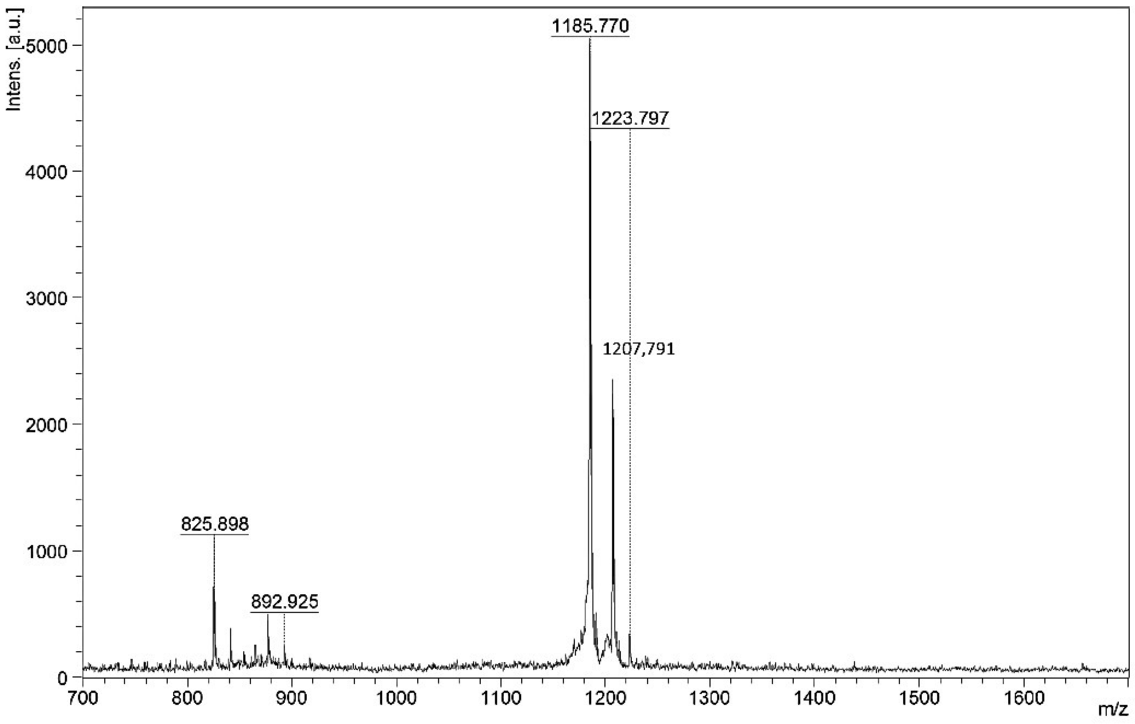

1
Mw: 1127.45
[MH$^+$]: 1128.54

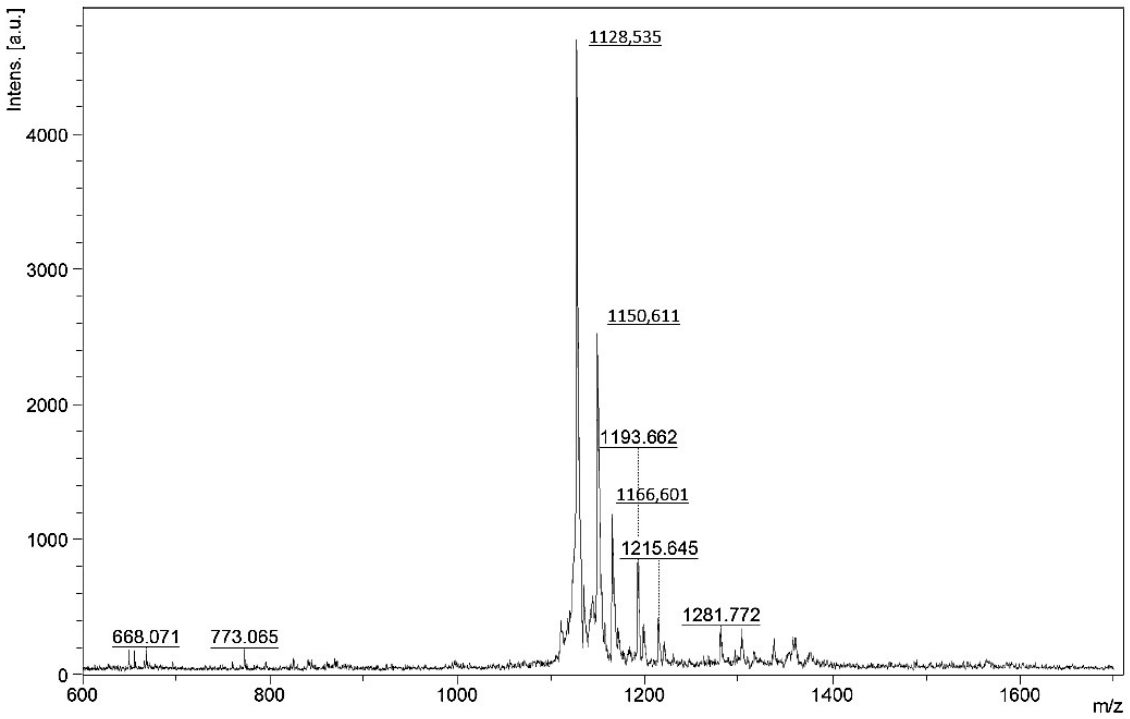

2
Mw: 1127.45
[MH$^+$]: 1128.56

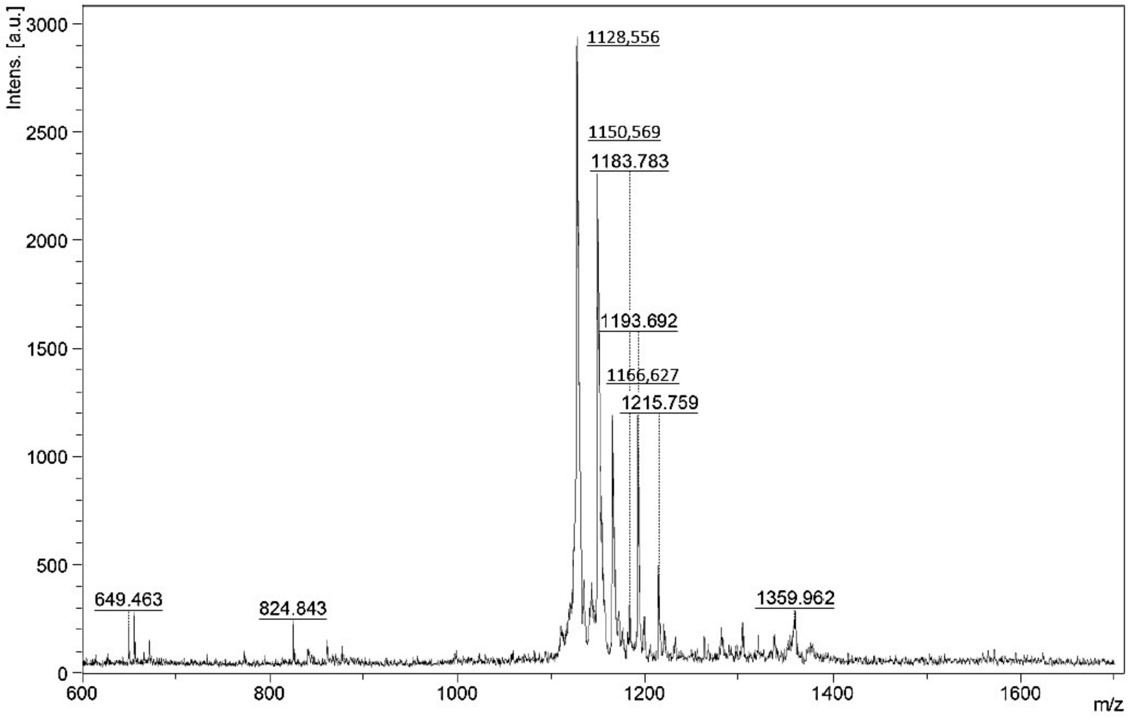

3
Mw: 1058.38
[MH$^+$]: 1059.49

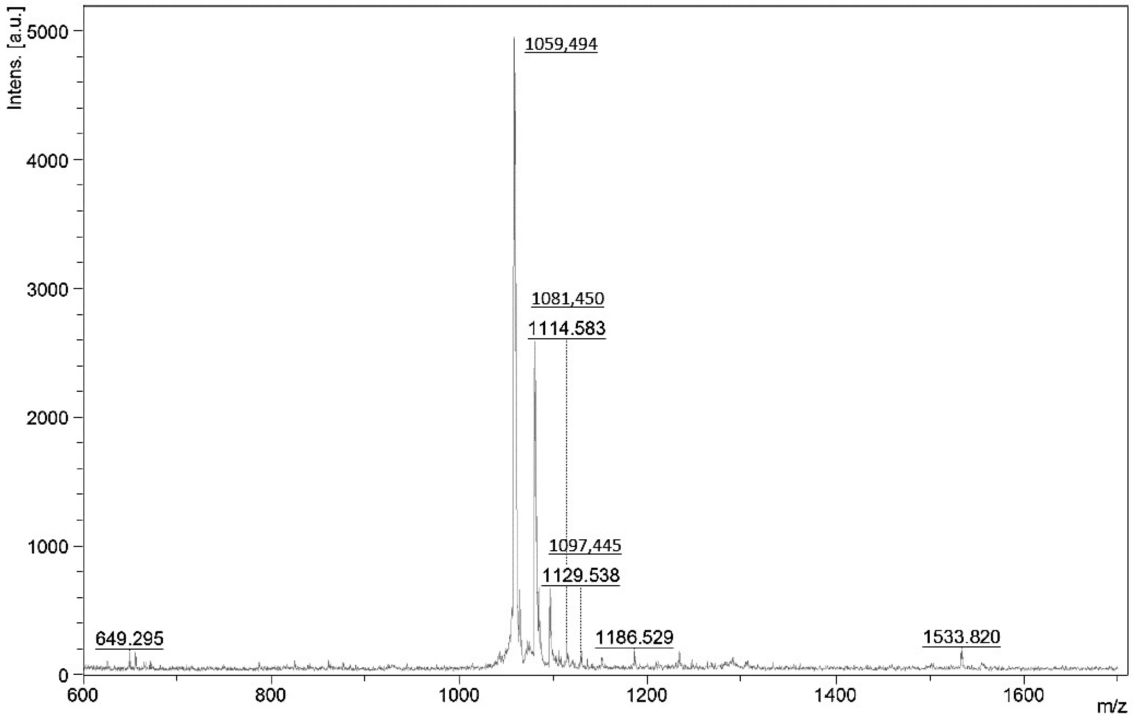

4

Mw: 1108.44

[MH+]: 1109.54

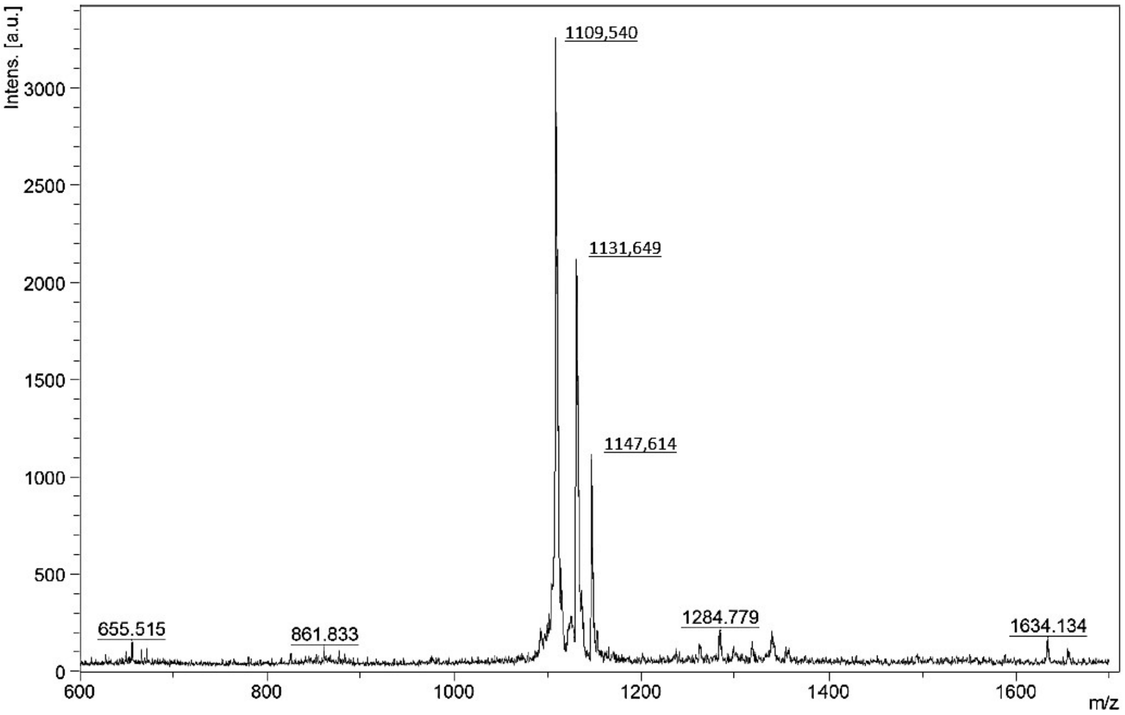

5

Mw: 1127.45

[MH+]: 1128.62

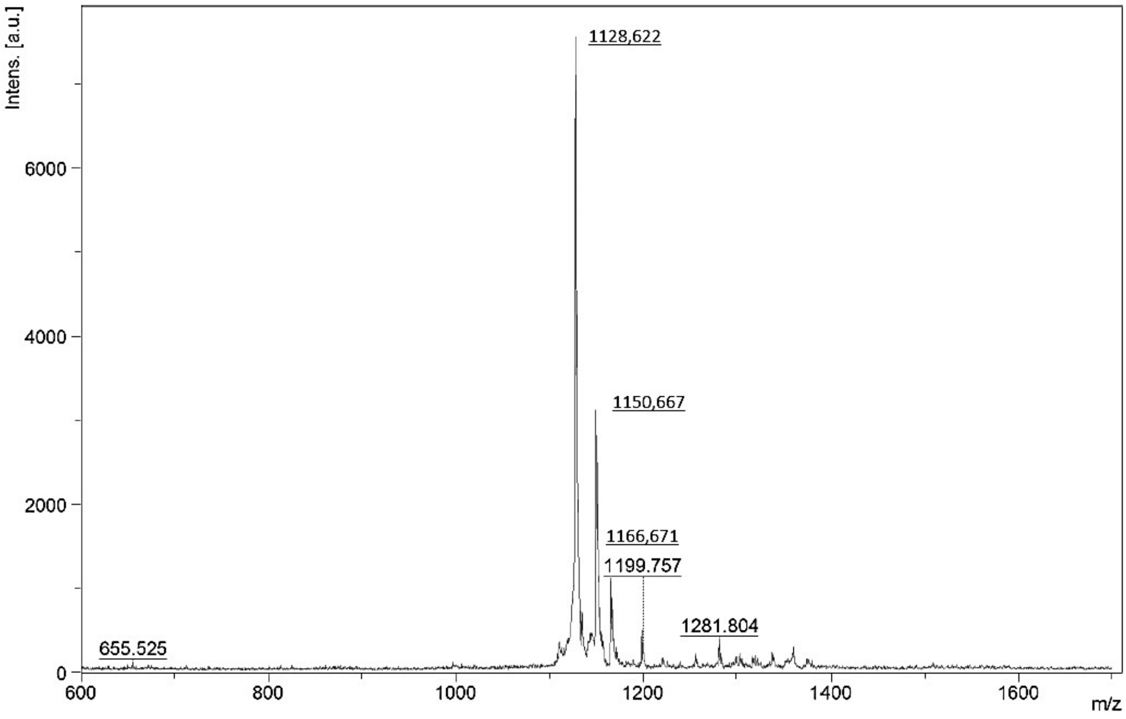

6

Mw: 1058.38

[MH+]: 1059.76

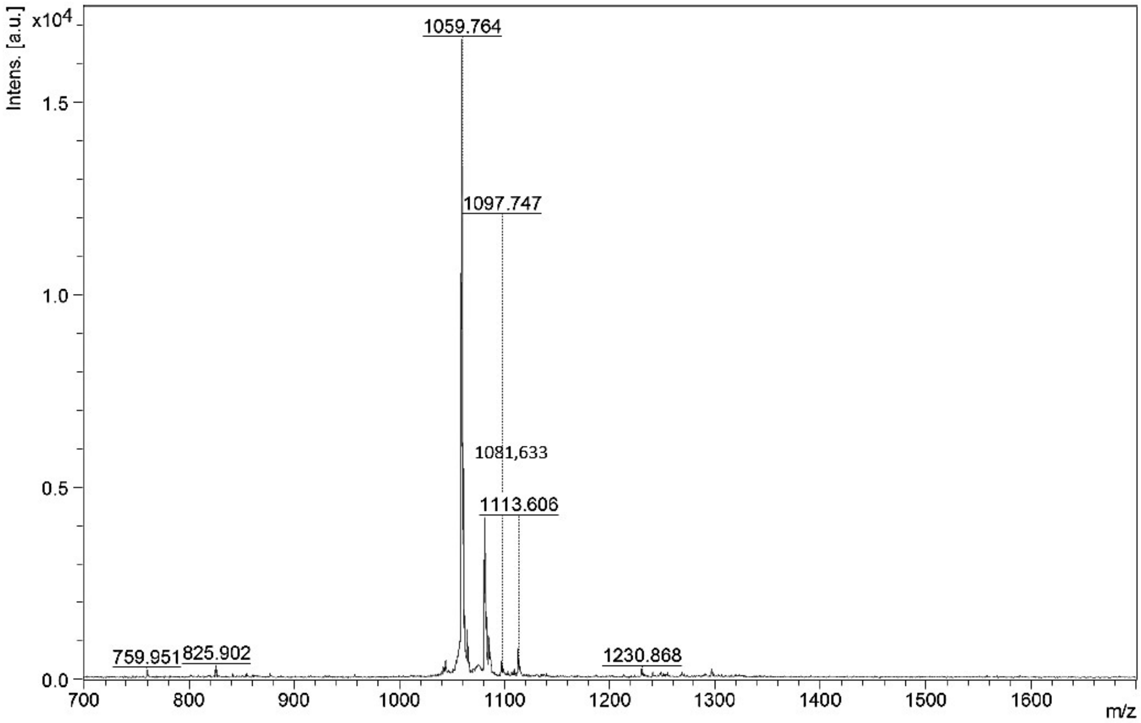

7

Mw: 1127.45

[MH+]: 1128.75

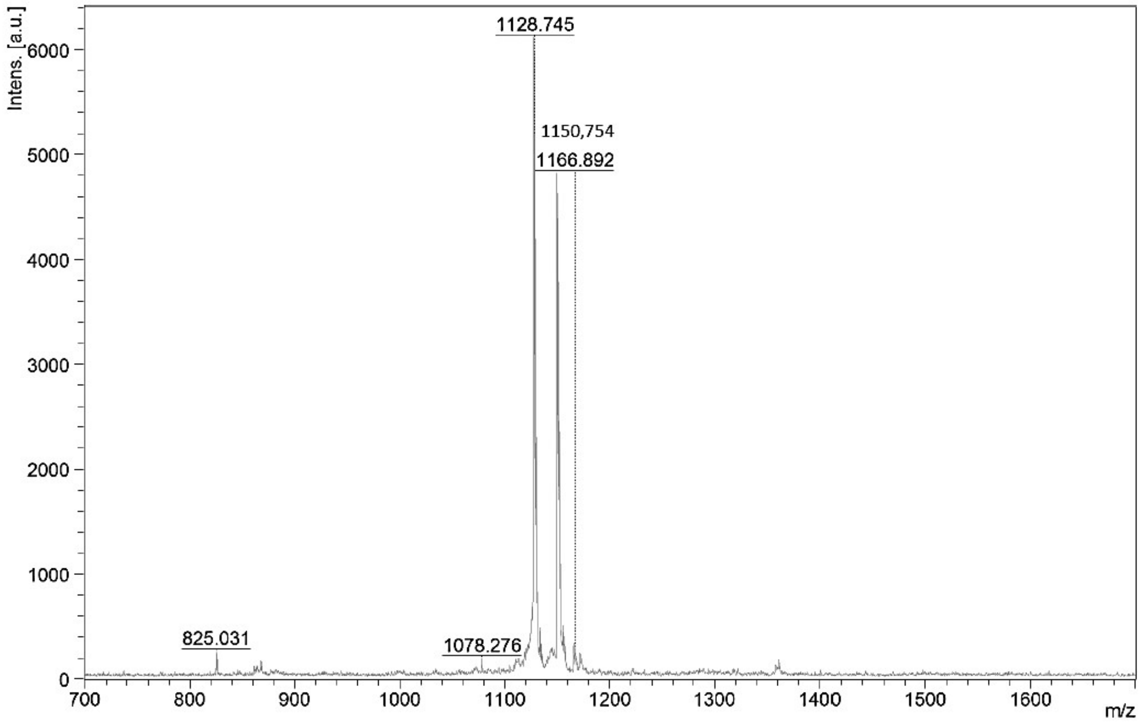

8

Mw: 1142.46

[MH+]: 1143.64

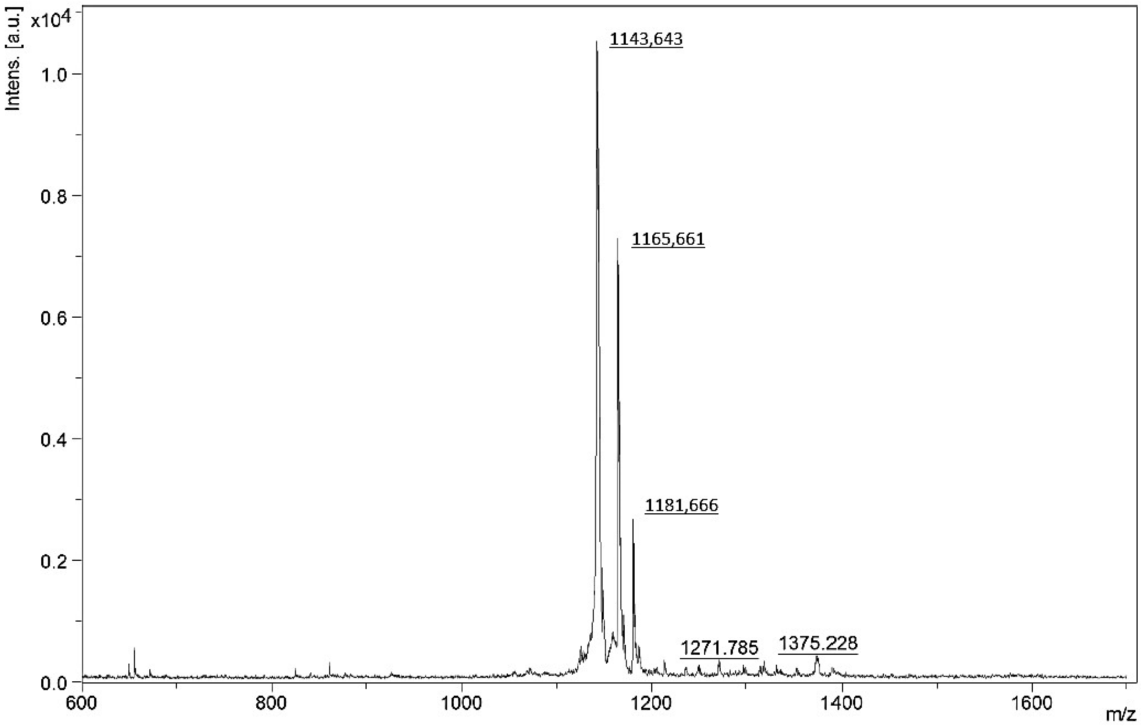

9

Mw: 1253.60

[MH+]: 1253.92

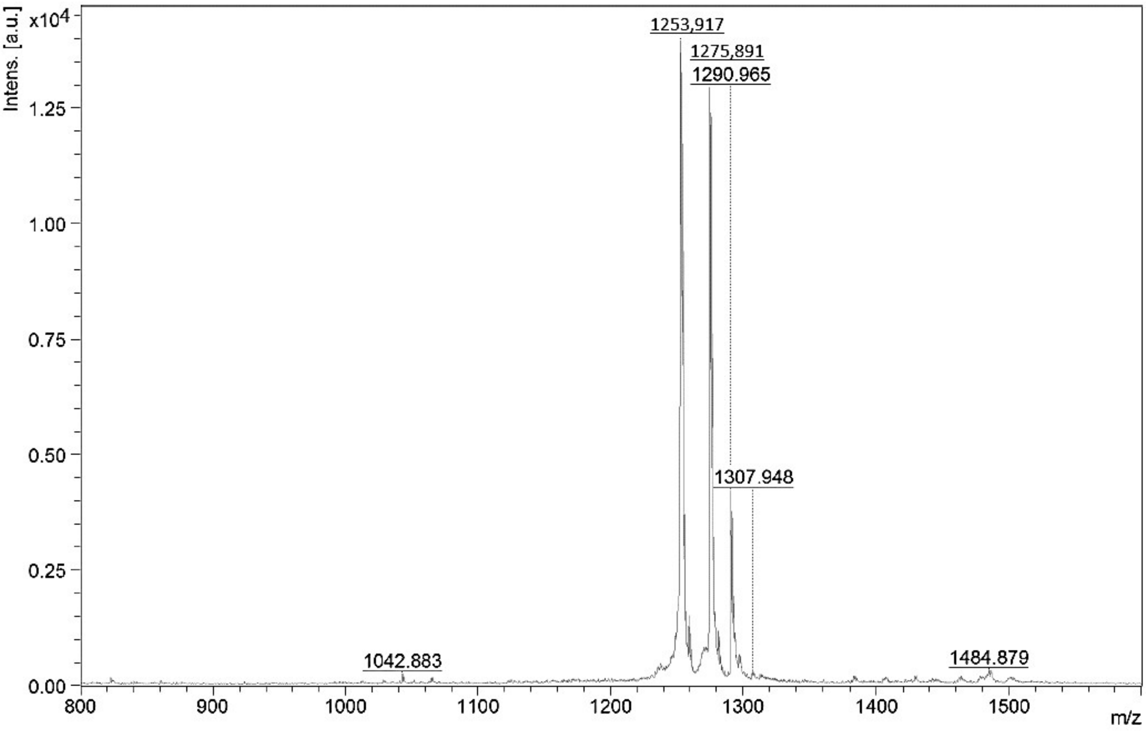

10
Mw: 1142.46
[MH+]: 1143.48

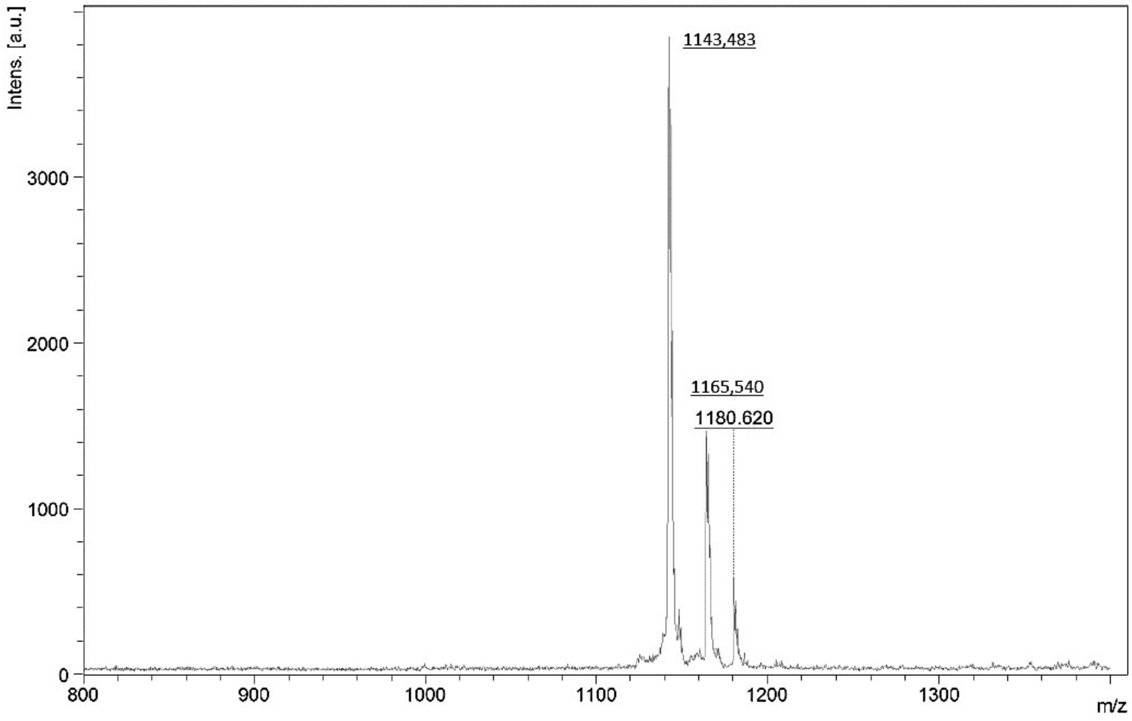

11
Mw: 1169.53
[MH+]: 1170.46

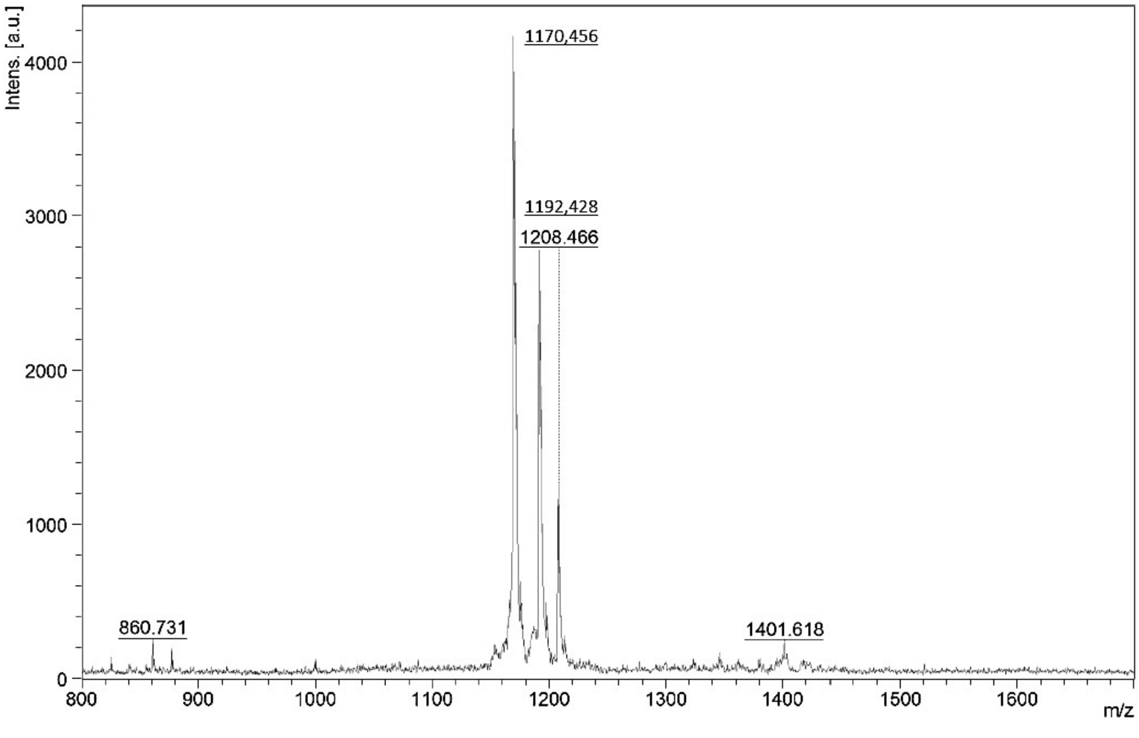

12

Mw: 1203.54

[MH+]: 1204.46

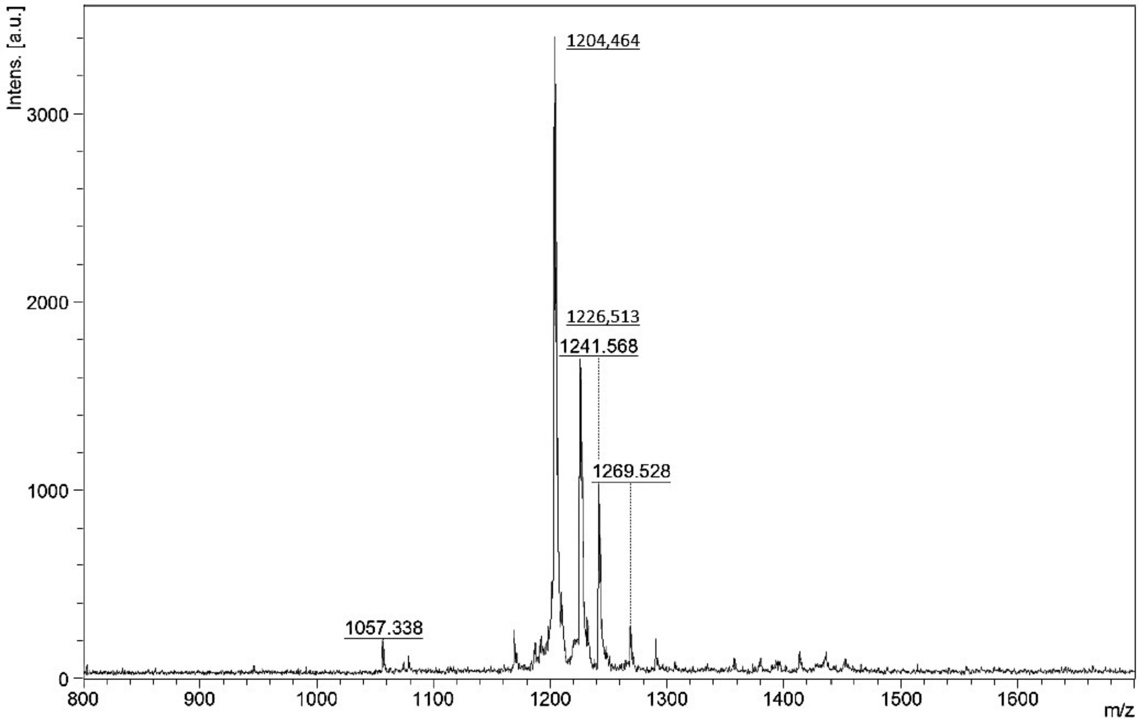

13

Mw: 1143.45

[MH+]: 1144.48

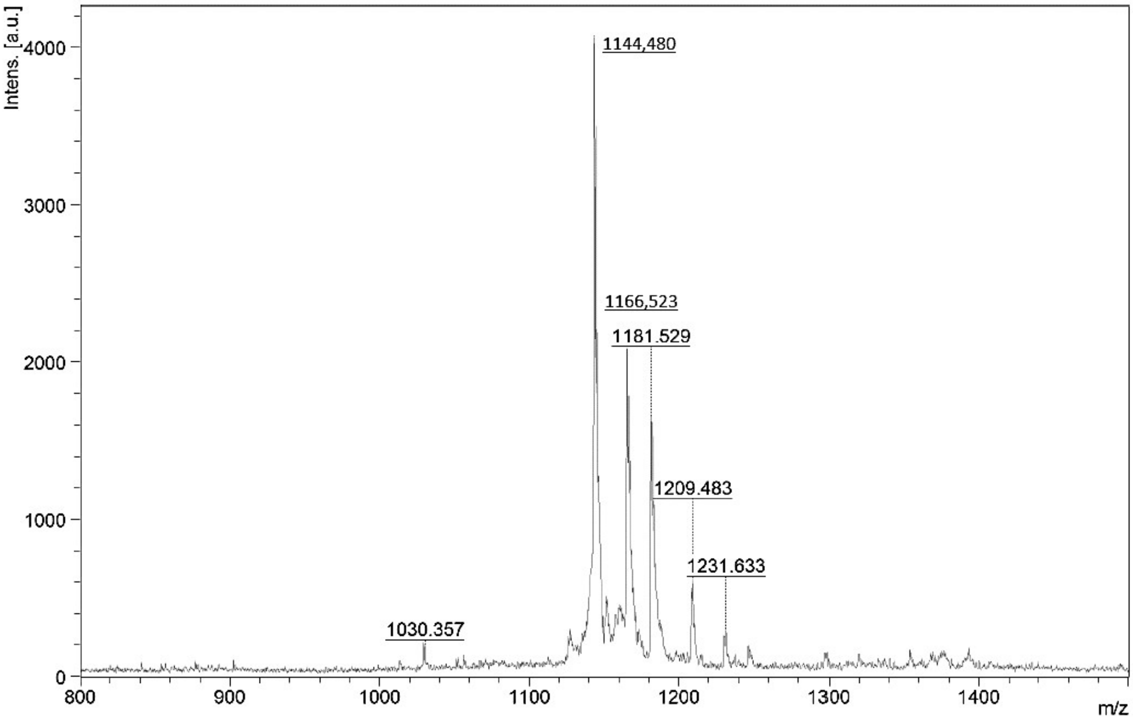

14
Mw: 1157.47
[MH+]: 1158.44

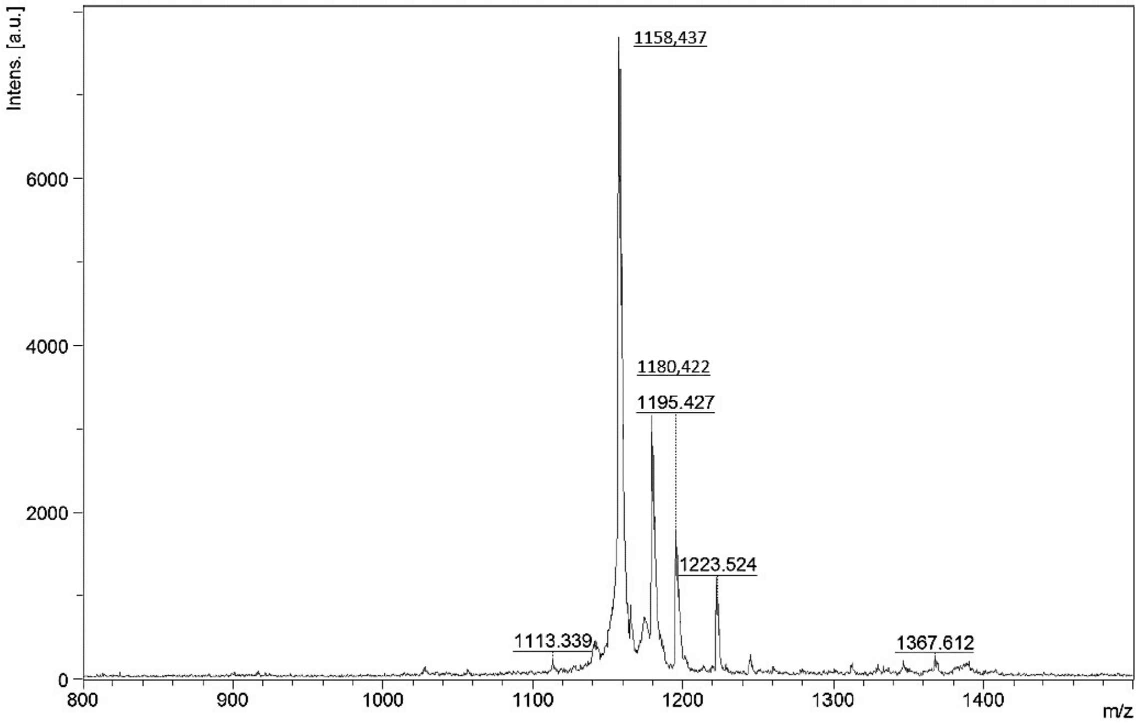

15
Mw: 1219.54
[MH+]: 1220.76

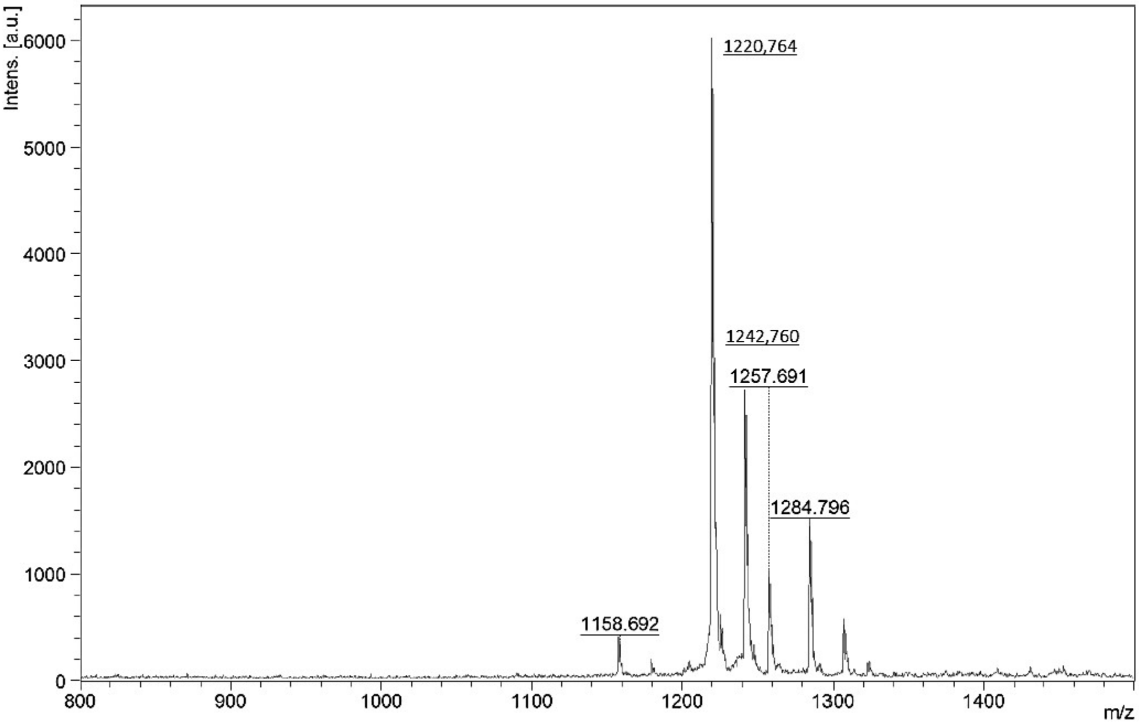

16
Mw: 1155.50
[MH⁺]: 1156.51

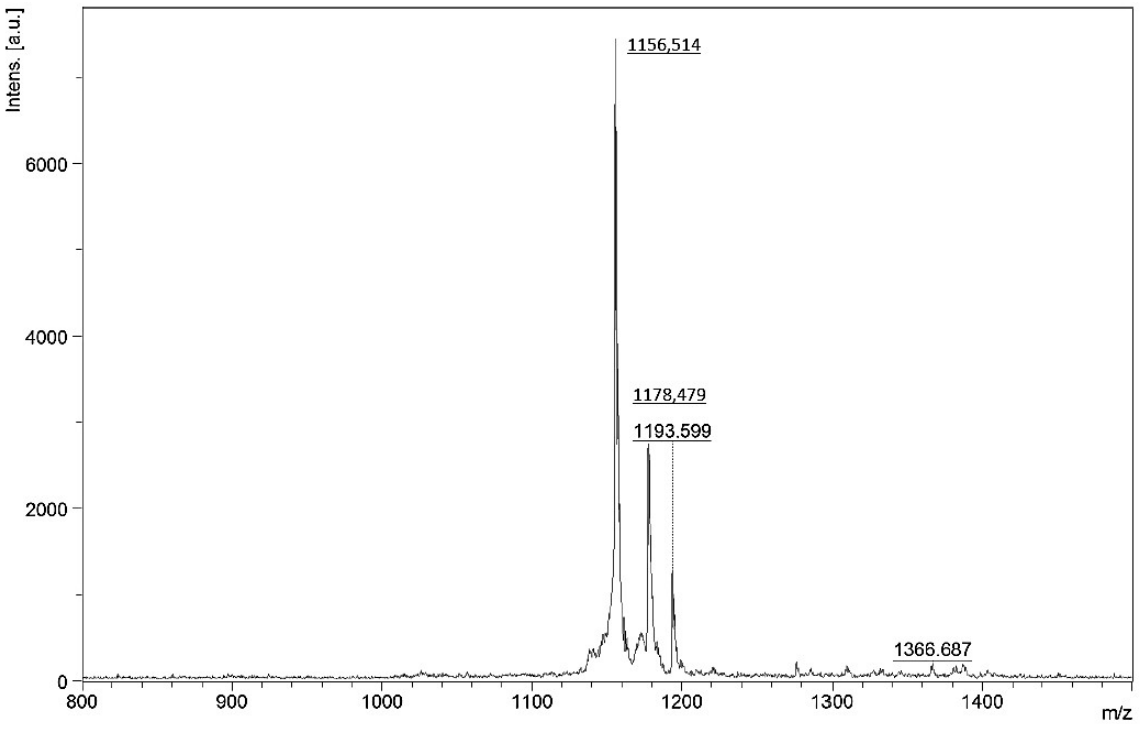

17
Mw: 1253.60
[MH⁺]: 1255.13

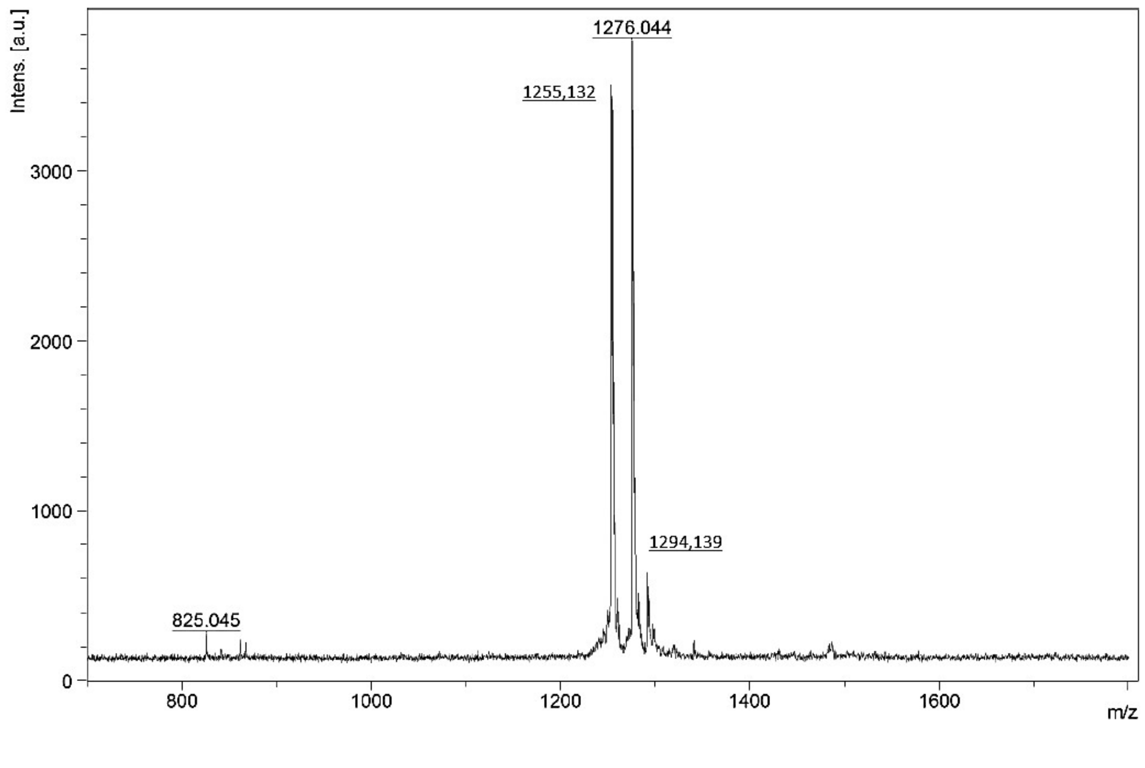

18
Mw: 1142.46
[MH$^+$]: 1143.97

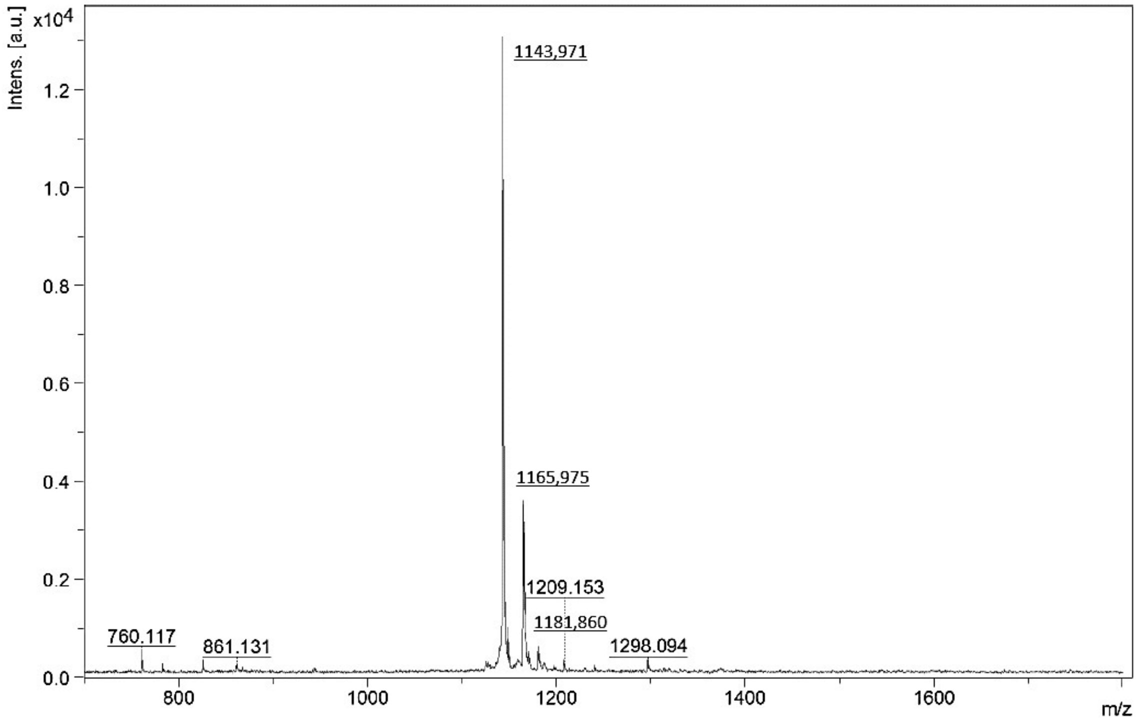

19
Mw: 1169.53
[MH$^+$]: 1170.93

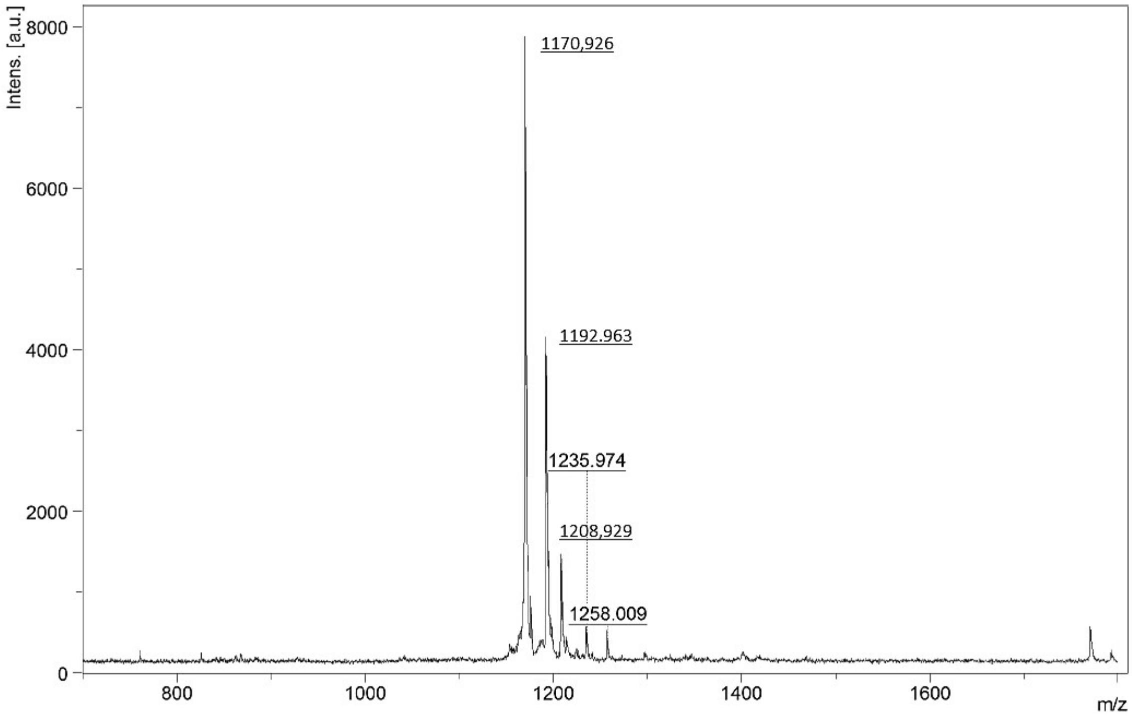

20
Mw: 1203.54
[MH$^+$]: 1204.75

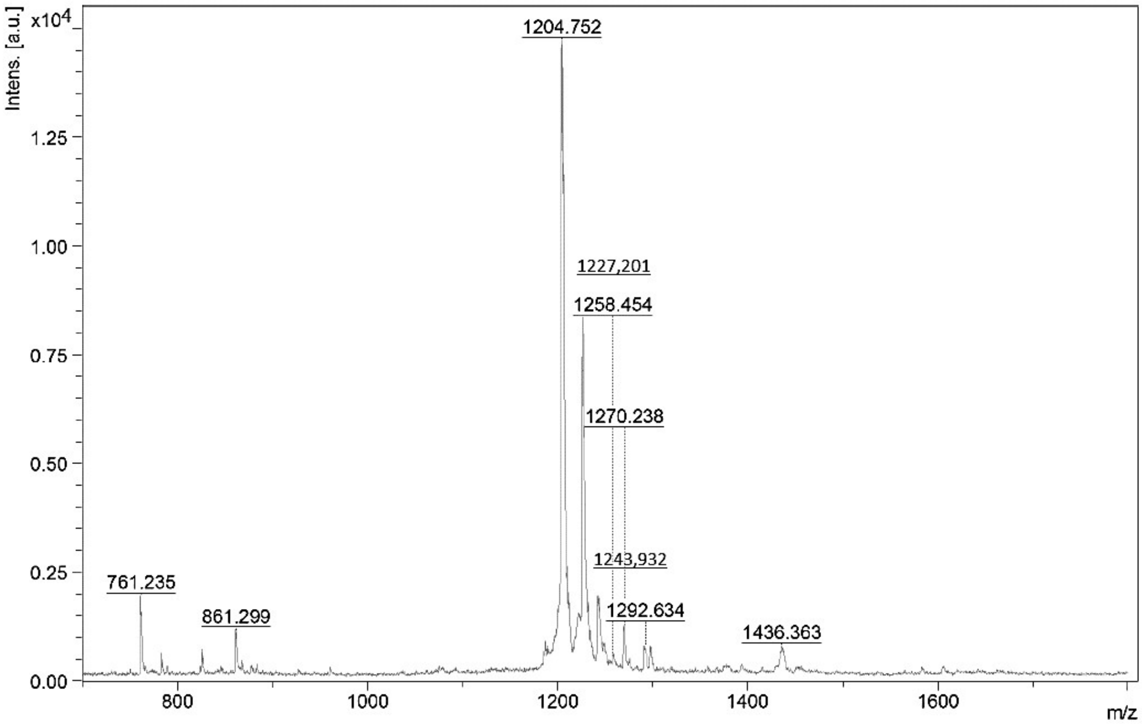

21
Mw: 1143.45
[MH$^+$]: 1145.08

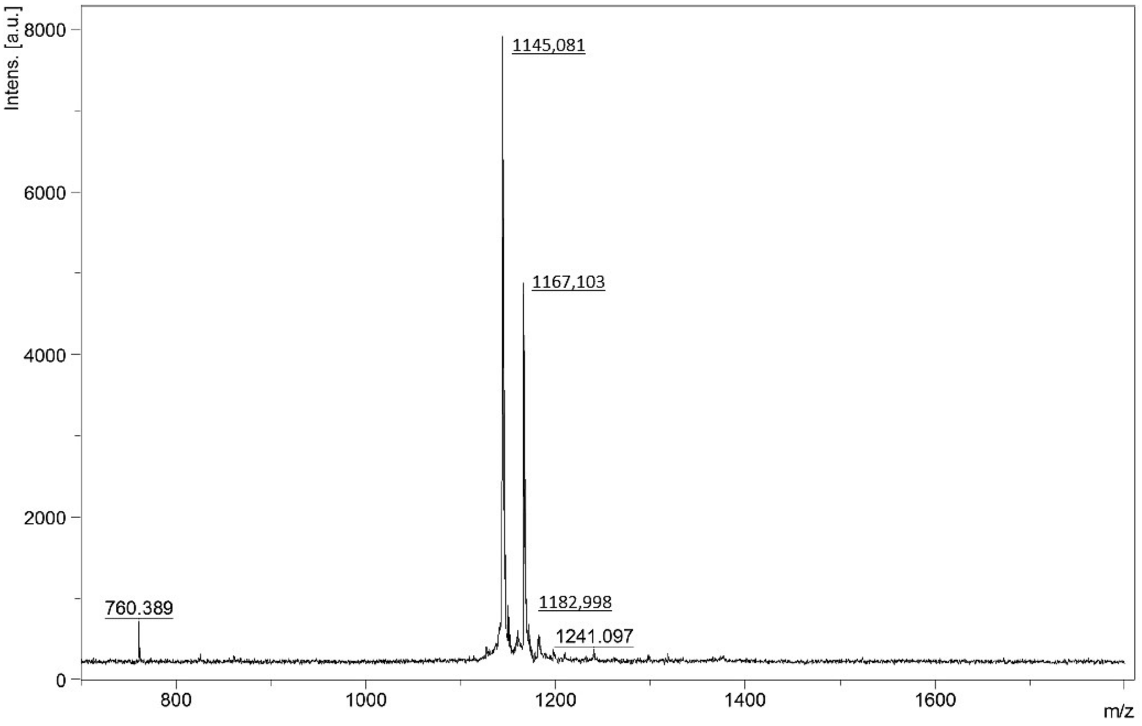

22

Mw: 1157.47

[MH$^+$]: 1158.80

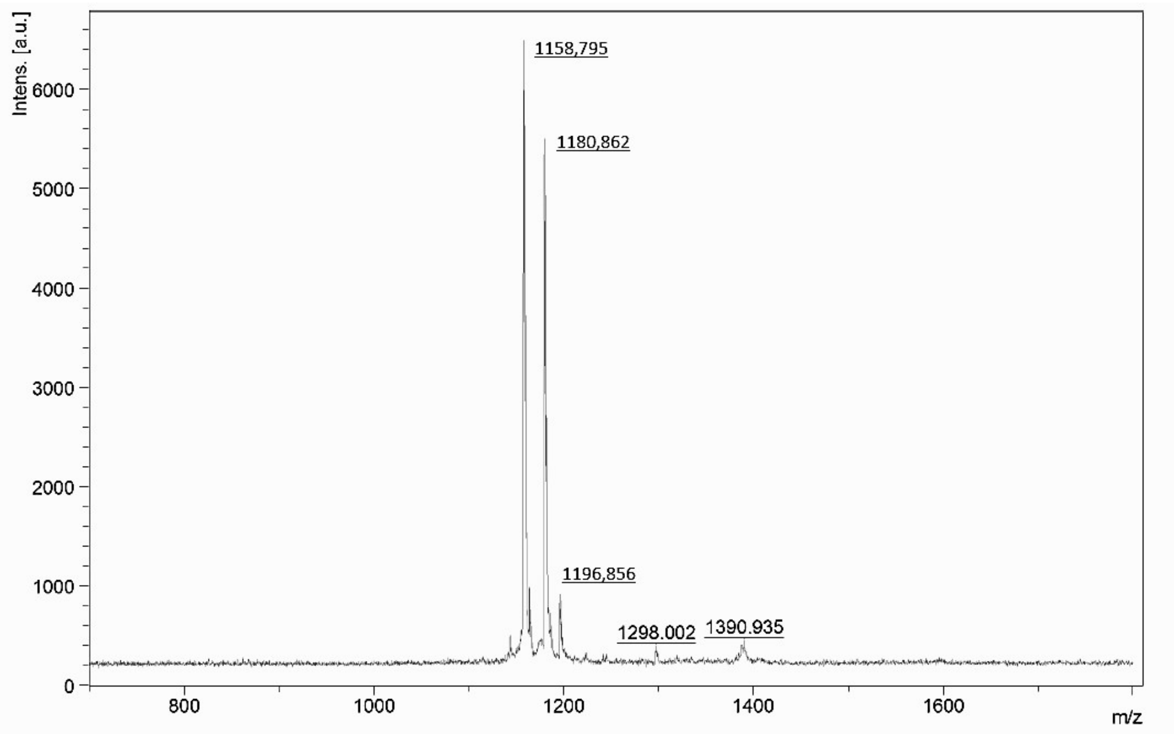

23

Mw: 1219.54

[MH$^+$]: 1220.84

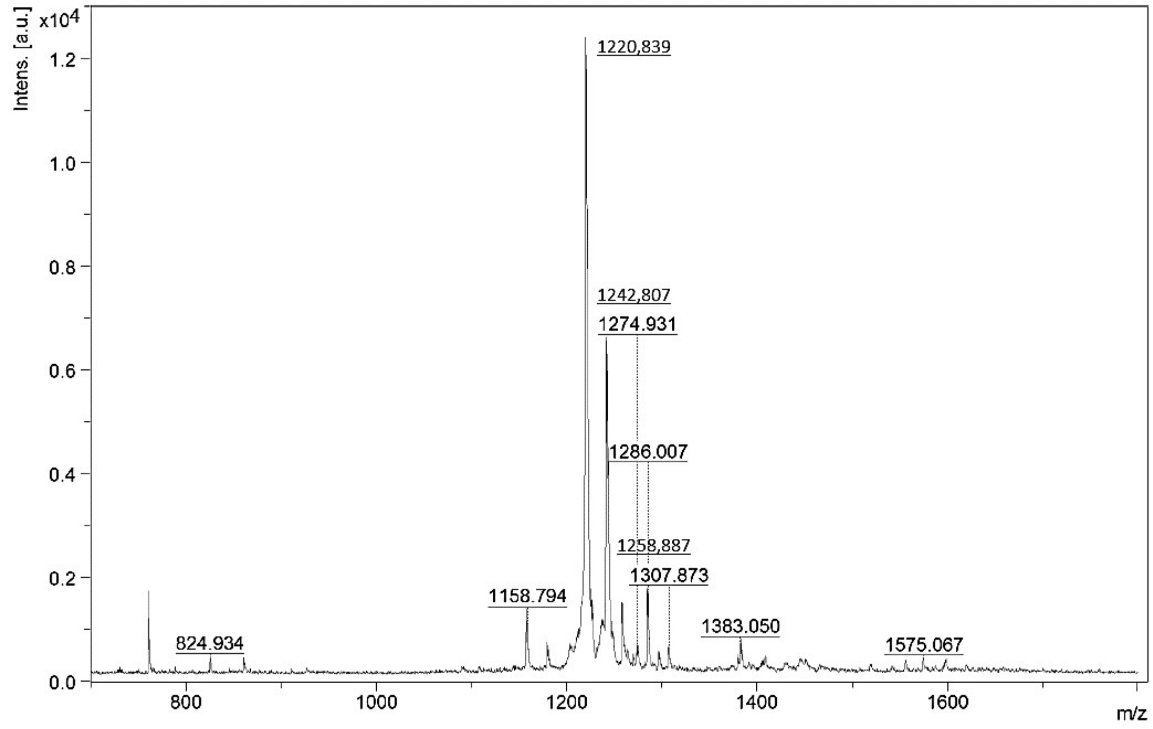

24
Mw: 1155.50
[MH+]: 1156.79

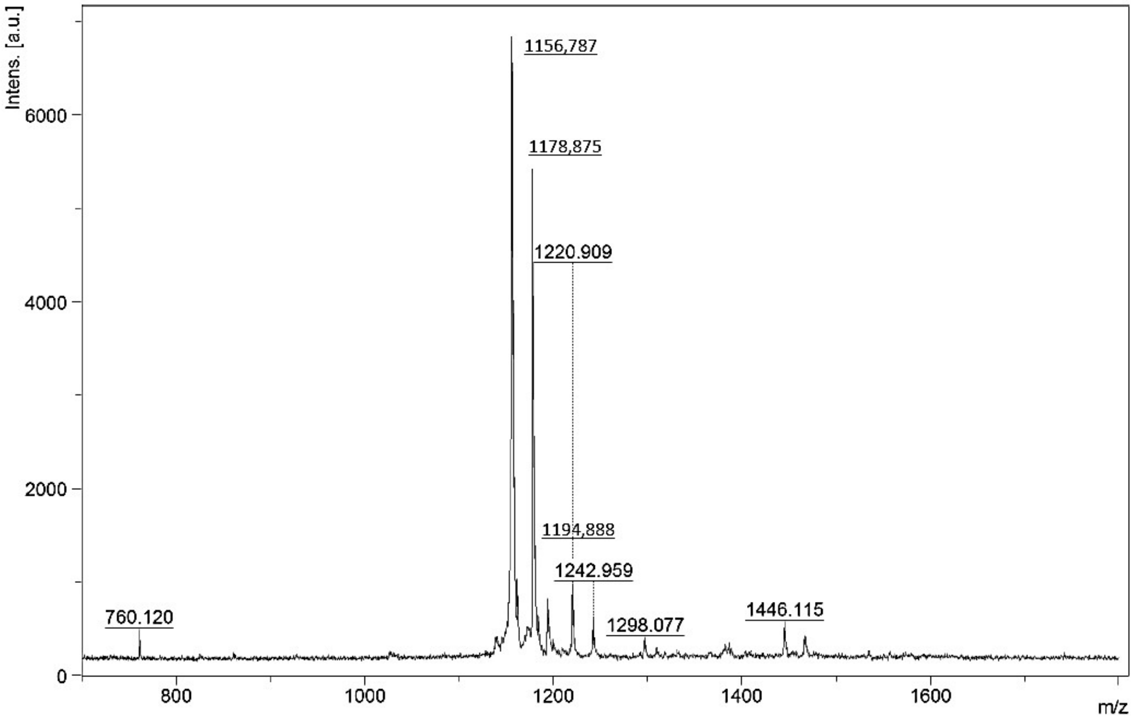

25
Mw: 1184.54
[MH+]: 1186.18

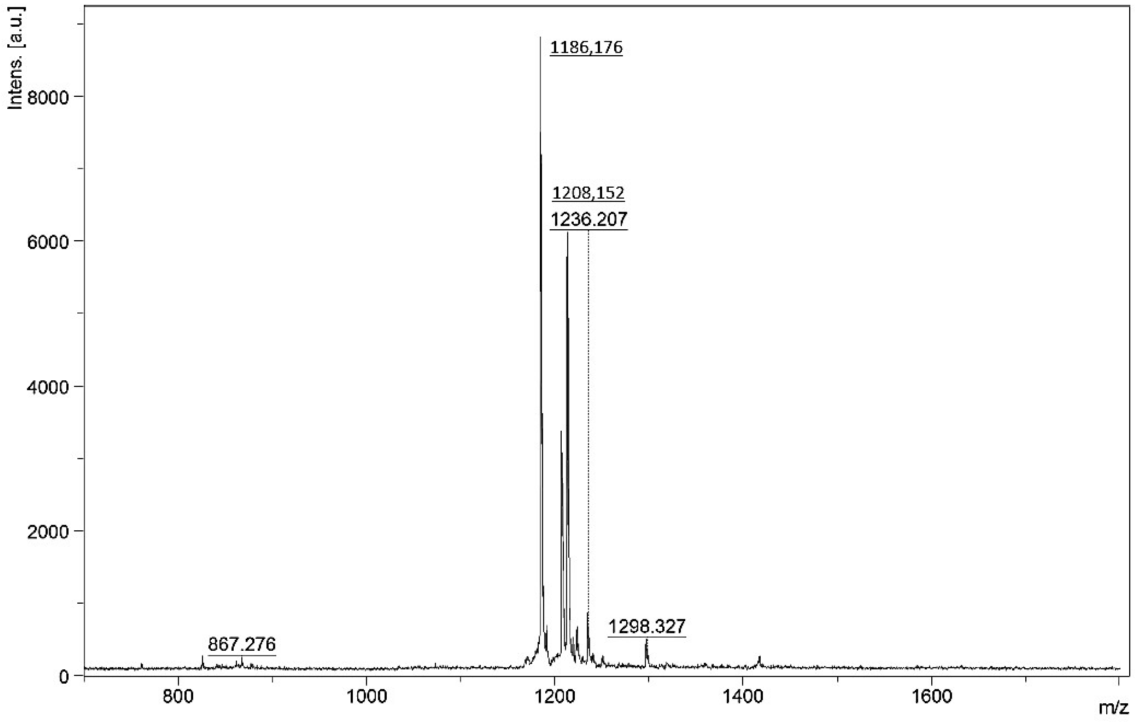

26
Mw: 1184.54
[MH$^+$]: 1186.04

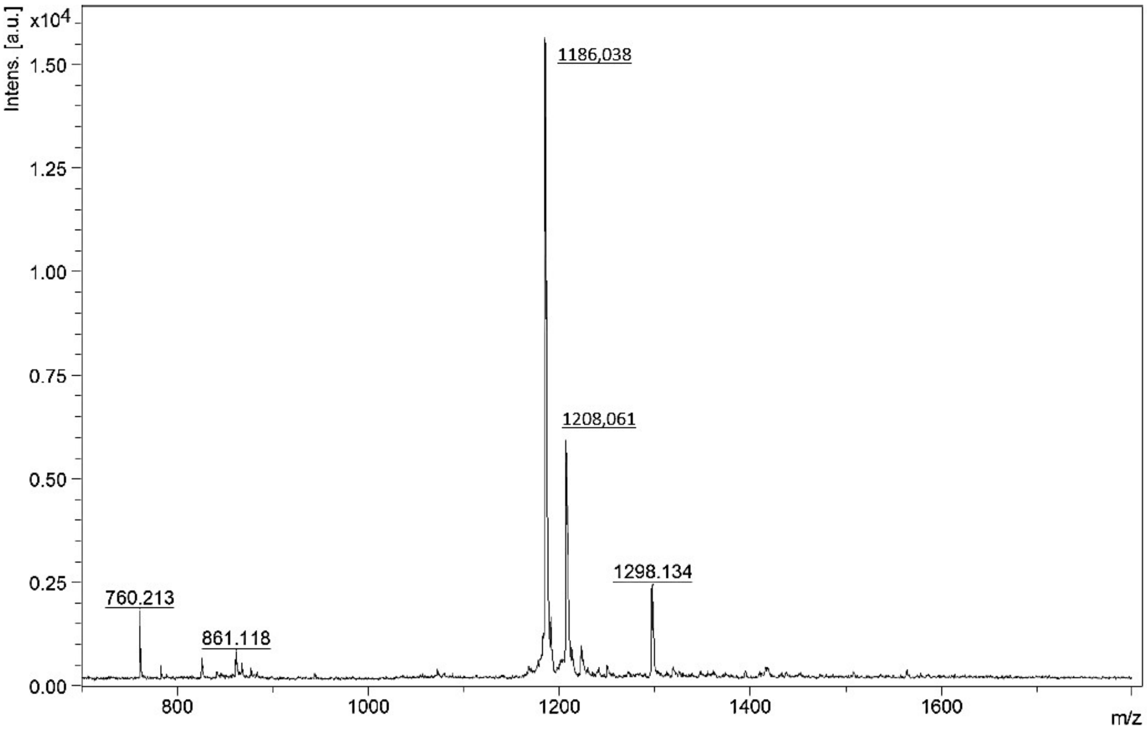

27
Mw: 1056.37
[MH$^+$]: 1057.97

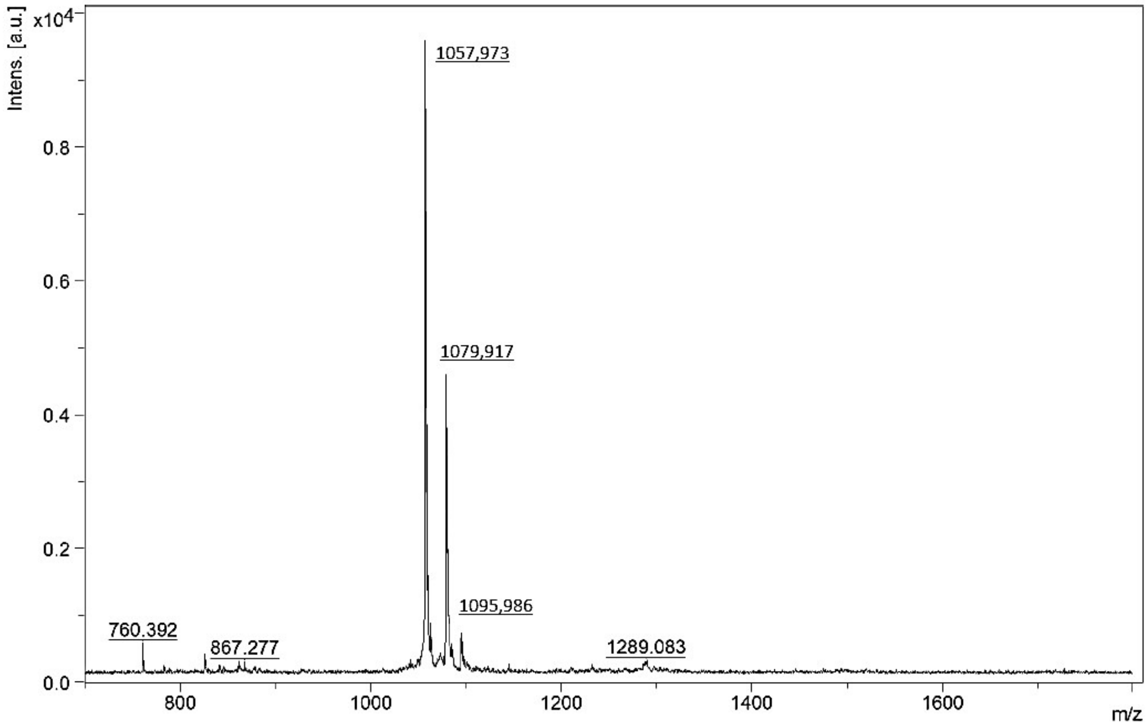

28
Mw: 928.19
[MH+]: 929.63

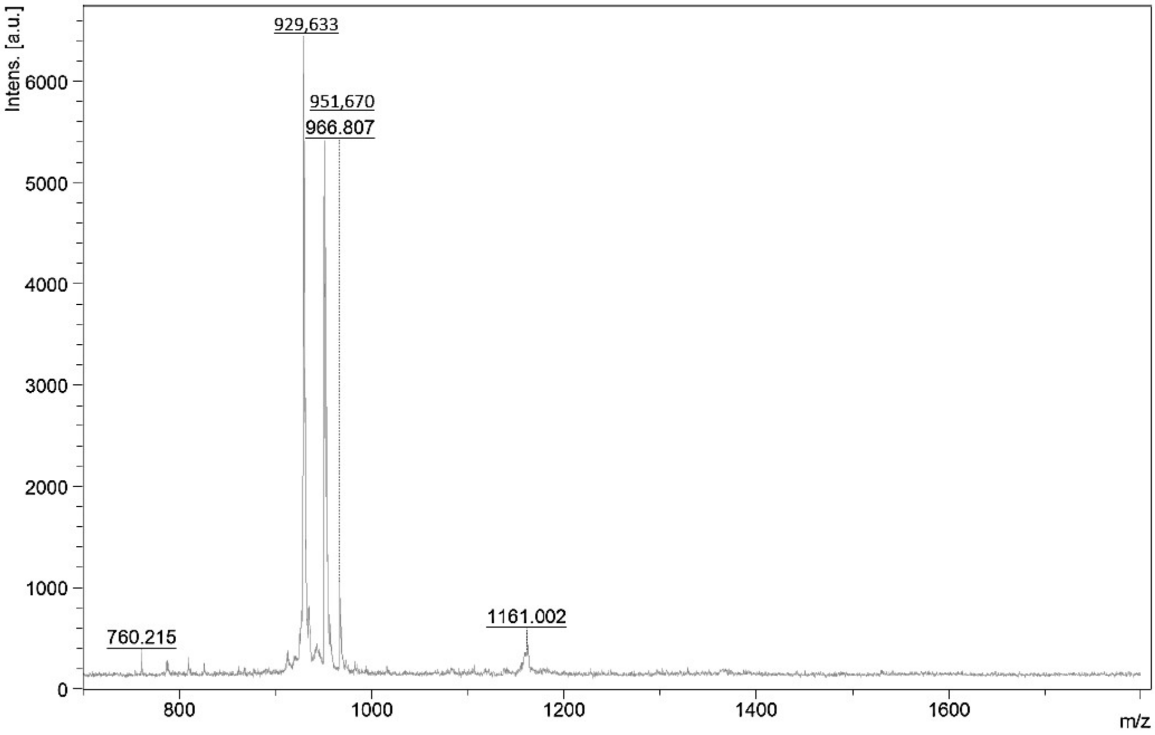

29
Mw: 730.96
[MH+]: 732.30

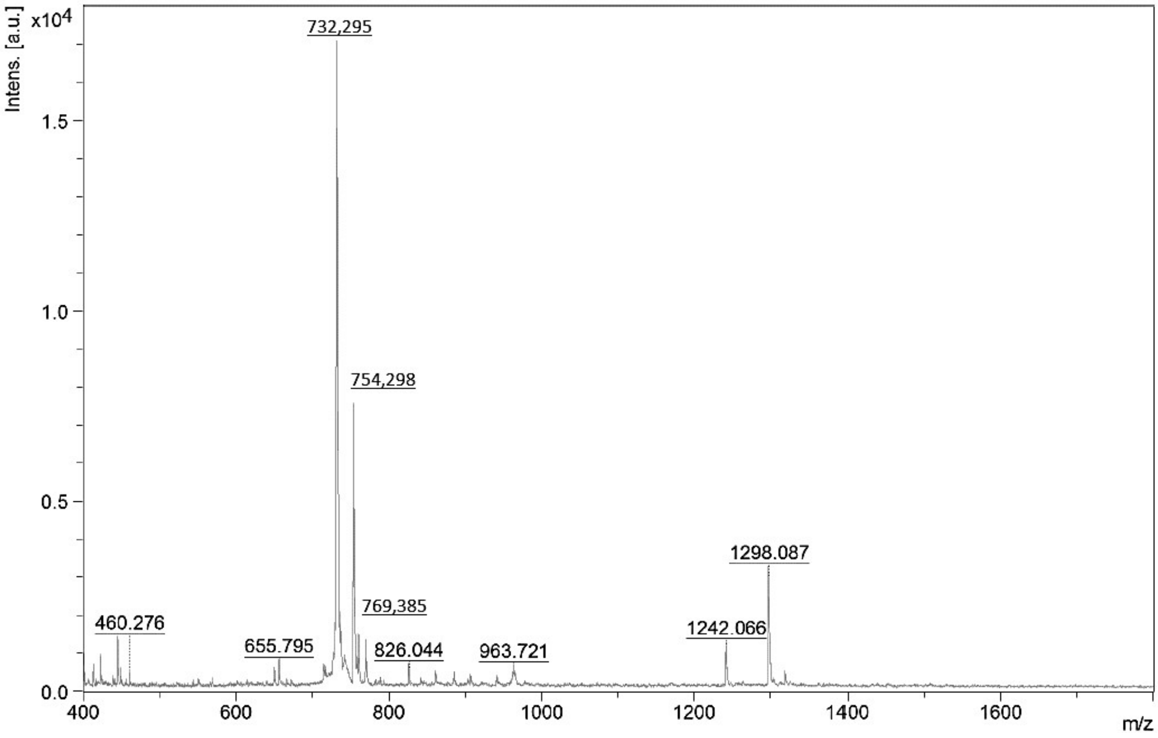

30

Mw: 1071.38

[MH+]: 1072.89

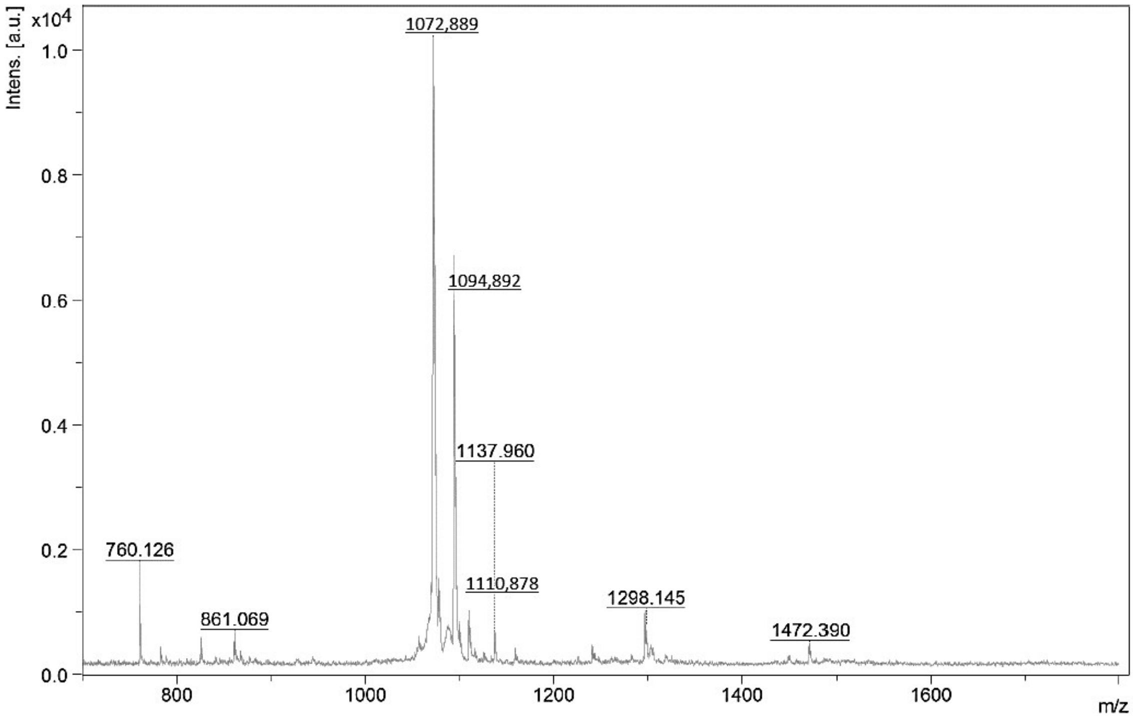

31

Mw: 943.21

[MH+]: 944.60

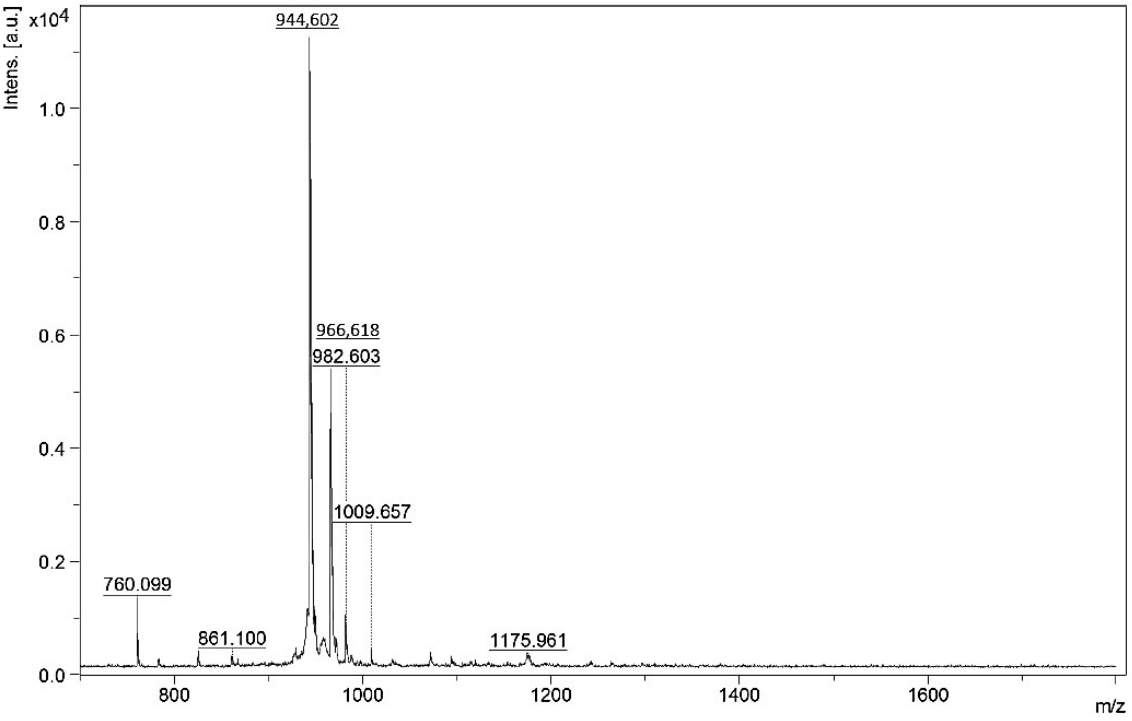

32
Mw: 745.97
[MH$^+$]: 747.30

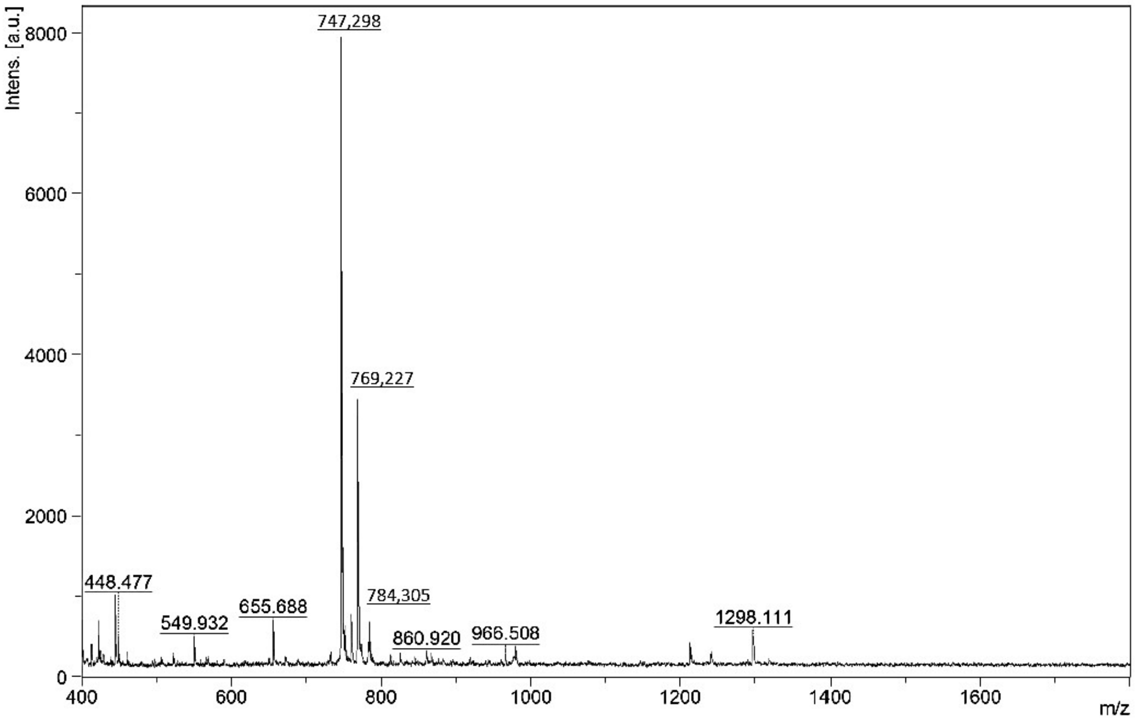

33
Mw: 1212.56
[MH$^+$]: 1213.95

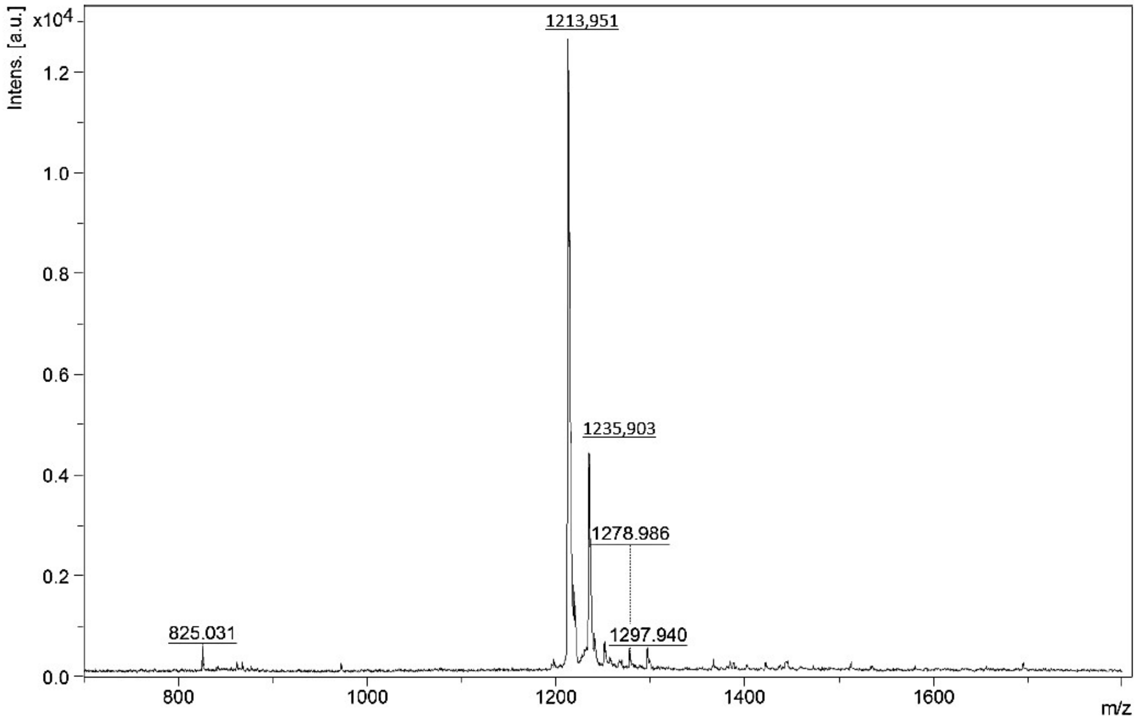

34
Mw: 1212.56
[MH$^+$]: 1213.98

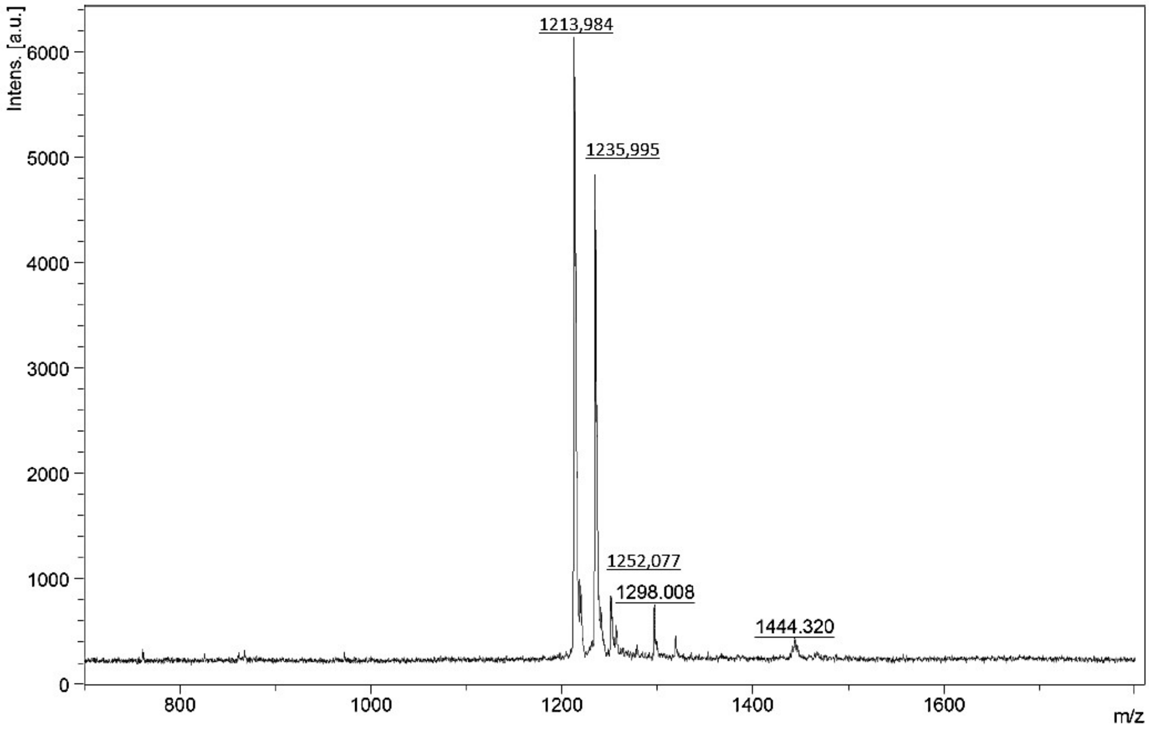

35
Mw: 1212.56
[MH$^+$]: 1214.06

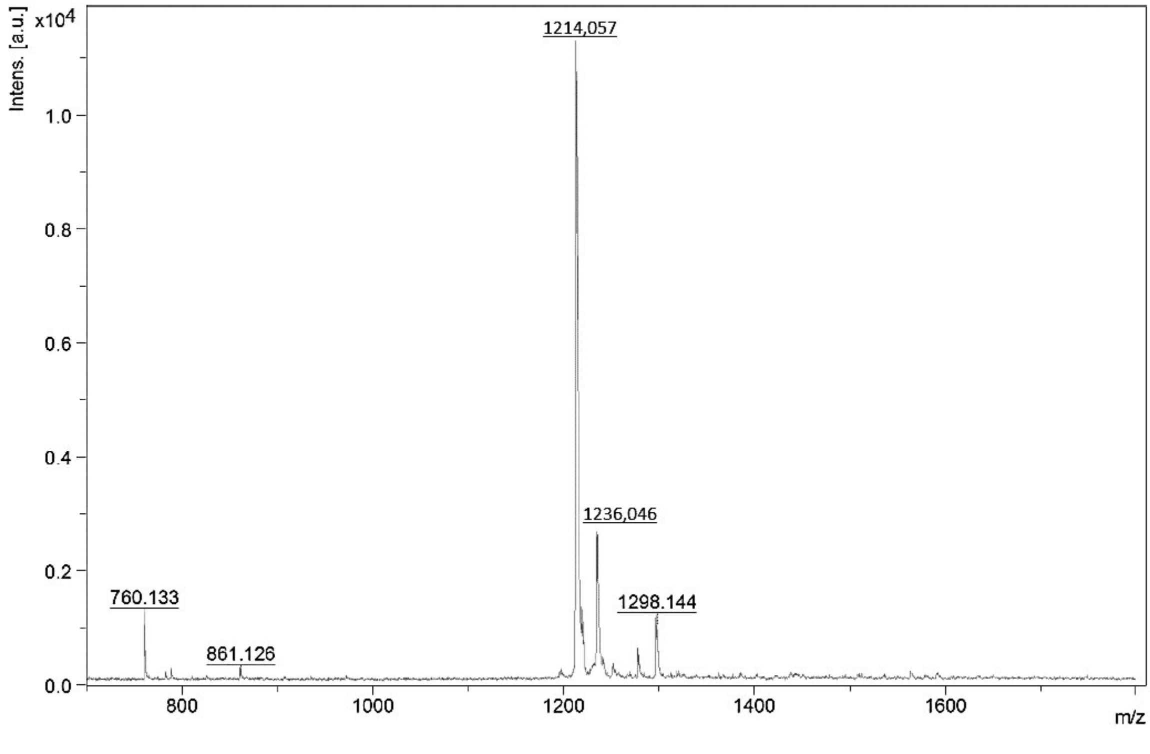

36
Mw: 1212.56
[MH$^+$]: 1214.13

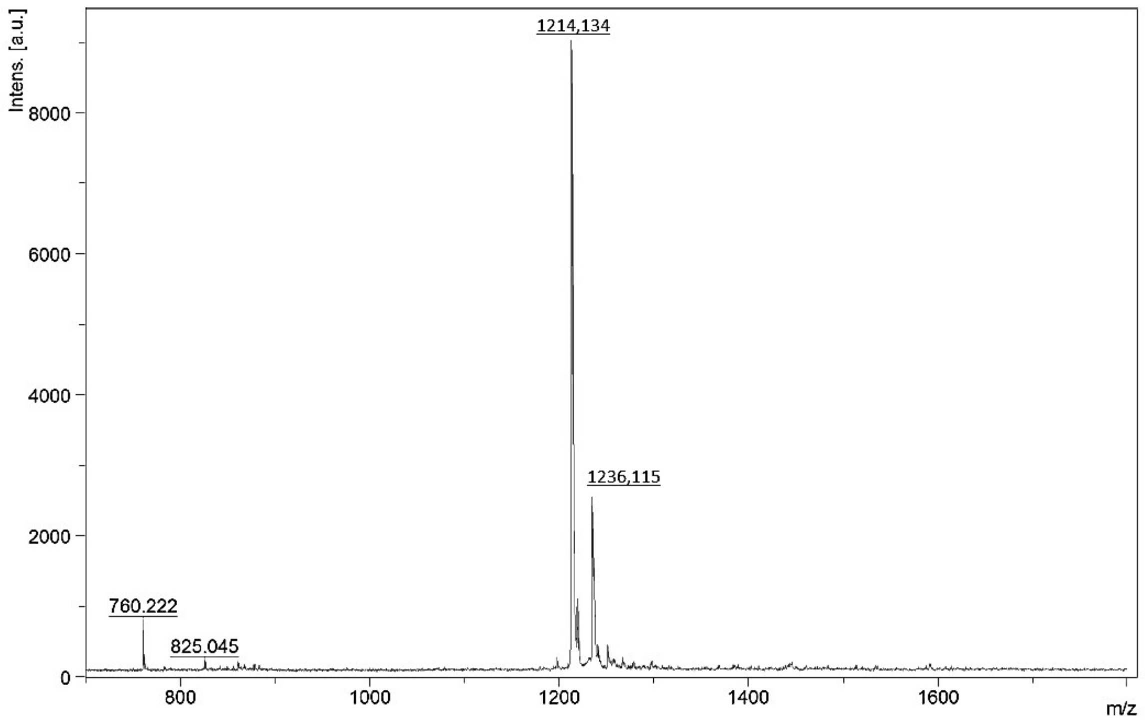

Figure S1:

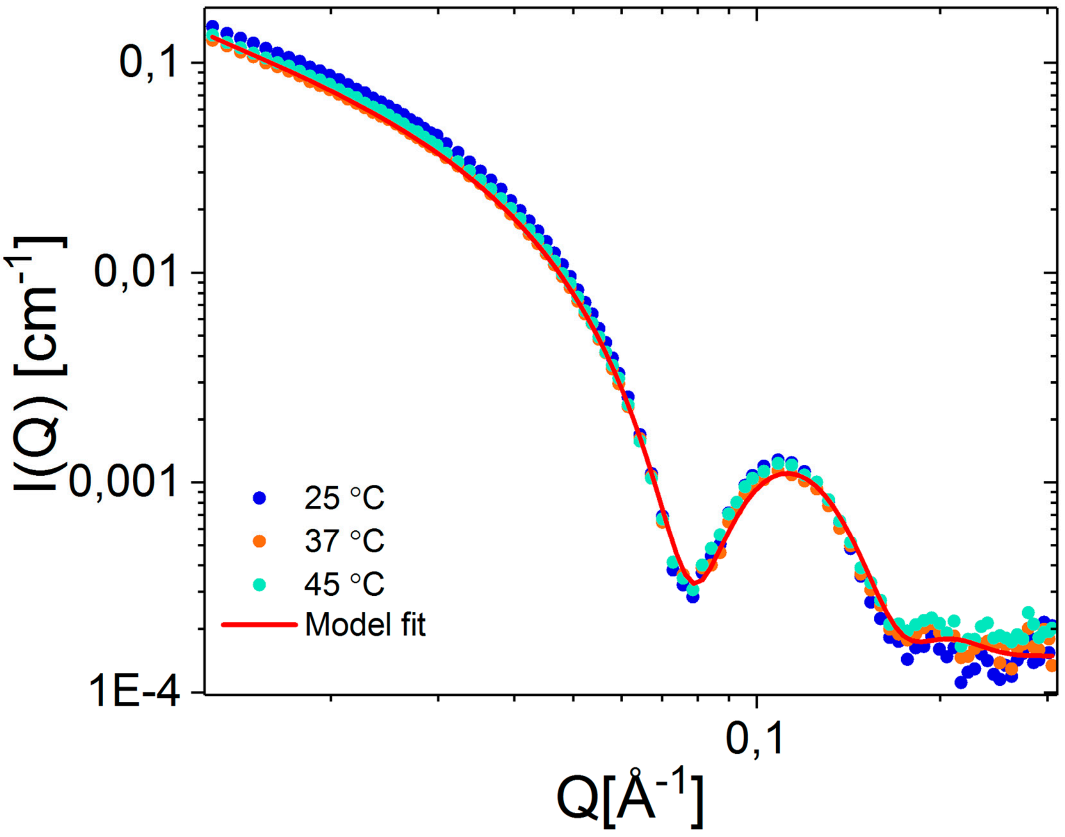

Figure S1. SAXS data for peptide 5 shows that the nanotube structure is retained over full temperature range (25-45 °C).

Figure S2:

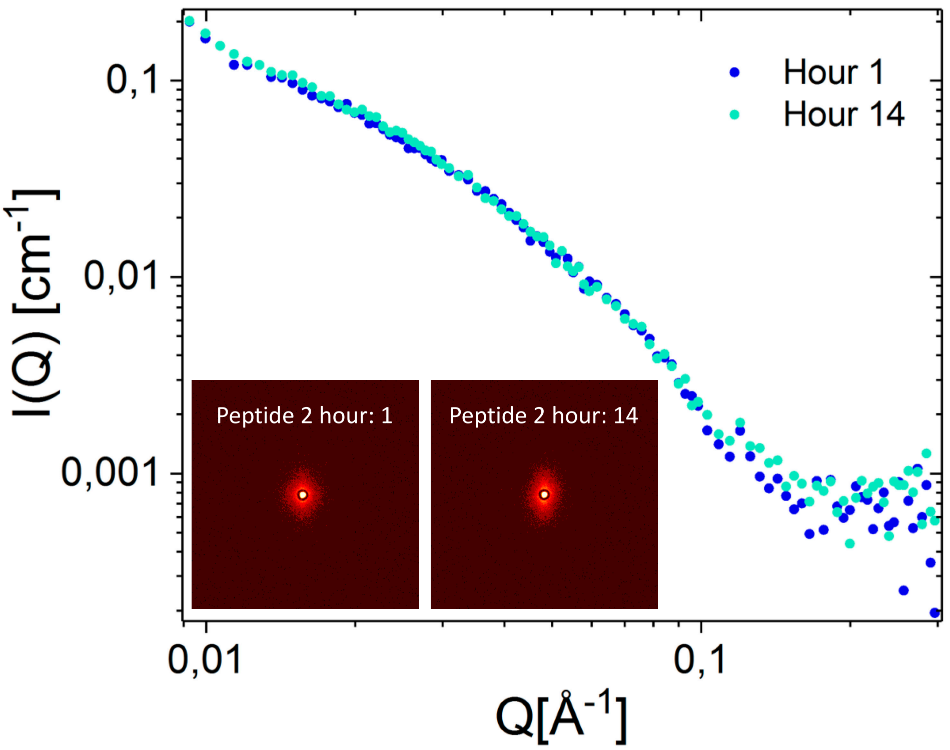

Figure S2. SAXS data for peptide 2 shows how the anisotropy (seen in the detector image) increases over time even though the scattering pattern does not change. The effect can be explained by the elongated structures being partly broken up during injection of the sample into the capillary and then reformed over time, however they are not visible in the scattering as the length of these structures are outside the measured q-range.

Figure S3:

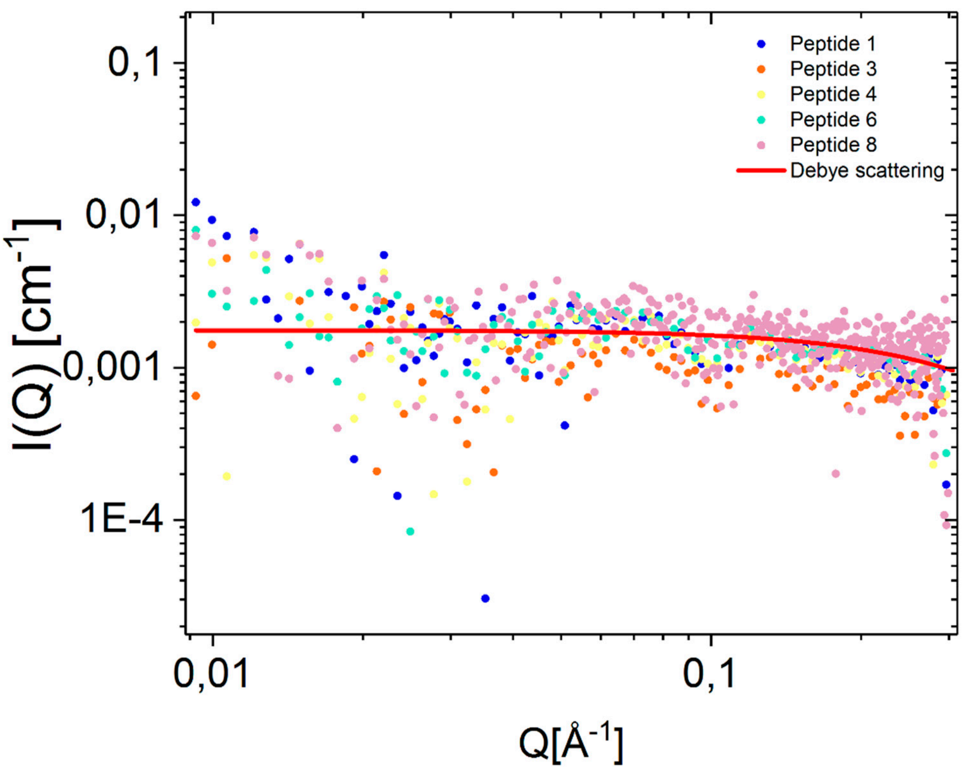

Figure S3. SAXS results showing the scattering intensity of 5 mg/mL peptides 1, 3, 4, 6 and 8 in solution measured by a Bruker Nanostar SAXS instrument and a simulated Debye curve corresponding to random coil polymer like chains.

Table S2:

Table S2. Important fit parameters from the analysis of liposomes-peptide mixes. Values outside of the experimental q-range noted with * and joint SAXS/SANS fits noted with §. The error of the fits is found to be less than 5 %.

	Liposomes	D2D peptide			Peptide 5		
**Fraction peptide**	**-**	**1:20**	**1:10**	**1:5**	**1:20**	**1:10§**	**1:5**
**ULV radius[Å]**	380	*	*	*	*	*	*
**Bilayer thickness[Å]**	38.5±0.5	38.5±0.5	38.5±0.5	38.5±0.5	38.5±0.5	37.8±0.5	37.4±0.5
**$Z_{peptide}$[Å]**	-	11	12	14	17	17	16
**$\sigma_{peptide}$[Å]**	-	3	3	3	3	3	3
**$f_{free\ peptide}$**	.	0.5	0.51	0.7	0.5	0.5	0.4
σ_{SD}	0.22	0.22	0.3	0.3	0.3	0.3	0.3
